# Not primed to agree? Short or no effect of rhythmic priming on typical adults processing number agreement

**DOI:** 10.3389/fpsyg.2025.1512267

**Published:** 2025-06-13

**Authors:** Dávid György, Douglas Saddy, Sonja A. Kotz, Julie Franck

**Affiliations:** ^1^Department of Psychology, University of Geneva, Geneva, Switzerland; ^2^Centre for Integrative Neuroscience and Neurodynamics, University of Reading, Reading, United Kingdom; ^3^Department of Neuropsychology and Psychopharmacology, Maastricht University, Maastricht, Netherlands

**Keywords:** rhythmic priming, entrainment, hierarchical structure building, music, syntactic processing

## Abstract

Accumulating evidence shows improved syntactic processing after exposure to a rhythmically regular compared to an irregular musical prime, environmental noise, or silence. One potentially shared system between musical rhythm and language processing may be responsible for the construction of hierarchical sequences. Following findings of a shorter-lived rhythmic priming effect in Jabberwocky and more precise neural tracking of linguistic constituents in natural language than in Jabberwocky, the present study hypothesized that (a) hierarchical structure building constitutes a key shared mechanism between rhythm and language processing and (b) semantic information may also play a role in structure building. In three experiments, French-speaking typical adults listened to 32-s rhythmic primes before completing six-sentence blocks of grammaticality judgment on natural language and jabberwocky materials in lab and online. Results showed a heavily reduced priming effect present only in the first sentence after a prime in Experiment 1 (natural language, online) and no priming in effects in Experiments 2 (jabberwocky, online) and 3 (natural language, in lab). Replicating previous results, overall grammaticality judgment d’ correlated with performance in a rhythm discrimination task. In two out of three experiments, grammaticality judgment performance correlated with rhythm discrimination. These correlations support the hypothesis of a domain-general cognitive network responsible for hierarchical structure building in rhythm and language processing, but do not rule out alternative accounts. However, the priming data showcase that the rhythmic priming effect is reduced when typical speakers process sentences containing linguistic information available at all levels compared to atypical populations processing natural language or typical adults processing syntactic structures in the absence of lexical semantics, and do not suggest a key role of lexico-semantic information in rhythmic priming. Furthermore, relationships between the rhythmic priming effect, rhythm discrimination, and spontaneous speech synchronization suggest that sensitivity to rhythmic priming may be influenced by several factors.

## Introduction

Rhythmicity is an ever-present component of the human experience from the molecular level, through oscillating neuronal ensembles, the heartbeat, and movement. Being perhaps two of the most uniquely human capacities, music and language both contain a certain level of rhythmicity, though the two differ both in their surface periodicity and underlying metrical structures. Whether music and language processing constitute two independent systems (Ayotte et al., 2002; Peretz and Coltheart, 2003; Peretz, 2009) or two systems with shared resources (Patel, 2003, 2011, 2012, 2014; Patel and Morgan, 2016; Ozernov-Palchik and Patel, 2018) is a matter of much debate. Some recent work has specifically focussed on a potential overlap between musical rhythm and language processing.

Several cortico-subcortical brain circuities have been identified as potentially shared networks between rhythm and language processing (Kotz and Schwartze, 2010; Fujii and Wan, 2014; Kotz et al., 2014; Patel and Iversen, 2014; Tierney and Kraus, 2014; Schwartze and Kotz, 2016; Heard and Lee, 2020). Further, the precise encoding of low-level information in the acoustic signal, neural oscillations entraining to external and other internal oscillations, and hierarchical cognitive control are considered as shared cognitive markers of rhythm and language processing (Ladányi et al., 2020; Asano et al., 2021; Fiveash et al., 2021; Nayak et al., 2022).

Empirical evidence supporting an overlap between rhythm and language processing comes from a series of various experiments. Correlations have been found between pre-school children’s ability to synchronize motor production to an external rhythm and the precision with which their brain encodes the speech syllable envelope, as well as reading readiness measured by phonological awareness, auditory short-term memory, and rapid object and color naming (Woodruff Carr et al., 2014). In school-age children, the ability to tell whether two rhythmic structures are the same or different is linked to morphosyntactic production in 6-year-old children and syntactic comprehension in children aged 7–17 (Gordon et al., 2014, 2015; Lee et al., 2020; Persici et al., 2023).

Typical adults with high rhythmic skills have also been reported to outperform adults with lower rhythmic abilities in sentence-in-noise perception, suggesting that rhythmic patterns may provide cues implying certain grammatical structures (Slater and Kraus, 2015; Yates et al., 2019). Furthermore, fMRI evidence shows overlapping brain regions involved in rhythm and syntax processing, including the left inferior frontal gyrus (pars opercularis), the left supplementary motor area, as well as the bilateral insula (Heard and Lee, 2020). As to the specific (hierarchical) structural similarities between rhythm and language, typical adults also subjectively group metronome beats into binary groups (Poudrier, 2020), while neuroimaging evidence shows neural entrainment to structurally relevant frequencies absent from language or musical stimuli (Ding et al., 2015; Large et al., 2015; Tal et al., 2017; Kaufeld et al., 2020; Glushko et al., 2022; Criscuolo et al., 2023), lending support to hierarchical sequence processing as a potential shared mechanism between rhythm and language (Martins et al., 2017; Martins et al., 2020; György et al., 2024).

Manipulating a rhythm presented before the speech stimulus or within the speech stimulus can also influence language processing in numerous populations, such as children with developmental language disorder (DLD) and developmental dyslexia (DD), typically developing children, typical adults and adults with acquired neurodegenerative disorders or lesions to the basal ganglia. Some of these experiments reported that creating an overly regular trochaic speech rhythm in German can influence semantic processing in adults, and syntactic processing and both typical adults and those with lesions to the basal ganglia (Rothermich et al., 2012; Roncaglia-denissen et al., 2013; Rothermich and Kotz, 2013; Roncaglia-Denissen et al., 2014; Kotz and Schmidt-Kassow, 2015). Other studies found that cueing sentences or phrases with a beat pattern that matches (as opposed to mismatches) their syllabic rhythm can improve phonological processing in adults (Cason and Schön, 2012; Cason et al., 2015a, 2015b).

Of crucial importance to the present study are reports of a general rhythmic priming in effect (RPE) according to which exposure to a regular prime whose musical rhythmic structure is easy to extract, leads to enhanced subsequent syntactic processing compared to exposure to a less regular prime, environmental noise, or silence children with typical development, developmental dyslexia, or developmental language disorders as well as typical adults in French, English and Hungarian (Przybylski et al., 2013; Schön and Tillmann, 2015; Bedoin et al., 2016, 2018; Chern et al., 2018; Canette et al., 2019, 2020b; Canette et al., 2020a; Fiveash et al., 2020; Ladányi et al., 2021; György et al., 2024). No RPE was reported in mathematical and visuo-spatial control tasks in typically developing English children, semantic fluency in French children (Canette et al., 2020b), and a picture naming task in Hungarian children (while a Stroop-task showed a lower Stroop effect after an irregular than after a regular prime or baseline, see Ladányi et al., 2021). This suggests that the rhythmic priming effect can be described as a language-specific effect realized by shared rhythm and language processing systems rather than a simple motivational effect due to a more engaging musical stimulus.

Recently, Kim et al. (2024) reported no RPE in 7-12-year-old English-speaking children performing a grammaticality judgment and a thematic role sentence comprehension task. As this experiment tested older English-speaking children than (Chern et al., 2018), the authors proposed that age may play a role in rhythmic priming effects. However, as earlier studies report a RPE in typically developing French-speaking children of the same age range, it seems that neither age nor the target language alone can account for differences in finding a RPE.

Moreover, recent work has found a short-lasting rhythmic priming effect in typical adults processing Jabberwocky sentences. Rather than a typical RPE on 6 sentences after priming, György et al. (2024) reported an RPE for the first three sentences only. Due to the use of a block design (Exp 1) and no significant difference between a silent (baseline) prime and either of the two rhythmic conditions, the direction of the RPE in typical adults remained unclear other than indicating a higher performance after a rhythmically regular than a rhythmically irregular musical prime. In other words, the authors reported a replicable RPE for the first three sentences after priming, but whether this difference between the regular and irregular primes stems from a facilitatory effect of the regular prime or a penalizing effect of the irregular prime remained unanswered. One potential explanation for the shorter RPE is the use of Jabberwocky sentences. Specifically, the authors suggested that the absence of lexico-semantic information may play a role in structure building processes influenced by the rhythmic priming effect. If lexico-semantic processing is involved in syntactic structure building and rhythmic priming is realized through a shared system engaged in hierarchical structure building, removing lexico-semantic information from speech may reduce the effectiveness of rhythmic priming, resulting in a weaker or shorter effect. This suggestion is in line with findings by Kaufeld et al. (2020), who reported that neural entrainment to syntactic constituents is weaker in Jabberwocky than in natural Dutch sentences. The authors argued that the tracking of the speech signal is enhanced when meaningful linguistic units can be inferred, suggesting that oscillatory activity might reflect the generation of inference-based linguistic representations (Martin and Doumas, 2017).

## The present study

The present study sought to replicate György et al.’ (2024) work, using natural language minimal pairs to evaluate to what extent the lack of lexico-semantic information can explain their initial results. Similar to György et al. (2024), two types of effects were investigated: the immediate effect of rhythmic priming on subsequent syntactic processing and correlations between rhythm and language processing.

Following the authors’ reasoning, if a system responsible for hierarchical structure building constitutes a key shared component between rhythm and language processing (Hypothesis 1), we expected that a regular rhythmic prime would result in higher grammaticality judgment performance than an irregular prime or a silent control condition (Prediction 1). It would also be possible that a fully developed language system may show less improvement due to a regular prime, and be more susceptible to disruption by an irregular prime. Given the lack of conclusive results regarding the direction of the RPE in typical adults, the present study included a silent control condition next to rhythmically regular and irregular primes.

If lexico-semantic information is involved in the structure-building processes through which rhythmic priming is realized (Hypothesis 2), we expected to observe a RPE over 6 sentences (as reported in children with typical and atypical development, Przybylski et al., 2013; Bedoin et al., 2016, 2018; Chern et al., 2018; Canette et al., 2020b; Ladányi et al., 2021) rather than a shorter 3-sentence effect as reported by György et al. (2024) (Prediction 2). To test this hypothesis, the present study used natural language stimuli that were constructed as minimal pairs to those employed by György et al. (2024). As such, ungrammatical sentences in the present study also involved agreement errors, particularly because the presence of an attractor noun phrase triggered a substantial number of errors in sentence production, as seen in examples like ‘The label on the bottles are rusty’ (Bock and Miller, 1991, and subsequent studies). Sensitivity to attraction was also observed in sentence comprehension, where it manifested as a ‘grammatical illusion.’ This illusion refers to the perception that a sentence with an agreement violation is correct when an attractor matches the verb in terms of its agreement features (Wagers et al., 2009; Villata et al., 2018; Villata and Franck, 2020). As such, agreement attraction phenomena allow measuring if participants built the correct (syntactic) hierarchical structure during sentence comprehension (Franck et al., 2006, 2010, 2015; Franck and Wagers, 2020).

Like in György et al. (2024), long-term relationships between rhythm and language were investigated by examining correlations between participants’ accuracy in grammaticality judgments and their performance on behavioral tests of rhythm and beat discrimination using the rhythm and accent subtests from the short version of the standardized Profile of Music Perception Skills (PROMS, Law and Zentner, 2012; Zentner and Strauss, 2017). If a shared cognitive system is responsible for hierarchical structure building in both linguistic and non-linguistic domains, we predicted that grammaticality judgment accuracy would correlate strongly with rhythm and beat discrimination skills. We further hypothesized that participants’ sensitivity to rhythmic regularity in a grammaticality judgment task (measured as the difference in performance following a regular versus an irregular prime, or the rhythmic priming effect) would correlate with their ability to process rhythmic structures (Gordon et al., 2014, 2015; Lee et al., 2020; Persici et al., 2023).

The rhythmic priming effect in typical adults is reported to be shorter (and sometimes weaker in typically developing children) than that in vulnerable populations. However, it appears to be related to rhythm discrimination, auditory selective attention, as well as frequency of listening to music, tendency to tap to a rhythm, and seeing music as a social bond (Canette et al., 2019; György et al., 2024). To further explore the link between RPE and rhythmic abilities, the present study also used a French version of the spontaneous speech synchronization task developed by Assaneo et al. (2019). Disguised as a syllable discrimination task, this protocol measured whether participants synchronize their speech output to an external rhythm. In this task, participants listened to a series of syllables and whispered the syllable ‘tah’ at the same time to make the task more difficult. Rather than their responses to ‘did you hear syllable X?’ type questions, the actual dependent variable was the phase-locking-value, representing the degree to which the participants synchronized their whisper rate to the presentation rate without overt instructions. As such, this task served as a measure of (non-hierarchical) rhythmic behavior. Spontaneous speech synchronization is related to performance in word learning tasks and neural encoding of syllable information (Assaneo et al., 2019). We aimed to explore whether participants’ tendency to synchronize to an external rhythm also plays a role in their sensitivity to rhythmic priming.

In summary, the hypotheses and predictions of the present study are as follows:

- A cognitive system responsible for coding hierarchical sequences constitutes an overlap between rhythm and language processing.
- Rhythmic priming will influence subsequent performance in a grammaticality judgment task such that it will be higher after a regular prime than after an irregular prime (with a silent baseline equal to irregular and lower than regular if the effect is primarily facilitatory, and equal to regular and greater than irregular if it is primarily penalizing).
- Overall grammaticality judgment accuracy will correlate with the ability to discriminate rhythmic structures.
- Sensitivity to the regularity of the prime (i.e., the rhythmic priming effect) will correlate with the ability to discriminate rhythmic structures.
- Is sensitivity to rhythmic priming linked to the likelihood with which a participant synchronizes their speech output rate to an external presentation rate?
- Lexico-semantic information reinforces the structure-building processes through which the RPE is realized.
- The duration or size of the RPE will be greater in natural language stimuli than in Jabberwocky.

Due to the sanitary restrictions during the Covid-19 Pandemic, Experiment 1 of the present study had to be carried out online. Given the difference between the present results and those reported by György et al. (2024), Experiments 2 and 3 were conducted to ensure that the modality of testing (online vs. in lab) did not constitute a confound variable. As such, Experiment 2 attempted to replicate György et al.’ (2024) Experiment 2 online, while Experiment 3 mirrored Experiment 1 in a controlled laboratory setting. Results from the three experiments will be reported one by one in the following sections.

## Experiment 1

### Methods

#### Participants

117 native French-speaking typical adults (87 women) participated in the experiment. Participants were between 18 and 34 years of age (*M* = 20.55, SD = 2.57), reporting no history of neurological disorders, specific language impairment, amusia, or psychiatric issues.

Participants gave informed consent prior to the start of the experiment, were not made aware of the purpose of the study, and compensated with either course credits or 20 CHF for their time. The experiment was approved by the University of Geneva Research Ethics Committee (PSE.20191004.04).

#### Materials

##### Grammaticality judgment (GJ) task

***Musical stimuli***: The two 32-s rhythmic primes (1 regular and 1 irregular) and the 32-s-long silence condition used in this experiment were identical to those used by György et al. (2024), the rhythmic primes adapted from Przybylski et al. (2013). The two primes contained the same number of tones and both featured a tam-tam at 175 Hz and a maracas at 466 Hz. The regular and irregular primes were different in the ease with which their underlying musical rhythmic structure was easy (regular prime) or difficult (irregular prime) to extract. The metrical structure of the regular rhythm consisted in temporal constituents of 125 ms, 250 ms, 500 ms and 1,000 ms. Additionally, the two instruments played simultaneously on six of the eight beats of the regular pattern. While the irregular prime also contained regular intervals of 125 ms, the lack of simultaneity between acoustic events created a less easily extractable hierarchical structure.

***Linguistic stimuli***: A total of 144 Jabberwocky sentences from György et al.’ (2024) study were converted into fully natural language sentences. The syntactic structures of the sentences included simple subject-verb-object sentences (24), subject relatives (12), complement clauses (12), common (object-subject-verb) object relatives (72), and object relatives with stylistic inversion (object-verb-subject) (24). Half the sentences were grammatically correct (*N* = 72) and the other half incorrect (*N* = 72). Every sentence contained a verb and 2 noun phrases (NP) mismatching in grammatical number, one of which was the grammatical subject of the verb while the other was an attractor. If the subject was in the plural, the attractor was in the singular, whereas if the subject was in the singular, the attractor was in the plural. A subject-verb number agreement violation where the verb agreed to the attractor NP instead of the subject NP constituted the ungrammaticality in all ungrammatical sentences. Table 1 shows an overview of ungrammatical sentences of all 5 structures. Please see Appendix 1 for a complete list of the linguistic stimuli.

**Table 1:** Examples of ungrammatical sentences of the five syntactic structures, respectively.

Sentence structure	Ungrammatical example sentence
Subject-verb-object sentence (24)	Le fidèle prennent les hiboux. DET.SG.MASC believer take-3PL DET.PL owl-PL The believer *are taking the owls.
Subject relative (12)	Voici le tricheur qui craignent les arbitres. Here.is DET.SG.MASC cheater REL.SUB fear-3PL DET.PL referee-PL This is the cheater that *are afraid of the referees.
Complement clause (12)	Les serveurs disent que le saumon cuisent. DET.PL waiter-PL say-3PL COMP DET.SG.MASC salmon cook-3PL The waiters say that the salmon *are cooking.
Common object relative (72)	Voici les gorilles que la touriste décrivent. Here.are DET.PL gorilla-PL REL.OBJ DET.SG.FEM tourist describe-3PL These are the gorillas that the tourist *are describing.
Transposed object relative (24)	Voici le pervers que proscrit les juristes. Here.is DET.SG.MASC deviant REL.OBJ condemn-3SG DET.PL jurist-PL This is the deviant that the jurists *condemns.

The stimuli were created by replacing György et al. (2024)’s pseudowords with French nouns.

Pseudo-words used in György et al.’ (2024) study were replaced with nouns, controlling for gender (152 masculine and 136 feminine) and number of syllables to ensure that the present materials are maximally comparable. As such, all nouns used in this study were disyllabic (mirroring pseudo-words from the Jabberwocky study), also keeping the number of syllables relatively constant between grammatical and ungrammatical sentences. Based on findings of animacy effects on syntax processing in Villata et al. (2018), animacy was controlled to the extent to which it was possible given all other constraints. To minimize any potential animacy effect, the majority of the materials (103 out of 144 sentences) an animate subject and an animate object. In sentences where this was not possible due to the verb used, the order of preference was: inanimate subject – inanimate object (37 sentences), inanimate subject – animate object (1 sentence), animate subject – inanimate object (3 sentences).

Sentences were recorded with natural prosody by a female native French speaker. Noise reduction and normalization were carried out in version 3.0.0 of Audacity(R) recording and editing software (Audacity Team, 2021).

***Priming procedure***: The priming procedure used in this study was identical to György et al. (2024)’s mixed design experiment (Experiment 2). Six sentences were presented auditorily after each prime. One miniblock contained one of the three possible 32-s prime conditions followed by 3 grammatical and 3 ungrammatical sentences in a randomized order. The relative ratio of different syntactic structures in each mini block remained identical throughout the experiment. 3 mini blocks of the three different prime conditions were presented in an alternating order, which was the same for the entire experiment in an experimental list. Six experimental lists were constructed and distributed evenly across participants based on the order of presentation of the prime conditions (8 x (Regular-Silence-Irregular), 8 x (Regular-Irregular-Silence), 8 x (Irregular-Regular-Silence), 8 x (Irregular-Silence-Regular), 8 x (Silence-Irregular-Regular), 8 x (Silence-Regular-Irregular)). Participants were instructed to press a response key to indicate whether the sentence they heard was grammatical (S) or not (K), and do so as quickly and accurately as possible. The entire experiment was run online. This task was built and administered using Psychopy (Peirce et al., 2019) and administered on Pavlovia (Peirce et al., 2019; Bridges et al., 2020).

Following previous priming studies (Przybylski et al., 2013; György et al., 2024), the dependent variable analyzed was Discrimination sensitivity (d’). This is a measure based on the proportion of hits (correct responses for ungrammatical sentences), false alarms (incorrect responses for grammatical sentences), correct rejections (correct responses for grammatical sentences) and misses (incorrect responses for ungrammatical sentences). D′ was calculated as z(p[hits]) – z(p[FAs]), where p[hits] is the proportion of hits and p[FAs] is the proportion of false alarms. Scores of 1 were changed to 0.99 and scores of 0 to 0.01 in an attempt to avoid infinite values, (Stanislaw and Todorov, 1999). An index of sensitivity to the regularity of the prime (d’_Regular_-d’_Irregular_) was also created for the analyses of relationships between tasks.

##### Profile of music perception skills (PROMS)

To estimate participants’ skills in discriminating rhythmic and accent structure, the rhythm and beat (accent) subtests of the short version of the Profile of Music Perception Skills (PROMS, Law and Zentner, 2012; Zentner and Strauss, 2017) were used. Participants had to decide, on a 5-point Likert scale ranging from ‘Definitely Same’ to ‘Definitely different’, whether a third sequence they heard was identical or different to the two identical accent or rhythmic sequences. A total of 8 rhythm and 10 beat trials were presented following the standardized short PROMS test. This task was built and administered online using LimeSurvey (LimeSurvey Project Team/Schmitz, 2015).

Composite scores of the PROMS rhythm subtest and the PROMS beat subtest were calculated based on the PROMS user guide. A PROMS combined score was obtained by adding up the two composite scores in accordance with the official PROMS guide.

##### Spontaneous speech synchronization (SSS) task

The spontaneous speech synchronization task used in this study was adapted for French from Assaneo et al. (2019). In this task, participants heard two 80-s randomized syllable sequences at roughly 4.5 syllables per second. Under the impression that they were performing a syllable discrimination task, participants were presented with four syllables and asked to indicate (Yes or No) whether or not those were part of the syllable sequence they heard. To make their task more challenging, participants were asked to continuously whisper the syllable “tah” while they were listening to the syllable sequences and were informed that their voice would be recorded to ensure they were whispering continuously. The real measure of interest was whether participants would spontaneously synchronize their whisper rate to the presentation rate of the syllable sequence. This task was built and administered online using Labvanced (Finger et al., 2016).

Following Assaneo et al. (2019), a phase-locking value (PLV) between the 4.5 Hz presentation rate and participants’ whisper rate was calculated as the dependent variable. This continuous variable represents the extent to which participants’ whisper rate was comparable to the syllable presentation rate.

#### General procedure

Participants completed the experiment from their own homes. They were asked to wear headphones and use a computer with the most recent version of Google Chrome installed. All participants completed the experimental tasks in the following order: Grammaticality judgment, PROMS and SSS.

#### Data analyses

Grammaticality judgment data (d’) was analyzed using Linear Mixed Effects Regression Models in R (RC Team, 2018), using the lme4 package (Bates et al., 2015). Main effects and interactions are reported from the anova(model) function, while pairwise comparisons were realized using the emmeans package.

All models contained Participant as a random effect. Following György et al. (2024)’s study, an initial model with Prime (Regular vs. Silence vs. Irregular) as the only fixed effect was run on d’, with follow-up models including Miniblockhalf (sentences 1–3 vs. 4–6 after each prime) as an additional fixed effect.

Accuracy was assessed using Generalized Linear Mixed Effects Regression Models in R (RC Team, 2018), using the lme4 package (Bates et al., 2015). Main effects and interactions are reported from relevant minimal pair model comparisons [anova (model1, model2)], while pairwise comparisons were realized using the emmeans package.

All accuracy models included Participant and Item as random effects, while the fixed effects were Prime (Regular vs. Silence vs. Irregular) and Grammaticality of the sentence (Grammatical vs. Ungrammatical).

Relationships between the different tasks were evaluated by simple Spearman correlation analyses using the Hmisc package in R.

### Results

#### RPE in the grammaticality judgment task

All items below chance performance (0.5 accuracy) were removed from further analysis. As performance in these sentences was low, and participants reported that these sentence structures were very unnatural in spoken French, all object relative sentences with stylistic inversion were also removed from the analysis. Consequently, a total of 117 out of 144 sentences per participant were analyzed. 1 participant below 0.5 accuracy was removed.

Grammaticality judgment performance was generally high (*M* = 0.895, SD = 0.307) and its distribution right-skewed. Correct response rates were generally higher for grammatical (*M* = 0.923, SD = 0.266) than for ungrammatical trials (*M* = 0.866, SD = 0.341), as is often the case in a grammaticality judgment task.

**Figure 1:**
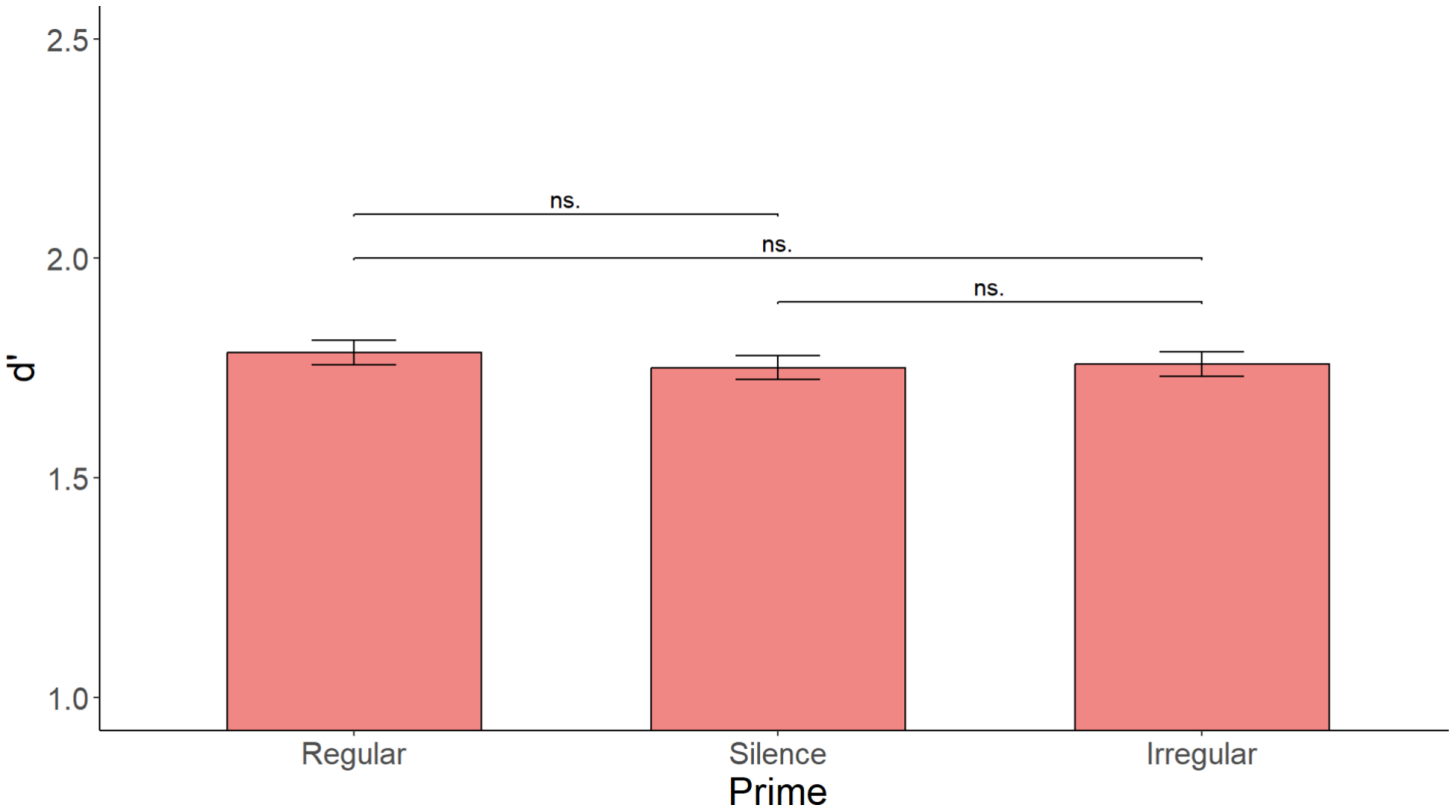
Mean d’ by prime over all sentences in Experiment 1. No significant effect of prime.

As illustrated in Figure 1, the initial model with Prime as the only fixed effect showed no significant main effect of Prime [*F*(2, 216) = 0.531, *p* = 0.599]. Supplementary Table 1 shows the summary of the fixed effects of the model, while Supplementary Table 2 reports main effects and interactions obtained using the anova(model) function in R.

**Figure 2:**
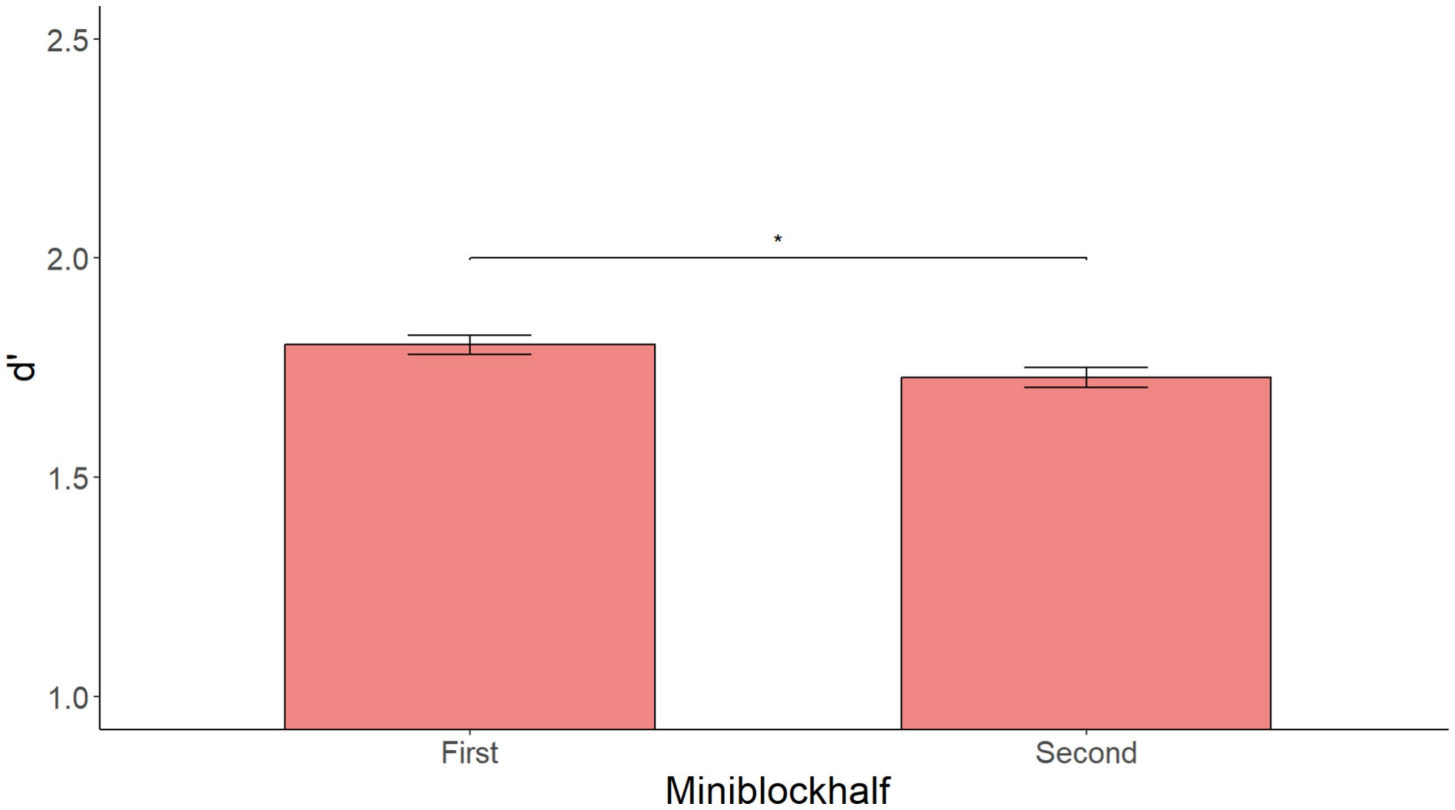
Mean d’ by Miniblockhalf (sentences 1–3 vs. 4–6 after each prime) in Experiment 1. Significantly higher performance in sentences 1–3 than 4–6.

A model with Prime, Miniblockhalf, and their interaction yielded a significant main effect of Miniblockhalf [*F*(1, 540) = 4.906, *p* = 0.027] showing higher accuracy in the first three than in the last three sentences but no other significant effects. This effect is visualized in Figure 2. Supplementary Table 3 shows the summary of the fixed effects of the model, while Supplementary Table 4 shows main effects and interactions obtained using the anova(model) function in R.

**Figure 3:**
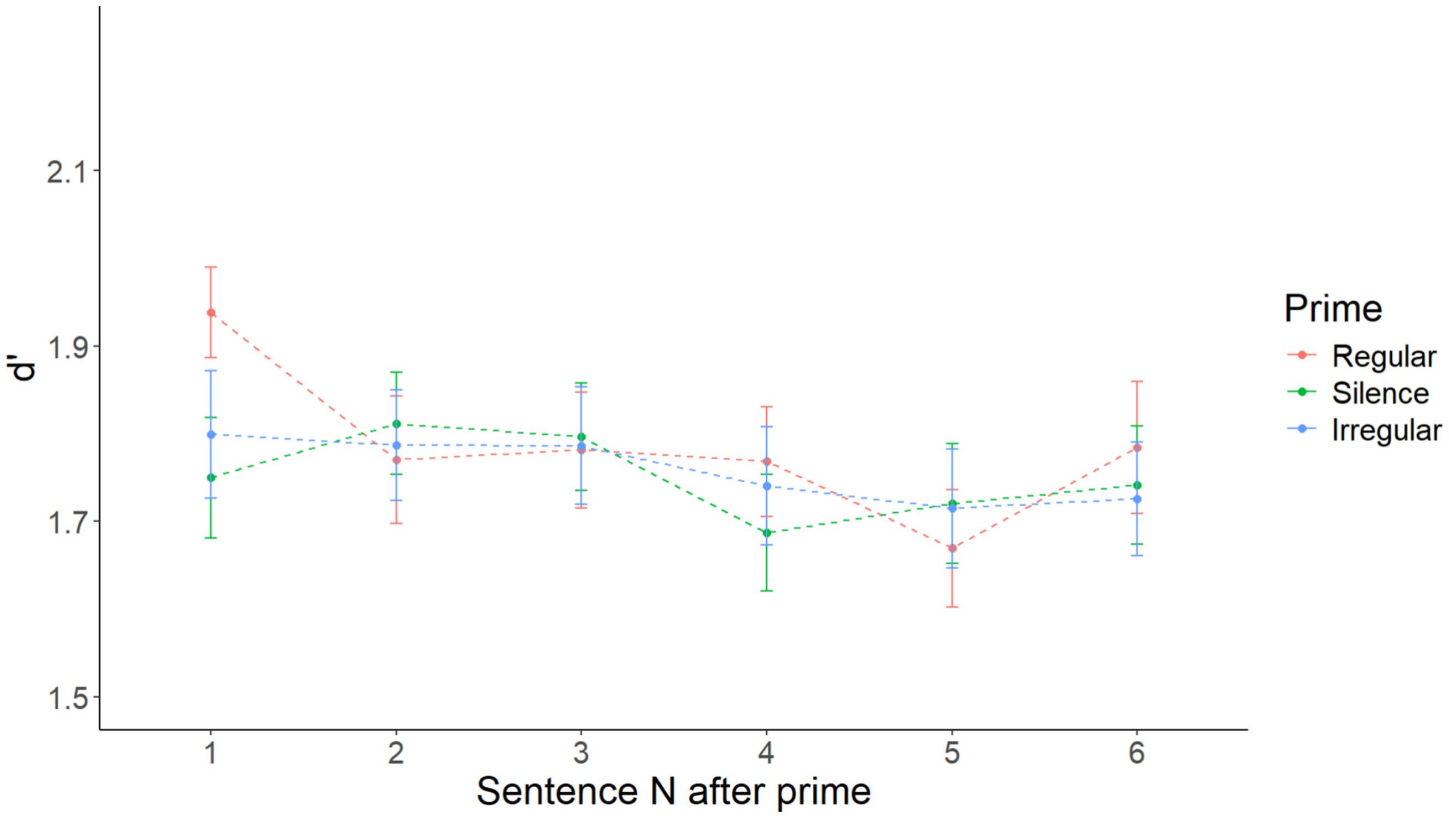
Mean d’ by prime over sentences 1–6, respectively, in Experiment 1.

**Figure 4:**
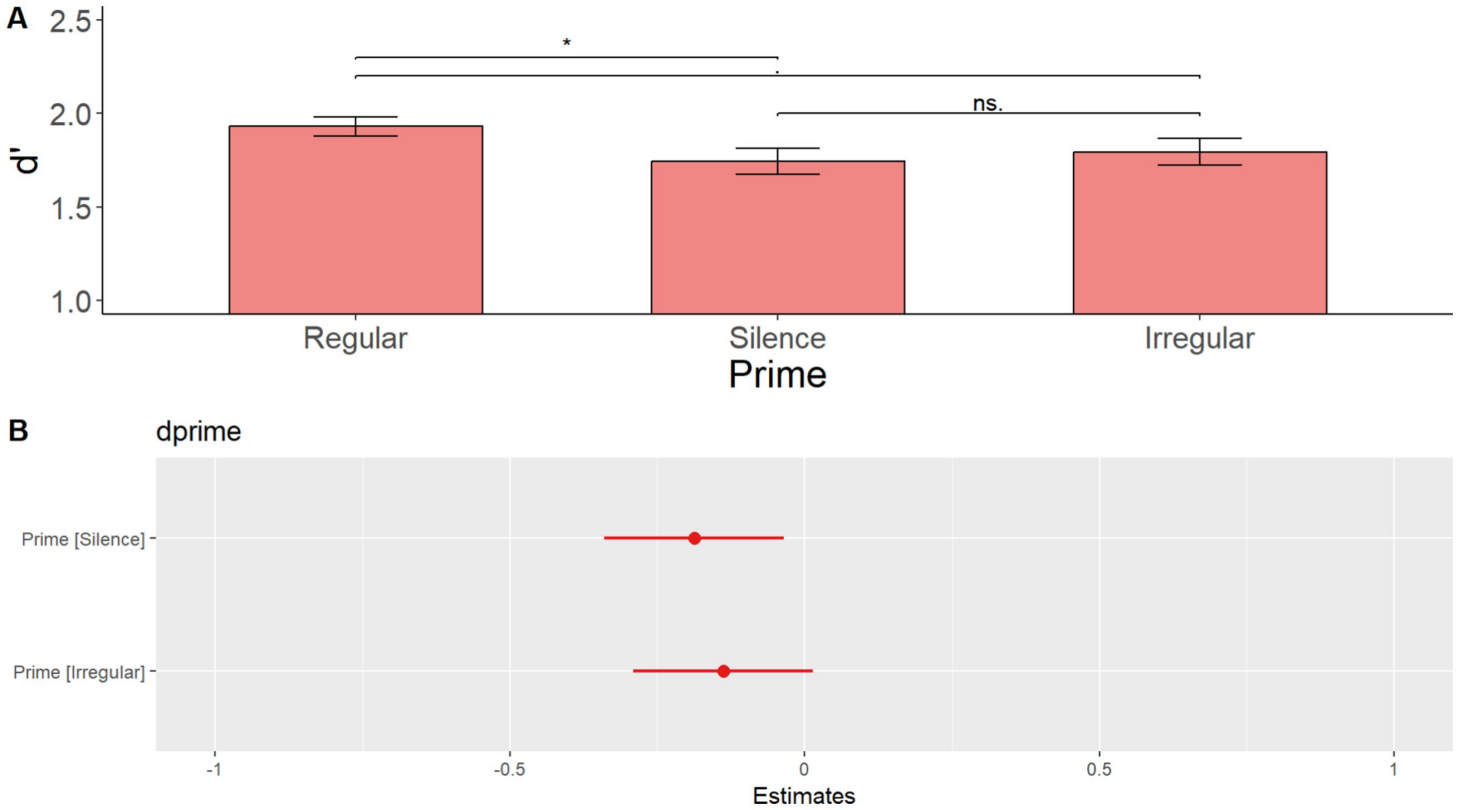
**(A)** Mean d’ by prime in the first sentence after each prime in Experiment 1. Grammaticality judgment d’ is significantly higher after a regular prime than after silence, and marginally higher than after irregular. **(B)** Plotted estimates of the model d’ ~ Prime + 1|Subject run on sentence 1 after each prime in Experiment 1.

After visual inspection of the data illustrated in Figure 3, it appeared that there might be a short-lived priming effect, restricted to only sentence 1 after the prime. Accordingly, a model with Prime as the only main effect on sentence 1 after priming only yielded a significant main effect of Prime [*F*(2, 216) = 3.128, *p* = 0.046]. Specifically, pairwise comparisons with the Tukey correction showed that d’ after a regular prime was higher than after silence (*t* = 2.412 *p* = 0.044), while the difference between the regular and irregular (*t* = 1.779, *p* = 0.179) and the irregular and silence (*t* = −0.633, *p* = 0.802) conditions was not significant. D′ by prime in the first sentence after each prime is shown in Figure 4. Supplementary Table 5 shows the summary of the fixed effects of the model, while Supplementary Table 6 reports main effects and interactions obtained using the anova(model) function in R.

#### Profile of music perception skills

Eleven participants did not complete PROMS due to a software error. PROMS rhythm (*W* = 0.977, *p* = 0.058), 0beat (*W* = 0.985, *p* = 0.283), and combined (*W* = 0.959, *p* = 0.518) scores were normally distributed. No outliers were more than 2.5 SD below the mean and no extreme outliers were observed, so the entirety of the dataset was kept for analysis. Significant positive correlations were observed between PROMS rhythm and PROMS beat [*r*(106) = 0.45, *p* < 0.001], as well as between the combined PROMS score and both sub-tests [beat: *r*(106) = 0.86, *p* < 0.001, rhythm: *r*(106) = 0.83, *p* = 0.001].

#### Spontaneous speech synchronization task

**Figure 5:**
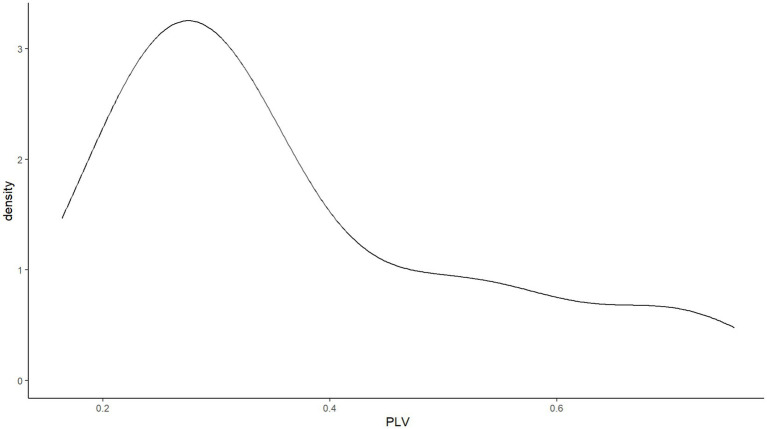
No bimodal distribution of SSS phase-locking values in Experiment 1.

Sixty participants’ (51.3%) audio recordings had to be dropped for one of the following reasons: inaudible whispering, environmental noise, or audible experimental stimuli. This drop rate is high, but is comparable to that reported by Assaneo et al. (2019). The remaining data points showed a skewed non-normal distribution (*W* = 0.860, *p* < 0.001). As shown in Figure 5, while this distribution is skewed, it is not a bimodal distribution like the one observed in English speakers by Assaneo et al. (2019). The sample of the present study contained more low-synchronizers than high-synchronizers, only 17 out of 57 participants exhibiting phase-locking values above 0.4.

#### Correlations

Grammaticality judgment d’ showed a significant positive correlation with PROMS beat [*r*(99) = 0.202, *p* = 0.045], and combined [*r*(99) = 0.203, *p* = 0.044], but not PROMS rhythm [*r*(99) = 0.130, *p* = 0.199]. No significant correlations were observed between sensitivity to regularity (in 1 or 6 sentences) and any of the PROMS measures.

Overall grammaticality judgment d’ also showed a negative correlation with SSS phase-locking values such that the more participants tended to synchronize their production rate to the 4.5 Hz presentation, the lower their grammaticality judgment d’ was [*r*(54) = −0.316, *p* = 0.020]. SSS PLVs showed no correlations with sensitivity to rhythmic priming of any of the PROMS measures.

## Discussion

Experiment 1 sought to test the hypothesis that lexico-semantic information is involved in the structure building mechanisms by which rhythmic priming is realized. To do this, we created natural language minimal pairs of jabberwocky sentences for which a short-term rhythmic priming effect (3 sentences rather than 6 traditionally reported in the priming literature) was reported. No rhythmic priming effect was observed for 6 or 3 sentences following the prime. Instead, the results showed an even shorter priming effect restricted to the first sentence following rhythmic stimulation. The priming effect manifested in a significant difference between the rhythmically regular and the silent baseline condition, while the difference between the rhythmically regular and irregular conditions was only marginal and irregular did not differ from silence. This suggests a weak but beneficial effect from exposure to a regular rhythm compared to baseline (rather than a penalizing effect from exposure to the irregular rhythm). The present results also replicate the positive relationship reported between beat discrimination and grammaticality judgment performance. As for spontaneous speech synchronization performance, we found a negative correlation between participants’ tendency to synchronize their speech output to an external rhythm. In other words, the higher tendency a participant exhibited to synchronize their speech output, the worse they performed in the GJ task, independently of priming. Interestingly, we do not replicate the bimodal distribution reported in English-speaking SSS participants (Assaneo et al., 2019; Kern et al., 2021). This data point will be addressed in greater detail in the General Discussion.

The present study differs from György et al. (2024)’s mixed design experiment in two factors. First, the current Experiment 1 used fully natural language stimuli to test our initial hypothesis. While the present results are contradictory to our initial prediction, they need not necessarily contradict the hypothesis behind said prediction. If rhythmic priming is realized through linguistics structure-building processes involving lexico-semantic information, it is possible that the availability of this information itself facilitates structure building in a fully developed language processing system, thus reducing any potential influence from extra-linguistic structural information such as rhythmic priming. If this is the case, we would expect a weaker priming effect in natural language stimuli than their jabberwocky minimal pairs.

Second, the present experiment had to be run online. As such, the experimental environment was far less controlled and more prone to potential distractions than that in a sound-attenuated testing booth. Indeed, while a large number of visual psychology experiments have been reported to be easily replicable online, auditory experiments have been more prone to lag (Anwyl-Irvine et al., 2020; Bridges et al., 2020). It is plausible that potential distractions and differences in participants’ auditory environment may have reduced the rhythmic priming effect that is already short-lived in the laboratory. Interestingly, testing online did not seem to result in a noticeable reduction in overall grammaticality judgment performance (*M* = 0.906, SD = 0.292 in György et al.’s Experiment 2, *M* = 0.895, SD = 0.307 here), rhythm and beat discrimination ability (results here), or the positive relationship between rhythm discrimination and grammaticality judgment. Nevertheless, the change in modality of testing may have introduced enough noise to interfere with the relatively subtle effect of rhythmic priming while leaving more robust effects intact.

To explore these two potential explanations, Experiment 2 tested rhythmic priming on jabberwocky materials online (replicating György et al.’s Experiment 2 online), while Experiment 3 replicated the present Experiment 1 in a laboratory setting. Experiment 3 also included the offline auditory oddball task used in György et al.’ (2024) study. Experiment 2 and 3 were otherwise identical to Experiment 1.

## Experiment 2

### Methods

#### Participants

89 native French-speaking typical adults (67 women) participated in the experiment. Participants were between 18 and 43 years of age (*M* = 22.30, SD = 4.76), with no history of neurological disorders, specific language impairment, amusia or psychiatric issues.

Participants gave informed consent prior to the start of the experiment, were not made aware of the purpose of the study, and were compensated with either course credits or 20 CHF for their time. The experiment was approved by the University of Geneva Research Ethics Committee (PSE.20191004.04).

#### Materials and procedure

##### Grammaticality judgment (GJ) task

The entire experiment was run online. This task was built and administered using Psychopy (Peirce & al., 2019) and administered on Pavlovia (Bridges et al., 2020). Otherwise, this task was identical to the grammaticality judgment task in György et al. (submitted)’s Experiment 2 (mixed design).

*The Profile of Music Perception Skills (PROMS), Spontaneous speech synchronization (SSS) task*, *general procedure* and *data analyses* were identical to those described in Experiment 1 of the present study.

### Results

#### RPE in grammaticality judgment task

All items below chance performance (0.5 accuracy) were removed from further analysis. Similar to Experiment 1, because of low performance and qualitative feedback from participants, all object relative sentences with stylistic inversion were also removed from analysis. Consequently, a total of 119 out of 144 sentences per participant were preserved. No participants were removed due to below chance performance.

Grammaticality judgment performance was generally high (*M* = 0.848, SD = 0.360) and its distribution right-skewed. Correct response rates were generally higher for grammatical (*M* = 0.896, SD = 0.306) than for ungrammatical trials (*M* = 0.799, SD = 0.401).

**Figure 6:**
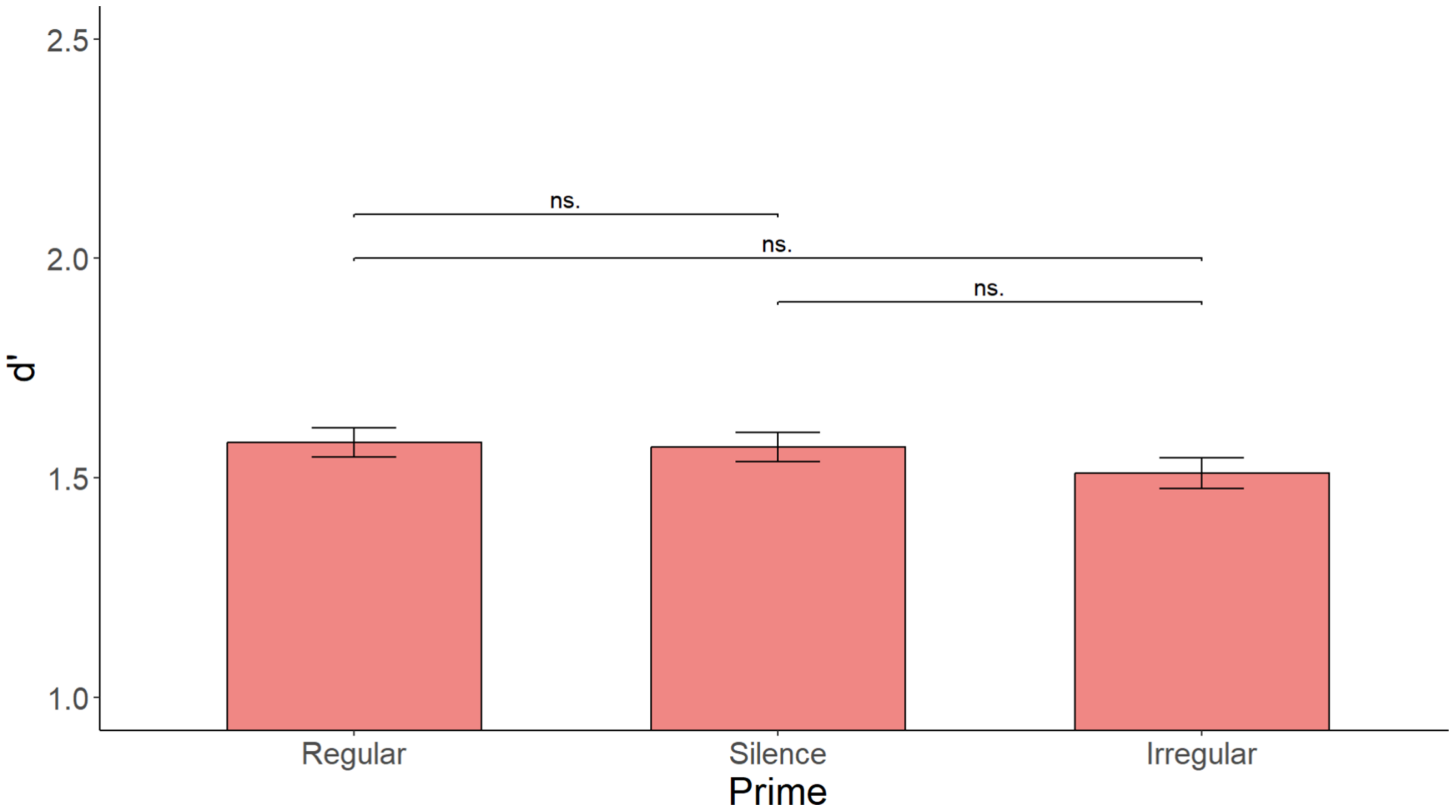
Mean d’ by prime over all sentences in Experiment 2. No significant effect of prime.

The initial model with Prime as the only fixed effect yielded no significant main effect of Prime [*F*(2, 176) = 0.527 *p* = 0.592]. Supplementary Table 7 shows the summary of the fixed effects of the model, Supplementary Table 8 the main effect of prime, while Figure 6 shows mean d’ by Prime.

No significant effects were shown in the output of the next model with Prime, Miniblockhalf and their interaction. Supplementary Table 9 shows the summary of the fixed effects of the model, while Supplementary Table 10 shows main effects and interactions obtained using the anova(model) function in R.

**Figure 7:**
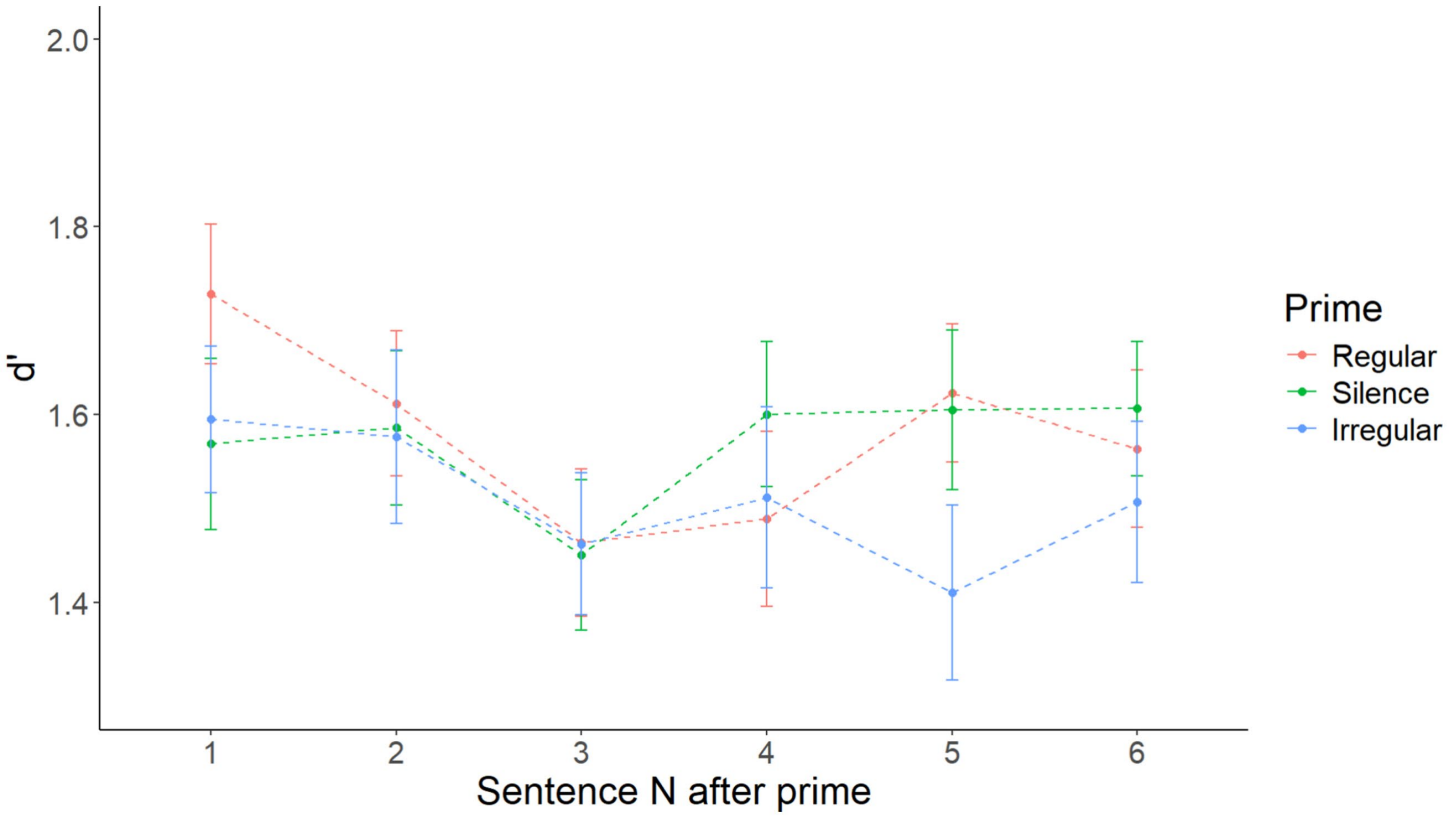
Mean d’ by prime over sentences 1–6, respectively, in Experiment 2.

**Figure 8:**
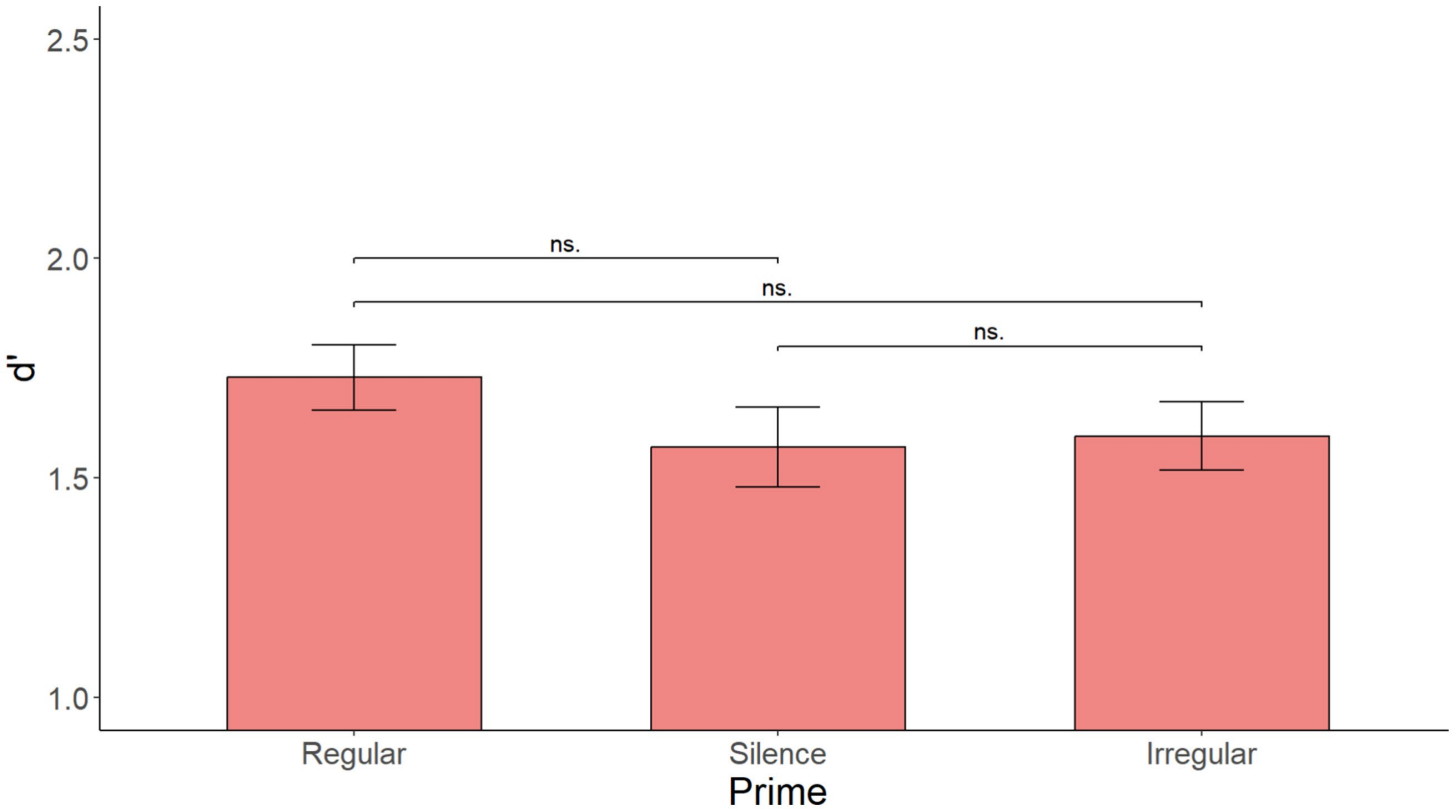
Mean d’ by prime in the first sentence after each prime in Experiment 2. No significant effect of prime.

**Figure 9:**
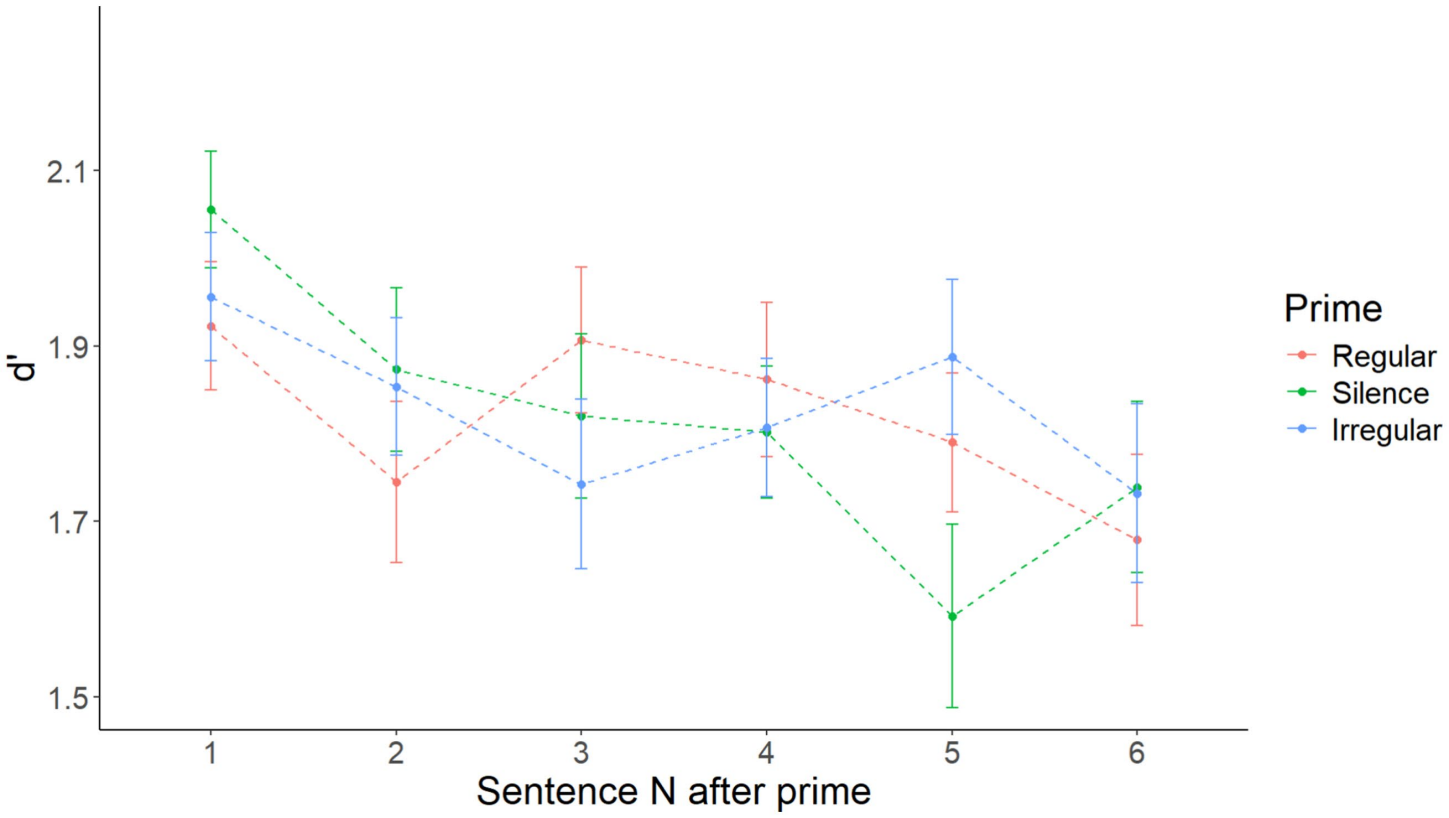
Mean d’ by prime over sentences 1–6, respectively, in Experiment 3.

**Figure 10:**
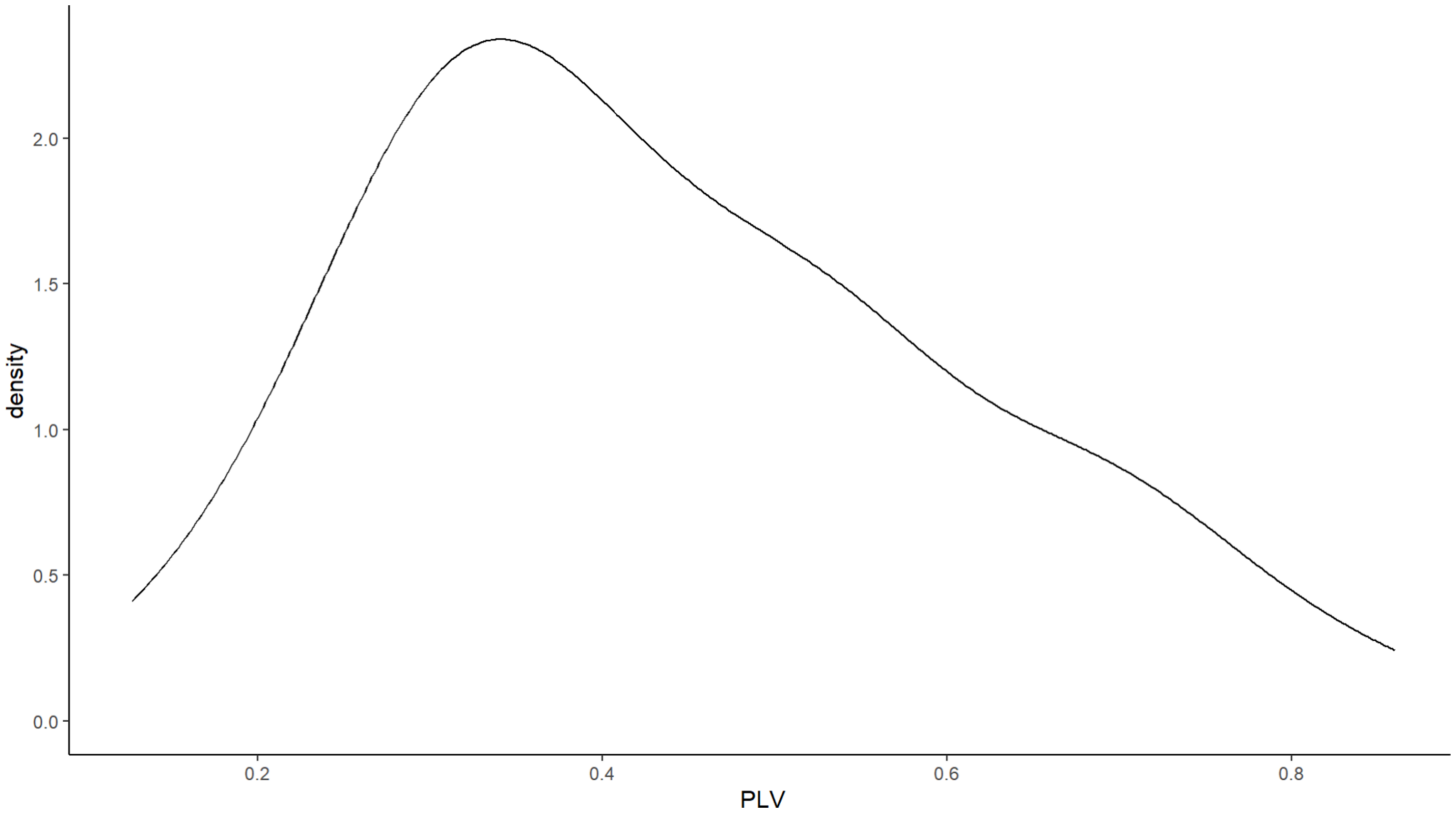
No bimodal distribution of SSS phase-locking values in Experiment 3.

As in Experiment 1, based on visual inspection of the data in Figure 7, it appeared that there might be a priming effect restricted to sentence 1 after the prime. However, as shown in the Figure 8, the main of Prime was not significant [*F*(2, 176) = 1.667, *p* = 0.192]. The model summary and the main effect of Prime are shown in Figures 9, 10, respectively.

#### Profile of music perception skills

Five participants could not complete PROMS due to a technical issue. PROMS rhythm composite scores showed a non-normal distribution (*W* = 0.959, *p* = 0.009), while PROMS beat (*W* = 0.982, *p* = 0.282) and combined (*W* = 0.973, *p* = 0.070) were normally distributed. No PROMS beat outliers were more than 2.5SD below the mean, so no participants were removed from analysis. As in the previous experiment, significant positive correlations were observed between PROMS rhythm and PROMS beat [*r*(84) = 0.53, *p* < 0.001], as well as between the combined PROMS score and both sub-tests [beat: *r*(84) = 0.88, *p* < 0.001, rhythm: *r*(84) = 0.86, *p* < 0.001].

#### Spontaneous speech synchronization task

**Figure 11:**
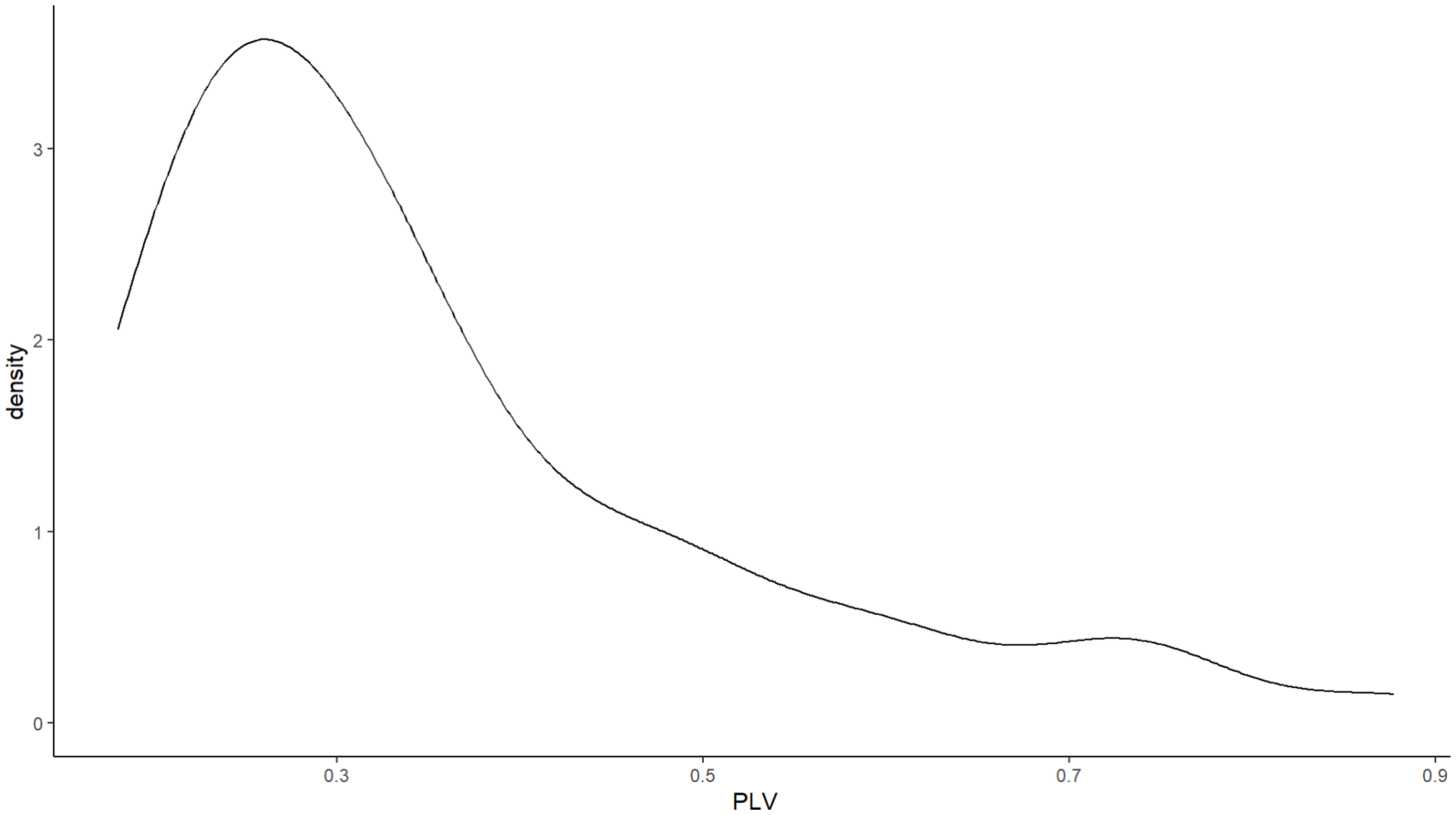
No bimodal distribution of SSS phase-locking values in Experiment 2.

Forty-five participants’ (50.6%) audio recordings had to be dropped due to inaudible whispering, environmental noise, or audible experimental stimuli. The remaining data points showed a skewed non-normal distribution (*W* = 0.845, *p* < 0.001). Shown in Figure 11 and similar to Experiment 1, this distribution was skewed but not bimodal. 15 out of 44 participants showed phase-locking values above 0.4.

#### Correlations

Grammaticality judgment d’ showed a significant positive correlation with PROMS rhythm [*r*(80) = 0.259, *p* = 0.021] and combined [*r*(80) = 0.233, *p* = 0.037], but not PROMS beat [*r*(80) = 0.148, *p* = 0.189].

A positive correlation was observed between sensitivity to the regularity of the prime and PROMS beat [*r*(80) = 0.659, *p* = 0.016] and combined [*r*(80) = 0.242, *p* = 0.031], but not rhythm [*r*(80) = 0.137, *p* = 0.225]: the better participants performed in beat discrimination, the more they benefited from a regular compared to an irregular prime.

No further correlations were found between performance on the SSS task, PROMS and grammaticality judgment.

### Discussion

Experiment 2 was run as a pure minimal pair of Experiment 1 using Jabberwocky materials from György et al. (2024). Results showed (1) no priming effect on 6, 3, or 1 sentence, (2) a replication of the positive relationship between rhythm discrimination and grammaticality judgment, (3) a positive correlation between beat discrimination and sensitivity to the regularity of the prime, (4) no bimodal distribution in spontaneous speech synchronization, and (5) no relationships between spontaneous speech synchronization and other measures. Experiment 2 replicated the positive relationship between rhythm discrimination and grammaticality judgment performance found by György et al. (2024) and Experiment 1 of the present study. Along with similar data points in the literature, these data point toward shared resources between rhythm and language processing.

Similar to Experiment 1, the present experiment found no bimodal distribution in spontaneous speech synchronization in native French-speaking typical adults. Instead, the data showed that a large portion of the sample is unlikely to synchronize their whisper rate to an external presentation rate without explicit instruction. These results replicate those presented in Experiment 1 and differ from well-replicated results in native English speakers, suggesting that native French speakers and native English speakers may differ in their tendency to spontaneously synchronize. Alternatively, it is possible that similar to the case of the RPE, the less controlled environment of online testing may have interfered with synchronization results. However, data from English speakers showed remarkably similar synchronization distributions measured online and offline, making the modality of testing an unlikely account for the results observed in these experiments. Nevertheless, Experiment 3 ran the SSS task in a controlled laboratory setting to rule out modality of testing as a potential confound.

The most puzzling result of the present experiment was the non-replication of the RPE reported in 3 sentences in Jabberwocky in the laboratory and in 1 sentence in natural language online. These results appear to be in line with our initial prediction suggesting a more pronounced RPE in natural language than in jabberwocky, assuming that online testing reduces the rhythmic priming effect altogether compared to a laboratory setting. Taken together, Experiments 1 and 2 show a priming effect in natural language but not in Jabberwocky, supporting the hypothesis put forward by György et al. (2024), according to which rhythmic priming may be realized through structure building processes involving lexico-semantic information. However, both of these experiments showed a much shorter-lived priming effect compared to those reported in the literature in children with typical and atypical development or typical adults using different linguistic stimuli and in typical adults processing the same Jabberwocky material used in the present study. Therefore, to systematically evaluate whether online testing globally reduces the RPE in natural language and jabberwocky materials, Experiment 3 used the same protocol as Experiment 1 in a laboratory setting.

As Experiment 3 was run in the lab, we decided to include an offline auditory oddball task to measure selective auditory attention (Schwartze et al., 2013; György et al., 2024) in an attempt to measure the role of auditory attention in the RPE. We expected to observe a correlation between sensitivity to the rhythmic regularity of the prime and oddball performance under the hypothesis that rhythmic priming is influenced by auditory attention.

## Experiment 3

### Methods

#### Participants

60 native French-speaking typical adults (43 women) participated in the experiment. Participants were between 18 and 44 years of age (*M* = 23.15, SD = 5.18), reporting no history of neurological disorders, specific language impairment, amusia, or psychiatric issues.

Participants gave informed consent prior to the start of the experiment, were not made aware of the purpose of the study, and were compensated with either course credits or 20 CHF for their time. The experiment was approved by the University of Geneva Research Ethics Committee (PSE.20191004.04).

#### Materials and procedure

All tasks of Experiment 3 were administered in a sound-attenuated experimental booth. Otherwise, they were identical to the task described in Experiment 1 of the present study.

##### Profile of music perception skills (PROMS)

This task was identical to the one used in György et al. (2024) and in Experiments 1 and 2 of the present study.

##### Spontaneous speech synchronization (SSS) task

This task was identical to the one used in Experiments 1 and 2 of the present study.

##### Auditory oddball

Following György et al. (2024), auditory attention was measured in an oddball paradigm in which participants heard 512 standard (600 Hz) and 128 deviant (660 Hz) tones, and were asked to report the number of deviant tones they heard (67). This task was built and administered using Presentation® software (Neurobehavioral Systems, Inc, 2024).

The dependent variable was precision calculated as the absolute value of the difference between the participant’s response and 128.

#### General procedure

Participants were seated in a sound-isolated booth where they performed the experiment on a computer and heard the auditory stimuli through headphones. All participants completed the experimental tasks in the following order: Grammaticality judgment, Auditory Oddball, SSS and PROMS.

### Results

#### RPE in grammaticality judgment task

All items below chance performance (0.5 accuracy) were removed from analysis. Similar to Experiments 1 and 2, because of low participant performance and qualitative feedback, all object relative sentences with stylistic inversion were also removed from analysis. Consequently, a total of 119 out of 144 sentences per participant were preserved. No additional subjects were removed.

Grammaticality judgment performance was generally high (*M* = 0.905, SD = 0.293) and its distribution right-skewed. Correct response rates were higher for grammatical (*M* = 0.930, SD = 0.256) than for ungrammatical trials (*M* = 0.880, SD = 0.325).

**Figure 12:**
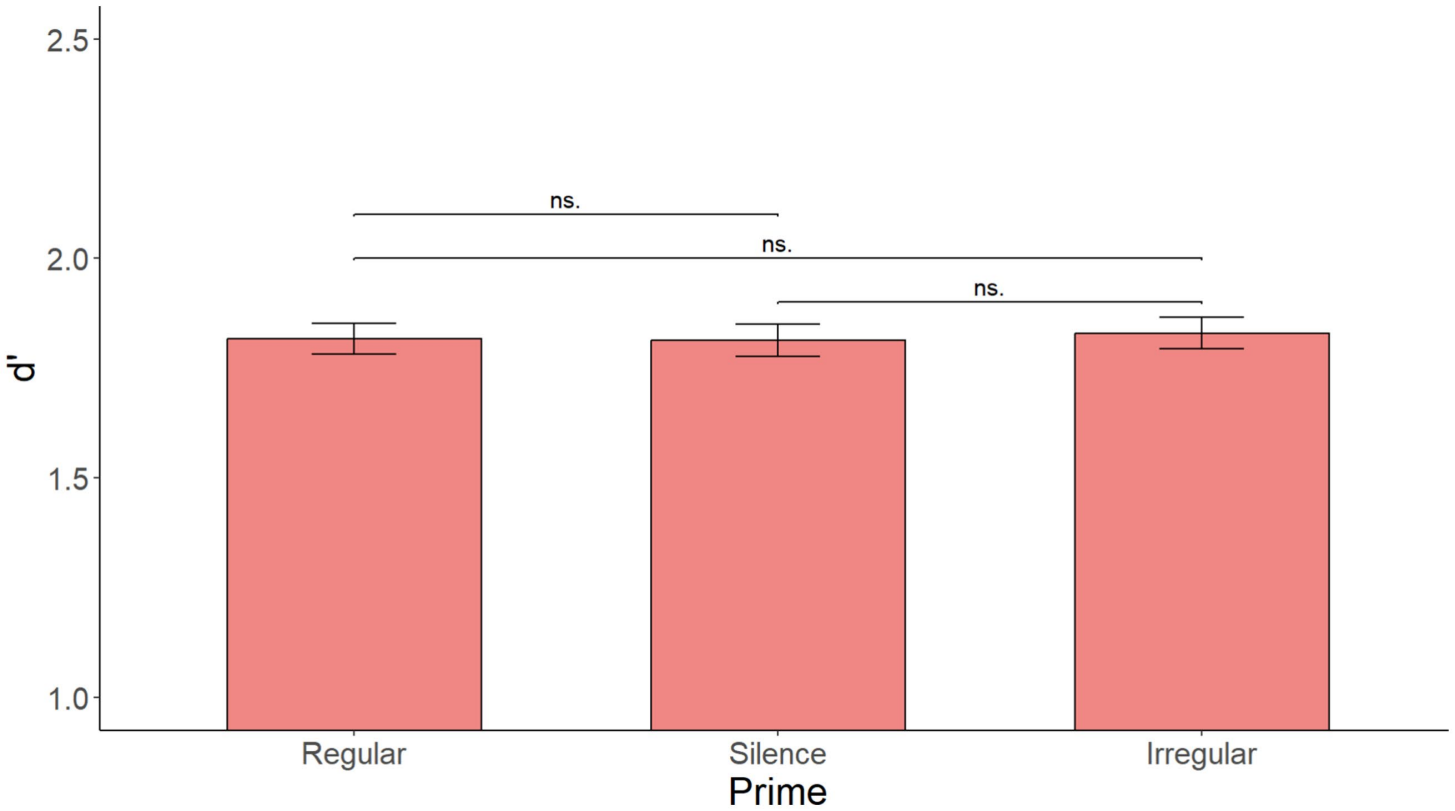
Mean d’ by prime over all sentences in Experiment 3. No significant effect of prime.

The linear mixed effects regression model with Prime as the only fixed effect yielded no significant main effect of Prime [*F*(2, 118) = 0.240, *p* = 0.787]. Supplementary Tables 13, 14 show the summary of the fixed effects of the model as well as the main effect of Prime, while Figure 12 shows mean d’ by Prime. Visual inspection of the data in Figure 9 showed no indication that the priming effect would be more present in the first sentence than in the rest of the sentences.

#### Profile of music perception skills

PROMS composite scores were normally distributed with no outliers (beat: *W* = 0.964, *p* = 0.076 rhythm: *W* = 0.971, *p* = 0.170, combined: *W* = 0.986, *p* = 0.746). Significant positive correlations were observed between PROMS rhythm and PROMS beat [*r*(60) = 0.53, *p* < 0.001], as well as between the combined PROMS score and both sub-tests [beat: *r*(60) = 0.88, *p* < 0.001 rhythm: *r*(60) = 0.86, *p* < 0.001].

#### Auditory oddball

In the Auditory Oddball task, three participants with a precision score of over 30 (an extremely high difference between the participant’s response and the correct number of deviant tones) were removed from analysis. Additionally, four further participants whose precision scores were over 2 standard deviations above the grand mean were also excluded from the final dataset (*M* = 6.038, SD = 4.682).

#### Spontaneous speech synchronization task

13 participants’ (21.7%) audio recordings had to be dropped for one of the following reasons: inaudible whispering, environmental noise, or audible experimental stimuli. Unlike in Experiments 1 and 2, the remaining data points showed a normal distribution (*W* = 0.965, *p* = 0.167). Just like in Experiments 1 and 2, Figure 10 shows no bimodal distribution. 25 out of 47 participants showed phase-locking values above 0.4.

#### Correlations

Grammaticality judgment d’ showed no relationship with PROMS beat [*r*(60) = −0.005, *p* = 0.968], PROMS rhythm [*r*(60) = 0.065, *p* = 0.619], or combined [*r*(60) = 0.042, *p* = 0.752].

A positive correlation was found between sensitivity to the regularity of the prime and PROMS rhythm, and a marginal relationship with PROMS combined, but not beat: the better participants performed in rhythm discrimination, the more they benefited from a regular compared to an irregular prime [rhythm: *r*(60) = 0.298, *p* = 0.021, combined: *r*(60) = 0.246, *p* = 0.059, beat: *r*(80) = 0.119, *p* = 0.364].

Oddball scores showed no significant correlations with overall grammaticality judgment d’ [*r*(53) = 0.012, *p* = 0.936] or sensitivity to regularity [*r*(53) = −0.125, *p* = 0.383].

A significant positive correlation was observed between SSS phase-locking values and overall grammaticality judgment d’: the more participants tended to synchronize their whisper rate to the syllable presentation rate, the higher their GJ performance [*r*(47) = 0.360, *p* = 0.013].

No further significant correlations were found between grammaticality judgment, PROMS, the SSS and oddball tasks.

## General discussion

The present study sought to explore two hypotheses. According to Hypothesis 1, a shared cognitive system responsible for coding abstract hierarchical structure is recruited by both language and musical rhythm processing (Fitch and Martins, 2014; Martins et al., 2017; Heard and Lee, 2020; Asano et al., 2021; György et al., 2024). Hypothesis 2, proposed by György et al. (2024), attempted to explain the short-term rhythmic priming effect they reported in French-speaking typical adults processing Jabberwocky sentences. The authors hypothesized that lexico-semantic information might play a role in the linguistic structure building processes that recruit this overlapping network. We tested predictions of these hypotheses in three experiments that varied in the naturalness (Natural language vs. Jabberwocky) of the linguistic stimuli used, and testing modality (Online vs. In lab).

Together with the in-lab jabberwocky data (György et al., 2024), the main results of the present study showed that (1) the RPE in typical adults is a short-lasting effect found in only three of five experiments of the two studies, (2) the RPE is probably susceptible to influence from multiple factors, but its effect size (or lack thereof) is not explained by Naturalness or Modality of testing alone (see Supplementary Table 15 for a summary of priming effects using the current stimuli), (3) rhythm and beat discrimination and grammaticality judgment performance show a weak but replicable (in 2 out of 3 experiments) positive relationship, (4) French speakers do not show a bimodal distribution in spontaneous speech synchronization. Overall, data from the rhythmic priming experiment appear to be inconclusive: the greatly reduced effect in Experiment 1 and null effects in Experiments 2 and 3 do not conclusively corroborate our hypotheses. However, under a frequentist statistical approach used in the present study, the lack of an effect does not allow us to conclusively reject the hypotheses, either. Instead, replicable correlations between rhythm discrimination and grammaticality judgment performance provide arguments in favor of Hypothesis 1, even though alternative explanations for these results are also possible. We address each of these in detail in the following sections.

### Weak priming effect in typical adults

Data from all experiments of the present study [and both experiments reported by György et al. (2024) and one experiment reported by Canette et al. (2020a)] suggested that the rhythmic priming effect in typical adults is greatly reduced compared to children with typical or atypical development. While each of our experiments were constructed as a minimal pair equivalent of Experiment 2 of György et al. (2024), differing in Naturalness, Modality of testing, or both, only one out of three experiments yielded a significant effect of rhythmic priming on syntactic processing. Furthermore, even this effect observed in Experiment 1 (Natural language, Online) was restricted to the first sentence presented after each prime, i.e., even shorter than that reported by György et al. (2024) in jabberwocky (in-lab). Neither the online jabberwocky nor the in-lab natural language study yielded a significant rhythmic priming effect.

Crucially, the reduced duration of the observed RPE does not seem to be fully accounted for by Naturalness of the linguistic stimuli or Modality of testing alone. In the lab, no priming effect was observed in natural language, compared to a three-sentence effect reported by György et al. (2024). Online testing showed an inverse trend: no effect in jabberwocky and an effect on the first sentence in natural language. Taken together, these findings suggest that the reduction of lexico-semantic information by using Jabberwocky is not responsible for the short or no priming effect reported in this study and in György et al. (2024). This is in line with results from agreement attraction suggesting that the syntactic structure of jabberwocky and natural language sentences like those used in the current study are processed similarly. This finding also suggests that lexico-semantic information (or the lack thereof) does not modulate the RPE.

As acknowledged in György et al. (2024), it is possible that highly repetitive subject-verb number agreement errors (as opposed to a combination of gender assignment, subject-verb number agreement and subject-verb person agreement used by several studies) led to higher grammaticality judgment performance in typical adults. This proposal is in line with results showing a significant priming effect over 6 sentences in typical adults processing sentences with more varied morphosyntactic errors but no behavioral priming effect when using simple agreement errors. At least two counter-arguments could be made to this proposal. Firstly, the analyses reported by Canette et al. (2019) (separate *t*-tests by items and by participants) would require a greater number of participants than 25 typical adults to reach 0.8 statistical power according to G*Power (Faul et al., 2007). Secondly, and potentially more importantly, one recent study reports no RPE in English-speaking children with typical development (Kim et al., 2024). This data point seems to suggest that the RPE is less robust in typically developing populations than believed based on previous reports (Canette et al., 2019; György et al., 2024) and may be specific to age or language (however, priming effects in psychology have often proven difficult to replicate, Cesario, 2014).

The recent series of short- and null effects of rhythmic priming on typical adults and children may also suggest that the RPE itself is a relatively weak effect in typical populations (György et al., 2024; Kim et al., 2024). If this is the case, the relatively stable RPE in atypical populations may stem primarily from a compensation effect. In this case, an impaired temporal or structural processing system can be reactivated by the regularity of an external rhythmic stimulus, greatly enhancing immediately subsequent language processing (Kotz et al., 2005; Przybylski et al., 2013). Comorbidity of temporal and language processing has been reported in developmental language disorder, developmental dyslexia, as well as neurodegenerative disorders and focal lesions to the basal ganglia (Corriveau and Goswami, 2009; Flaugnacco et al., 2014; Kotz and Schmidt-Kassow, 2015). These populations also appear to benefit from external rhythmic stimulation to improve their language processing (Flaugnacco et al., 2014; Kotz and Schmidt-Kassow, 2015; Przybylski et al., 2013), which may rely on compensatory mechanisms to boost improved processing. These compensation effects could be realized through subcortico-cortical networks such as the cerebellum-thalamus-preSMA, similar to accounts explaining how patients with focal lesions or neurodegenerative disorders to the basal ganglia can take advantage of external regularities using their intact cerebellar network (Kotz et al., 2009, 2014; Kotz and Schwartze, 2010; Kotz and Schmidt-Kassow, 2015; Schwartze and Kotz, 2016). However, a robust typical(ly developing) system would not benefit much from such compensation effects, or may even be insensitive to input from non-specialized pathways that might otherwise actively support syntactic structure building. In a hierarchical cognitive control-based account of the RPE, a typical system could process syntactic structures without the need for a supporting metrical grid, and may have sufficient resources to process regular and irregular metrical structures as well as subsequent syntactic structures optimally. Conversely, an atypical system may not only benefit from the presence of an easy-to-extract hierarchical structure actively supporting syntactic structure building, but might also have fewer available hierarchical cognitive control resources to process syntactic structures immediately after encountering an irregular rhythmic prime, the structure of which is not apparent enough for highly automatic processes to extract (Asano et al., 2021).

As far as the musical stimuli are concerned, while the primes developed by Przybylski et al. (2013) have repeatedly been shown to generate a priming effect in typical and atypical children, it is also possible that the structural difference between the regular and the irregular prime is simply not enough to influence a fully developed structure processing system. Using primes whose musical rhythmic structures differ in clear and precise points may help bring further insight to this issue. Recent studies with children and adults have chosen to use more musical, more varied, and multi-layered primes, creating irregular primes by randomizing the order of acoustic events in each layer (Canette et al., 2019; Canette et al., 2020a). Introducing precise stepwise changes between the regular and irregular primes used in these experiments may help uncover what aspect(s) or musical rhythmic regularity contribute to the rhythmic priming effect.

One major point of criticism that the present work has to acknowledge concerns the use of the grammaticality judgment task for evaluating the effect of rhythmic priming on syntactic processing. Crucially, this task has been proposed to rely heavily on metalinguistic skills rather than being a pure measure of syntactic processing (Ladányi et al., 2020; Serratrice et al., 2009; Sprouse, 2007). It therefore seems critical to determine whether the RPE lies in fundamental mechanisms of syntactic computation and not in the meta-cognitive component of the task, and to gather evidence about how these effects manifest on-line during sentence processing. So far, one rhythmic priming study has looked at the P600, reporting a higher P600 amplitude after exposure to a regular than after an irregular rhythmic prime (Canette et al., 2020b). This finding was interpreted as indicating a beneficial effect of rhythmic priming on syntactic processing. One major advantage of using ERPs is that they constitute an on-line direct measure of language processing with an impressive temporal resolution, while a potential disadvantage lies in its precise interpretation. While the cited RPE paper interprets an increase in P600 amplitude as facilitation of syntactic processing, studies investigating superimposed regular speech meter on German sentences often interpret a decreased P600 as facilitated syntactic integration (Roncaglia-denissen et al., 2013). Alternatively, future research could also use off-line tasks that probe into more ecological components of language processing. One such task could involve Who did what to whom? types of comprehension questions that evaluate the thematic roles of a given sentence. Indeed, in contrast to the common assumption that typical adult parsing is flawless, the few studies that explored the actual parses built by healthy adults have shown an unexpectedly high rate of comprehension errors, suggesting that such tasks may probe language processing in a more targeted manner than grammaticality judgment (Villata and Franck, 2020). Furthermore, thematic role comprehension questions also constitute one of the very few behavioral language comprehension measures sensitive to regularizing speech rhythm (Roncaglia-denissen et al., 2013), a sensitivity that may extend to rhythmic priming. It appears that a combination of online and offline methods will provide more complete and more ecological answers to questions probing the overlap between rhythm and language processing than grammaticality judgments alone.

### Individual differences: a promising avenue?

The most important finding from the French version of the Spontaneous Speech Synchronization task is the lack of a bimodal distribution reported in English speakers (Assaneo and Poeppel, 2018; Assaneo et al., 2019, 2020; Lizcano-Cortés et al., 2022). In other words, while their sample of English speakers showed that some participants are prone to spontaneously entrain their speech output to the rhythmicity of the input while others do not, the current sample of French speakers seem to be globally low synchronizers. While performance was slightly higher when the task was administered in the lab, a strikingly low number of phase-locking values at or above 0.6 (the 25th percentile of high synchronizers in English speakers, Assaneo et al., 2019) were observed in all three experiments. The lack of a bimodal distribution in the present study appeared to be independent of the modality of testing (online vs. in lab). Not only does this lend credence to these results, it is also consistent with Assaneo and colleagues’ original results in English speakers, showing the same (there, bimodal) distribution online as that observed in a laboratory setting. So far, the bimodal distribution in SSS phase-locking values has been reported in speakers of English and German, both stress-timed languages. It is plausible that speakers of syllable-timed languages such as French show a globally different tendency to synchronize their speech output to an external presentation rate by virtue of the rhythmicity of their own language. Indeed, language background appears to impact Normalized Pairwise Variability Index in classical music (Patel, 2008), sensitivity to music (Patel and Daniele, 2003, but see recent critique by Condit-Schultz, 2019), and French speakers in particular tend to perform poorly in beat or stress processing tasks (Dupoux et al., 1997, 2008; Schmidt-Kassow et al., 2011). Indeed, French in particular lacks or has very weak lexical stress, with French speakers at least sometimes being unable to distinguish contrastive stress patterns (Dupoux et al., 1997, 2008). While the lack of lexical stress in everyday speech rhythm does not impact language processing in native speakers, French participants may not instinctively impose subjective rhythmization on the (French) syllables they were exposed to in the SSS task. This lack of imposed rhythmic structure may have, in turn, made it less obvious for these participants to instinctively synchronize their production rate to the external stimulus. More work is needed to identify whether this difference in the tendency (not) to synchronize speech output to an external rhythm stem from speaking a syllable-timed language or another property specific to French.

Given the lack of bimodal distribution in the SSS task performance, it is not surprising that we found no clear advantage of high synchronizers over low synchronizers in grammatically judgment. The more puzzling part of the lack of the systematic relationship appears to stem from a negative correlation between SSS and grammaticality judgment in Experiment 1 and a positive correlation in Experiment 3. This is particularly interesting as mean phase-locking values in the in-lab experiment were slightly higher than in Experiments 1 and 2. Therefore, it appears that better synchronizers draw more of a positive (or null) correlation with grammaticality judgment performance, while worse synchronizers show more of a negative (or null) relationship. This pattern also emerged after a median split in Experiments 1 (below median: *r*(27) = −0.403, *p* = 0.037, at or above median: *r*(27) = 0.10, *p* = 0.959), 2 (below median: *r*(27) = 0.094, *p* = 0.641, at or above median: *r*(27) = 0.209, *p* = 0.296) and 3 (below median: *r*(23) = −0.162, *p* = 0.460, at or above median: *r*(24) = 0.170, *p* = 0.427).

As for PROMS, Experiments 1 and 2 (but not 3) of the present study replicated the finding that grammaticality judgment accuracy correlates positively with rhythm and beat discrimination in typical adult participants. György et al. (2024) reported that this relationship remained stable even after controlling for selective auditory attention, suggesting that it is likely to be somewhat specific to music and language processing.

This data point replicates not only similar observations in typical adults and provides evidence for an overlap between rhythm and syntax processing, it also adds to similar to observations in the developmental literature (Gordon et al., 2014, 2015; Lee et al., 2020; Persici et al., 2023) and evidence showing that adults with more refined rhythmic skills are better able to perceive sentences, but not words, in noise (Slater and Kraus, 2015; Yates et al., 2019). One possible explanation for this result may lie in a shared system responsible for hierarchical structure processing given that PROMS, the grammaticality judgment task used in the present study and its direct predecessor, sentence-in-noise (but not word-in-noise) processing, as well as the rhythmic discrimination, sentence comprehension and elicited morphosyntax production tasks used in the developmental literature require the internal construction of correct structural (metrical or syntactic) representations. Alternatively, it is also possible that the rhythm-syntax link is mediated through another aspect of language processing such as prosody, which involves both fine auditory processing and the construction of hierarchical structures relevant but not equivalent to syntactic structures. As mentioned before, findings showing relationships between rhythm and syntax (but not phonology) processing in children (Gordon et al., 2014, 2015) and reports of adults with better rhythmic skills showing better sentence-in-noise, but not words-in-noise performance (Slater and Kraus, 2015; Yates et al., 2019) suggest that the correlations observed in this study are likely to be subserved by some form of structural processing in the two domains. However, we must acknowledge that the present study did not use a specific control task (other than the SSS, where the real task is disguised from participants) to rule out a link due to general task engagement. Therefore, while we believe that simple task engagement is unlikely to account for these results, future studies should include additional tasks that serve to measure task engagement outside of structure building processes. While more research is still needed to gain a precise understanding of the overlap between rhythm and syntax processing, and suggest that identifying rhythmic abilities that can act as markers of language performance may be a worthwhile pursuit.

The present study found a stronger priming effect the better our participants performed at beat (Experiment 2) or rhythm (Experiment 3) discrimination in Experiments 2 and 3 (but not 1). This pattern is not consistent with the negative correlation between PROMS scores and the RPE reported by György et al. (2024).

Given the recent reports of short-term or null rhythmic priming effects in adults (György et al., 2024 and the present study) and in children (Kim et al., 2024), it appears that the RPE in typical populations may be a less robust effect than that reported in children with developmental language disorder or developmental dyslexia. Consequently, future research could benefit from identifying individual psychometric differences making participants more or less susceptible to the RPE. Indeed, the RPE was linked to rhythm and discrimination, auditory selective attention, as well as the frequency of listening to music, tendency to tap to a rhythm, and seeing music as a social bond (Canette et al., 2019; György et al., 2024). A more refined comprehension of psychometric factors modulating the RPE would not only enrich our theoretical understanding of the cognitive and neural mechanisms underlying rhythmic priming, but would also enrich our knowledge of the rhythm-language overlap.

## Data Availability

The raw data supporting the conclusions of this article will be made available by the authors, without undue reservation.

## References

[ref1] Anwyl-Irvine A. Dalmaijer E. S. Hodges N. Evershed J. K. (2020). Realistic precision and accuracy of online experiment platforms, web browsers, and devices. Behav. Res. Methods 53, 1407–1425. doi: 10.3758/s13428-020-01501-5, PMID: 33140376 PMC8367876

[ref2] Asano R. Boeckx C. Seifert U. (2021). Hierarchical control as a shared neurocognitive mechanism for language and music. Cognition 216:104847. doi: 10.1016/j.cognition.2021.104847 34311153

[ref3] Assaneo M. F. Poeppel D. (2018). The coupling between auditory and motor cortices is rate-restricted: evidence for an intrinsic speech-motor rhythm. Sci. Adv. 4, 1–10. doi: 10.1126/sciadv.aao3842 PMC5810610 29441362

[ref4] Assaneo M. F. Rimmele J. M. Sanz Perl Y. Poeppel D. (2020). Speaking rhythmically can shape hearing. Nat. Hum. Behav. 5, 71–82. doi: 10.1038/s41562-020-00962-0 33046860

[ref5] Assaneo M. F. Ripollés P. Orpella J. Lin W. M. de Diego-Balaguer R. Poeppel D. (2019). Spontaneous synchronization to speech reveals neural mechanisms facilitating language learning. Nat. Neurosci. 22, 627–632. doi: 10.1038/s41593-019-0353-z 30833700 PMC6435400

[ref6] Audacity Team (2021). Audacity(R): free audio editor and recorder [computer application] (version 3.0.0). Audacity® software is copyright © 1999-2021 audacity Team. It is free software distributed under the terms of the GNU general public. Available online at: https://audacityteam.org/

[ref7] Ayotte J. Peretz I. Hyde K. (2002). Congenital amusia. Brain 125, 238–251. doi: 10.1093/brain/awf028 11844725

[ref8] Bates D. Maechler M. Bolker B. Walker S. Christensen R. H. B. Singmann H. (2015). Package “lme4”: Linear mixed-effects models using ‘Eigen’ and S4. Available online at: https://cran.r-project.org/web/packages/lme4/index.html

[ref9] Bedoin N. Besombes A. M. Escande E. Dumont A. Lalitte P. Tillmann B. (2018). Boosting syntax training with temporally regular musical primes in children with cochlear implants. Ann. Phys. Rehabil. Med. 61, 365–371. doi: 10.1016/j.rehab.2017.03.004 28506442

[ref10] Bedoin N. Brisseau L. Molinier P. Roch D. Tillmann B. (2016). Temporally regular musical primes facilitate subsequent syntax processing in children with specific language impairment. Front. Neurosci. 10, 1–11. doi: 10.3389/fnins.2016.00245 27378833 PMC4913515

[ref11] Bock K. Miller C. A. (1991). Broken agreement. Cogn. Psychol. 23, 45–93. doi: 10.1016/0010-0285(91)90003-7 2001615

[ref12] Bridges D. Pitiot A. MacAskill M. R. Peirce J. W. (2020). The timing mega-study: comparing a range of experiment generators, both lab-based and online. PeerJ 8, 1–29. doi: 10.7717/peerj.9414 PMC7512138 33005482

[ref13] Canette L.-H. Bedoin N. Lalitte P. Bigand E. Tillmann B. (2019). The regularity of rhythmic primes influences syntax processing in adults. Audit. Percept. Cogn. 2, 163–179. doi: 10.1080/25742442.2020.1752080

[ref14] Canette L. H. Fiveash A. Krzonowski J. Corneyllie A. Lalitte P. Thompson D.. (2020a). Regular rhythmic primes boost P600 in grammatical error processing in dyslexic adults and matched controls. Neuropsychologia 138:107324. doi: 10.1016/j.neuropsychologia.2019.107324 31877312

[ref15] Canette L. H. Lalitte P. Bedoin N. Pineau M. Bigand E. Tillmann B. (2020b). Rhythmic and textural musical sequences differently influence syntax and semantic processing in children. J. Exp. Child Psychol. 191:104711. doi: 10.1016/j.jecp.2019.104711 31770684

[ref16] Cason N. Astésano C. Schön D. (2015a). Bridging music and speech rhythm: rhythmic priming and audio-motor training affect speech perception. Acta Psychol. 155, 43–50. doi: 10.1016/j.actpsy.2014.12.002 25553343

[ref17] Cason N. Hidalgo C. Isoard F. Roman S. Schön D. (2015b). Rhythmic priming enhances speech production abilities: evidence from prelingually deaf children. Neuropsychology 29, 102–107. doi: 10.1037/neu0000115 25068663

[ref18] Cason N. Schön D. (2012). Rhythmic priming enhances the phonological processing of speech. Neuropsychologia 50, 2652–2658. doi: 10.1016/j.neuropsychologia.2012.07.018 22828660

[ref19] Cesario J. (2014). Priming, replication, and the hardest science. Perspect. Psychol. Sci. 9, 40–48. doi: 10.1177/1745691613513470 26173239

[ref20] Chern A. Tillmann B. Vaughan C. Gordon R. L. (2018). New evidence of a rhythmic priming effect that enhances grammaticality judgments in children. J. Exp. Child Psychol. 173, 371–379. doi: 10.1016/j.jecp.2018.04.007 29778278 PMC5986615

[ref21] Condit-Schultz N. (2019). Deconstructing the nPVI. Music. Percept. 36, 300–313. doi: 10.1525/mp.2019.36.3.300

[ref22] Corriveau K. H. Goswami U. (2009). Rhythmic motor entrainment in children with speech and language impairments: tapping to the beat. Cortex 45, 119–130. doi: 10.1016/j.cortex.2007.09.008 19046744

[ref23] Criscuolo A. Schwartze M. Prado L. Ayala Y. Merchant H. Kotz S. A. (2023). Macaque monkeys and humans sample temporal regularities in the acoustic environment. Prog. Neurobiol. 229:102502. doi: 10.1016/j.pneurobio.2023.102502 37442410

[ref24] Ding N. Melloni L. Zhang H. Tian X. Poeppel D. (2015). Cortical tracking of hierarchical linguistic structures in connected speech. Nat. Neurosci. 19, 158–164. doi: 10.1038/nn.4186 26642090 PMC4809195

[ref25] Dupoux E. Pallier C. Sebastian N. Mehler J. (1997). A Destressing “Deafness” in French? J. Mem. Lang. 36, 406–421. doi: 10.1006/jmla.1996.2500

[ref26] Dupoux E. Sebastián-Gallés N. Navarrete E. Peperkamp S. (2008). Persistent stress ‘deafness’: the case of French learners of Spanish. Cognition 106, 682–706. doi: 10.1016/j.cognition.2007.04.001 17592731

[ref27] Faul F. Erdfelder E. Lang A.-G. Buchner A. (2007). G*power 3: a flexible statistical power analysis program for the social, behavioral, and biomedical sciences. Behav. Res. Methods 39, 175–191. doi: 10.3758/bf03193146 17695343

[ref28] Finger H. Goeke C. Diekamp D. Standvoß K. König P. (2016). LabVanced: a unified JavaScript framework for online studies. 2017 International conference on computational social science IC2S2 July 10–13, 2016, Cologne, Germany.

[ref29] Fitch W. T. Martins M. D. (2014). Hierarchical processing in music, language, and action: Lashley revisited. Ann. N. Y. Acad. Sci. 1316, 87–104. doi: 10.1111/nyas.12406 24697242 PMC4285949

[ref30] Fiveash A. Bedoin N. Gordon R. L. Tillmann B. (2021). Processing rhythm in speech and music: shared mechanisms and implications for developmental speech and language disorders. Neuropsychology 35, 771–791. doi: 10.1037/neu0000766 34435803 PMC8595576

[ref31] Fiveash A. Bedoin N. Lalitte P. Tillmann B. (2020). Rhythmic priming of grammaticality judgments in children: duration matters. J. Exp. Child Psychol. 197:104885. doi: 10.1016/j.jecp.2020.104885 32559634

[ref32] Flaugnacco E. Lopez L. Terribili C. Zoia S. Buda S. Tilli S.. (2014). Rhythm perception and production predict reading abilities in developmental dyslexia. Front. Hum. Neurosci. 8, 1–14. doi: 10.3389/fnhum.2014.00392 24926248 PMC4045153

[ref33] Franck J. Colonna S. Rizzi L. (2015). Task-dependency and structure-dependency in number interference effects in sentence comprehension. Front. Psychol. 6:349. doi: 10.3389/fpsyg.2015.00349 25914652 PMC4392591

[ref34] Franck J. Lassi G. Frauenfelder U. H. Rizzi L. (2006). Agreement and movement: a syntactic analysis of attraction. Cognition 101, 173–216. doi: 10.1016/j.cognition.2005.10.003 16360139

[ref35] Franck J. Soare G. Frauenfelder U. H. Rizzi L. (2010). Object interference in subject-verb agreement: the role of intermediate traces of movement. J. Mem. Lang. 62, 166–182. doi: 10.1016/j.jml.2009.11.001

[ref36] Franck J. Wagers M. (2020). Hierarchical structure and memory retrieval mechanisms in agreement attraction. PLoS One 15:e0232163. doi: 10.1371/journal.pone.0232163 32428038 PMC7237028

[ref37] Fujii S. Wan C. Y. (2014). The role of rhythm in speech and language rehabilitation: the SEP hypothesis. Front. Hum. Neurosci. 8, 1–15. doi: 10.3389/fnhum.2014.00777 25352796 PMC4195275

[ref38] Glushko A. Poeppel D. Steinhauer K. (2022). Overt and implicit prosody contribute to neurophysiological responses previously attributed to grammatical processing. Sci. Rep. 12:14759. doi: 10.1038/s41598-022-18162-3 36042220 PMC9427746

[ref39] Gordon R. L. Jacobs M. S. Schuele C. M. Mcauley J. D. (2015). Perspectives on the rhythm-grammar link and its implications for typical and atypical language development. Ann. N. Y. Acad. Sci. 1337, 16–25. doi: 10.1111/nyas.12683 25773612 PMC4794983

[ref40] Gordon R. L. Shivers C. M. Wieland E. A. Kotz S. A. Yoder P. J. Devin McAuley J. (2014). Musical rhythm discrimination explains individual differences in grammar skills in children. Dev. Sci. 18, 635–644. doi: 10.1111/desc.12230 25195623

[ref41] György D. Saddy J. D. Kotz S. A. Franck J. (2024). Rhythmic priming of syntactic processing in jabberwocky: a short-lived effect. Lang Cogn Neurosci 39, 939–958. doi: 10.1080/23273798.2024.2361737

[ref42] Heard M. Lee Y. S. (2020). Shared neural resources of rhythm and syntax: an ALE meta-analysis. Neuropsychologia 137:107284. doi: 10.1016/j.neuropsychologia.2019.107284 31783081

[ref43] Kaufeld G. Bosker H. R. ten Oever S. Alday P. M. Meyer A. S. Martin A. E. (2020). Linguistic structure and meaning organize neural oscillations into a content-specific hierarchy. J. Neurosci. 40, 9467–9475. doi: 10.1523/jneurosci.0302-20.2020 33097640 PMC7724143

[ref44] Kern P. Assaneo M. F. Endres D. Poeppel D. Rimmele J. M. (2021). Preferred auditory temporal processing regimes and auditory-motor synchronization. Psychonomic Bulletin Rev. 28, 1860–1873. doi: 10.3758/s13423-021-01933-w PMC8642338 34100222

[ref45] Kim H.-W. McLaren K. E. Lee Y. S. (2024). No influence of regular rhythmic priming on grammaticality judgment and sentence comprehension in English-speaking children. J. Exp. Child Psychol. 237:105760. doi: 10.1016/j.jecp.2023.105760 37647840

[ref46] Kotz S. A. Gunter T. C. Wonneberger S. (2005). The basal ganglia are receptive to rhythmic compensation during auditory syntactic processing: ERP patient data. Brain Lang. 95, 70–71. doi: 10.1016/j.bandl.2005.07.039

[ref47] Kotz S. A. Schmidt-Kassow M. (2015). Basal ganglia contribution to rule expectancy and temporal predictability in speech. Cortex 68, 48–60. doi: 10.1016/j.cortex.2015.02.021 25863903

[ref48] Kotz S. A. Schwartze M. (2010). Cortical speech processing unplugged: a timely subcortico-cortical framework. Trends Cogn. Sci. 14, 392–399. doi: 10.1016/j.tics.2010.06.005 20655802

[ref49] Kotz S. A. Schwartze M. Schmidt-Kassow M. (2009). Non-motor basal ganglia functions: a review and proposal for a model of sensory predictability in auditory language perception. Cortex 45, 982–990. doi: 10.1016/j.cortex.2009.02.010 19361785

[ref50] Kotz S. A. Stockert A. Schwartze M. (2014). Cerebellum, temporal predictability and the updating of a mental model. Philos. Transac. Royal Soc. B 369:403. doi: 10.1098/rstb.2013.0403 PMC4240970 25385781

[ref51] Ladányi E. Lukács Á. Gervain J. (2021). Does rhythmic priming improve grammatical processing in Hungarian-speaking children with and without developmental language disorder? Dev. Sci. 1–12:13112. doi: 10.1111/desc.13112 PMC8530934 34060171

[ref52] Ladányi E. Persici V. Fiveash A. Tillmann B. Gordon R. L. (2020). Is atypical rhythm a risk factor for developmental speech and language disorders? Wiley Interdiscip. Rev. Cogn. Sci. 11, 1–32. doi: 10.1002/wcs.1528 PMC7415602 32244259

[ref53] Large E. W. Herrera J. A. Velasco M. J. (2015). Neural networks for beat perception in musical rhythm. Front. Syst. Neurosci. 9, 1–14. doi: 10.3389/fnsys.2015.00159 26635549 PMC4658578

[ref54] Law L. N. C. Zentner M. (2012). Assessing musical abilities objectively: Construction and validation of the profile of music perception skills. PLoS One 7:e52508. doi: 10.1371/journal.pone.0052508 23285071 PMC3532219

[ref55] Lee Y. S. Ahn S. Holt R. F. Schellenberg E. G. (2020). Rhythm and syntax processing in school-age children. Dev. Psychol. 56, 1632–1641. doi: 10.1037/dev0000969 32700950

[ref56] LimeSurvey Project Team/Schmitz (2015). LimeSurvey: an open source survey tool (2.00). Hamburg: Limesurvey Project.

[ref57] Lizcano-Cortés F. Gómez-Varela I. Mares C. Wallisch P. Orpella J. Poeppel D.. (2022). Speech-to-speech synchronization protocol to classify human participants as high or low auditory-motor synchronizers. STAR Protoc 3:101248. doi: 10.1016/j.xpro.2022.101248 35310080 PMC8931471

[ref58] Martin A. E. Doumas L. A. A. (2017). A mechanism for the cortical computation of hierarchical linguistic structure. PLoS Biol. 15:e2000663. doi: 10.1371/journal.pbio.2000663 28253256 PMC5333798

[ref59] Martins M. J. D. Fischmeister F. P. S. Gingras B. Bianco R. Puig-Waldmueller E. Villringer A.. (2020). Recursive music elucidates neural mechanisms supporting the generation and detection of melodic hierarchies. Brain Struct. Funct. 225, 1997–2015. doi: 10.1007/s00429-020-02105-7 32591927 PMC7473971

[ref60] Martins M. D. Gingras B. Puig-Waldmueller E. Fitch W. T. (2017). Cognitive representation of “musical fractals”: processing hierarchy and recursion in the auditory domain. Cognition 161, 31–45. doi: 10.1016/j.cognition.2017.01.001 28103526 PMC5348576

[ref61] Nayak S. Coleman P. L. Ladányi E. Nitin R. Gustavson D. E. Fisher S. E.. (2022). The musical abilities, pleiotropy, language, and environment (MAPLE) framework for understanding musicality-language links across the lifespan. Neurobiol. Lang. 3, 615–664. doi: 10.1162/nol_a_00079 PMC9893227 36742012

[ref62] Neurobehavioral Systems, Inc. (2024). Presentation® software (18.0). Available online at: www.neurobs.com

[ref63] Ozernov-Palchik O. Patel A. D. (2018). Musical rhythm and reading development: does beat processing matter? Ann. N. Y. Acad. Sci. 1423, 166–175. doi: 10.1111/nyas.13853 29781084

[ref64] Patel A. D. (2003). Language, music, syntax and the brain. Nat Neurosci. 6, 674–681. doi: 10.1038/nn1082 12830158

[ref65] Patel A. D. (2008). Talk of the tone. Nature 453, 726–727. doi: 10.1038/453726a 18528382

[ref66] Patel A. D. (2011). Why would musical training benefit the neural encoding of speech? The OPERA hypothesis. Front. Psychol. 2, 1–14. doi: 10.3389/fpsyg.2011.00142 21747773 PMC3128244

[ref67] Patel A. D. (2012). The OPERA hypothesis: assumptions and clarifications. Ann. N. Y. Acad. Sci. 1252, 124–128. doi: 10.1111/j.1749-6632.2011.06426.x 22524349

[ref68] Patel A. D. (2014). Can nonlinguistic musical training change the way the brain processes speech? The expanded OPERA hypothesis. Hear. Res. 308, 98–108. doi: 10.1016/j.heares.2013.08.011 24055761

[ref69] Patel A. D. Daniele J. R. (2003). An empirical comparison of rhythm in language and music. Cognition 87, B35–B45. doi: 10.1016/s0010-0277(02)00187-7 12499110

[ref70] Patel A. D. Iversen J. R. (2014). The evolutionary neuroscience of musical beat perception: the action simulation for auditory prediction (ASAP) hypothesis. Front. Syst. Neurosci. 8, 1–14. doi: 10.3389/fnsys.2014.00057 24860439 PMC4026735

[ref71] Patel A. D. Morgan E. (2016). Exploring cognitive relations between prediction in language and music. Cogn. Sci. 41, 303–320. doi: 10.1111/cogs.12411 27665745

[ref72] Peirce J. Gray J. R. Simpson S. MacAskill M. Höchenberger R. Sogo H.. (2019). PsychoPy2: experiments in behavior made easy. Behav. Res. Methods 51, 195–203. doi: 10.3758/s13428-018-01193-y 30734206 PMC6420413

[ref73] Peretz I. (2009). Music, language and modularity framed in action. Psychol. Belg 49:157. doi: 10.5334/pb-49-2-3-157

[ref74] Peretz I. Coltheart M. (2003). Modularity of music processing. Nat. Neurosci. 6, 688–691. doi: 10.1038/nn1083 12830160

[ref75] Persici V. Blain S. D. Iversen J. R. Key A. P. Kotz S. A. Devin McAuley J.. (2023). Individual differences in neural markers of beat processing relate to spoken grammar skills in six-year-old children. Brain Lang. 246:105345. doi: 10.1016/j.bandl.2023.105345 37994830

[ref76] Poudrier E. (2020). The influence of rate and accentuation on subjective Rhythmization. Music. Percept. 38, 27–45. doi: 10.1525/mp.2020.38.1.27

[ref77] Przybylski L. Bedoin N. Krifi-Papoz S. Herbillon V. Roch D. Léculier L.. (2013). Rhythmic auditory stimulation influences syntactic processing in children with developmental language disorders. Neuropsychology 27, 121–131. doi: 10.1037/a0031277 23356600

[ref78] RC Team (2018). A language and environment for statistical computing. Vienna: R Foundation for Statistical Computing.

[ref79] Roncaglia-Denissen M. P. Schmidt-Kassow M. Heine A. Kotz S. A. (2014). On the impact of L2 speech rhythm on syntactic ambiguity resolution. Second. Lang. Res. 31, 157–178. doi: 10.1177/0267658314554497

[ref80] Roncaglia-denissen M. P. Schmidt-kassow M. Kotz S. A. (2013). Speech rhythm facilitates syntactic ambiguity resolution: ERP evidence. PLoS One 8, 1–9. doi: 10.1371/journal.pone.0056000 PMC3568096 23409109

[ref81] Rothermich K. Kotz S. A. (2013). Predictions in speech comprehension: FMRI evidence on the meter-semantic interface. NeuroImage 70, 89–100. doi: 10.1016/j.neuroimage.2012.12.013 23291188

[ref82] Rothermich K. Schmidt-Kassow M. Kotz S. A. (2012). Rhythm’s gonna get you: regular meter facilitates semantic sentence processing. Neuropsychologia 50, 232–244. doi: 10.1016/j.neuropsychologia.2011.10.025 22178743

[ref83] Schmidt-Kassow M. Rothermich K. Schwartze M. Kotz S. A. (2011). Did you get the beat? Late proficient French-German learners extract strong-weak patterns in tonal but not in linguistic sequences. NeuroImage 54, 568–576. doi: 10.1016/j.neuroimage.2010.07.062 20692349

[ref84] Schön D. Tillmann B. (2015). Short- and long-term rhythmic interventions: perspectives for language rehabilitation. Ann. N. Y. Acad. Sci. 1337, 32–39. doi: 10.1111/nyas.12635 25773614

[ref85] Schwartze M. Farrugia N. Kotz S. A. (2013). Dissociation of formal and temporal predictability in early auditory evoked potentials. Neuropsychologia 51, 320–325. doi: 10.1016/j.neuropsychologia.2012.09.037 23022431

[ref86] Schwartze M. Kotz S. A. (2016). Contributions of cerebellar event-based temporal processing and preparatory function to speech perception. Brain Lang. 161, 28–32. doi: 10.1016/j.bandl.2015.08.005 26362972

[ref87] Serratrice L. Sorace A. Filiaci F. Baldo M. (2009). Bilingual children’s sensitivity to specificity and genericity: evidence from metalinguistic awareness. Biling. Lang. Congn. 12, 239–257. doi: 10.1017/s1366728909004027

[ref88] Slater J. Kraus N. (2015). The role of rhythm in perceiving speech in noise: a comparison of percussionists, vocalists and non-musicians. Cogn. Process. 17, 79–87. doi: 10.1007/s10339-015-0740-7 26445880 PMC5019948

[ref89] Sprouse J. (2007). Continuous acceptability, categorical grammaticality, and experimental syntax. Biolinguistics 1, 123–134. doi: 10.5964/bioling.8597

[ref90] Stanislaw H. Todorov N. (1999). Calculation of signal detection theory measures. Behav. Res. Methods Instruments Computers 31, 137–149. doi: 10.3758/BF03207704 10495845

[ref91] Tal I. Large E. W. Rabinovitch E. Wei Y. Schroeder C. E. Poeppel D.. (2017). Neural entrainment to the beat: the “missing-pulse” phenomenon. J. Neurosci. 37, 6331–6341. doi: 10.1523/JNEUROSCI.2500-16.2017 28559379 PMC5490067

[ref92] Tierney A. Kraus N. (2014). Auditory-motor entrainment and phonological skills: precise auditory timing hypothesis (PATH). Front. Hum. Neurosci. 8, 1–9. doi: 10.3389/fnhum.2014.00949 25505879 PMC4245894

[ref93] Villata S. Franck J. (2020). Similarity-based interference in agreement comprehension and production: evidence from object agreement. J. Exp. Psychol. Learn. Mem. Cogn. 46, 170–188. doi: 10.1037/xlm0000718 31033310

[ref94] Villata S. Tabor W. Franck J. (2018). Encoding and retrieval interference in sentence comprehension: evidence from agreement. Front. Psychol. 9, 1–16. doi: 10.3389/fpsyg.2018.00002 29403414 PMC5780450

[ref95] Wagers M. W. Lau E. F. Phillips C. (2009). Agreement attraction in comprehension: representations and processes. J. Mem. Lang. 61, 206–237. doi: 10.1016/j.jml.2009.04.002

[ref96] Woodruff Carr K. White-Schwoch T. Tierney A. T. Strait D. L. Kraus N. (2014). Beat synchronization predicts neural speech encoding and reading readiness in preschoolers. Proc. Natl. Acad. Sci. 111, 14559–14564. doi: 10.1073/pnas.1406219111 25246562 PMC4210020

[ref97] Yates K. M. Moore D. R. Amitay S. Barry J. G. (2019). Sensitivity to melody, rhythm, and beat in supporting speech-in-noise perception in young adults. Ear Hear. 40, 358–367. doi: 10.1097/AUD.0000000000000621 29965864 PMC6400450

[ref98] Zentner M. Strauss H. (2017). Assessing musical ability quickly and objectively: development and validation of the short-PROMS and the Mini-PROMS. Ann N Y Acad Sci 1400, 33–45. doi: 10.1111/nyas.13410 28704888

