# Supplementary material for: Not primed to agree? Short or no effect of rhythmic priming on typical adults processing number agreement
Source: Front Psychol. 2025 Jun 13;16:1512267. doi: 10.3389/fpsyg.2025.1512267 (PMC12204084; doi:10.3389/fpsyg.2025.1512267)
Supplement: Supplementary file 1 [file Supplementary_file_1.docx]

**Supplementary Material**

| **Appendix 1: Natural language stimuli used in Experiments 1 and 3** | | | | |
| --- | --- | --- | --- | --- |
| **Item** | **Grammaticality** | **Sentence** | **Structure** |  |
| 1 | grammatical | Voici les personnes que le psychiatre reconstruit. | RO_OSV |  |
| 2 | ungrammatical | Voici les gorilles que la touriste décrivent. | RO_OSV |  |
| 3 | grammatical | Voici les soldats que la ministre promet. | RO_OSV |  |
| 4 | ungrammatical | Voici les amis que le vampire rejoignent. | RO_OSV |  |
| 5 | grammatical | Voici les génies que le prodige déçoit. | RO_OSV |  |
| 6 | ungrammatical | Voici les prêtresses que la princesse choisissent. | RO_OSV |  |
| 7 | grammatical | Voici les confrères que le savant comprend. | RO_OSV |  |
| 8 | ungrammatical | Voici les chameaux que le marquis détiennent. | RO_OSV |  |
| 9 | grammatical | Voici les voleurs que la mamie poursuit. | RO_OSV |  |
| 10 | ungrammatical | Voici les chinois que la française traduisent. | RO_OSV |  |
| 11 | grammatical | Voici les écrits que la tablette retranscrit. | RO_OSV |  |
| 12 | ungrammatical | Voici les baleines que la plongeuse défendent. | RO_OSV |  |
| 13 | grammatical | Voici les athlètes que la famille soutient. | RO_OSV |  |
| 14 | ungrammatical | Voici les immeubles que la mairie bâtissent. | RO_OSV |  |
| 15 | grammatical | Voici les élèves que la maîtresse détruit. | RO_OSV |  |
| 16 | ungrammatical | Voici les caissiers que la patronne suspendent. | RO_OSV |  |
| 17 | grammatical | Voici les bijoux que la boutique contient. | RO_OSV |  |
| 18 | ungrammatical | Voici les problèmes que la patience résolvent. | RO_OSV |  |
| 19 | grammatical | Voici les menteurs que le garçon punit. | RO_OSV |  |
| 20 | ungrammatical | Voici les messieurs que la pilote conduisent. | RO_OSV |  |
| 21 | grammatical | Voici les méduses que le nageur perçoit. | RO_OSV |  |
| 22 | ungrammatical | Voici les crapauds que le sculpteur reproduisent. | RO_OSV |  |
| 23 | grammatical | Voici les serpents que la sorcière nourrit. | RO_OSV |  |
| 24 | ungrammatical | Voici les aveugles que la souris salissent. | RO_OSV |  |
| 25 | grammatical | Voici les voyous que la complice prévient. | RO_OSV |  |
| 26 | ungrammatical | Voici les braqueurs que la fleuriste surprennent. | RO_OSV |  |
| 27 | grammatical | Voici les dragons que la guerrière combat. | RO_OSV |  |
| 28 | ungrammatical | Voici les brigands que le vigile reconnaissent. | RO_OSV |  |
| 29 | grammatical | Voici les accords que la justice rétablit. | RO_OSV |  |
| 30 | ungrammatical | Voici les poissons que le pêcheur garantissent. | RO_OSV |  |
| 31 | grammatical | Voici les poneys que le cocher rafraîchit. | RO_OSV |  |
| 32 | ungrammatical | Voici les chrétiens que le rabbin convertissent. | RO_OSV |  |
| 33 | grammatical | Voici les chanteurs que la batteuse compromet. | RO_OSV |  |
| 34 | ungrammatical | Voici les minables que la tireuse refroidissent. | RO_OSV |  |
| 35 | grammatical | Voici les limites que le traité définit. | RO_OSV |  |
| 36 | ungrammatical | Voici les bandits que la brigade contredisent. | RO_OSV |  |
| 37 | grammatical | Voici les amants que le bouffon divertit. | RO_OSV |  |
| 38 | ungrammatical | Voici les mémés que le médecin rajeunissent. | RO_OSV |  |
| 39 | grammatical | Voici les chansons que la radio raccourcit. | RO_OSV |  |
| 40 | ungrammatical | Voici les ados que le dealer pervertissent. | RO_OSV |  |
| 41 | grammatical | Voici les costumes que la lessive rétrécit. | RO_OSV |  |
| 42 | ungrammatical | Voici les infos que le rapports transmettent. | RO_OSV |  |
| 43 | grammatical | Voici les acteurs que la série travestit. | RO_OSV |  |
| 44 | ungrammatical | Voici les adjoints que le doyen réélisent. | RO_OSV |  |
| 45 | grammatical | Voici les sportifs que la blessée ralentit. | RO_OSV |  |
| 46 | ungrammatical | Voici les rivaux que le boxeur démolissent. | RO_OSV |  |
| 47 | grammatical | Voici les copains que la diva réunit. | RO_OSV |  |
| 48 | ungrammatical | Voici les marins que le stagiaire satisfont. | RO_OSV |  |
| 49 | grammatical | Voici la tortue que les vainqueurs obtiennent. | RO_OSV |  |
| 50 | ungrammatical | Voici la leçon que les chauffeurs apprend. | RO_OSV |  |
| 51 | grammatical | Voici le renard que les archers atteignent. | RO_OSV |  |
| 52 | ungrammatical | Voici le ménage que les enfants unit. | RO_OSV |  |
| 53 | grammatical | Voici la vaisselle que les lavages ternissent. | RO_OSV |  |
| 54 | ungrammatical | Voici le portrait que les médias dépeint. | RO_OSV |  |
| 55 | grammatical | Voici la frontière que les transports desservent. | RO_OSV |  |
| 56 | ungrammatical | Voici le métal que les produits dissout. | RO_OSV |  |
| 57 | grammatical | Voici la fillette que les époux attendent. | RO_OSV |  |
| 58 | ungrammatical | Voici le défaut que les miroirs grossit. | RO_OSV |  |
| 59 | grammatical | Voici la banquière que les escrocs corrompent. | RO_OSV |  |
| 60 | ungrammatical | Voici le marcheur que les masseurs pétrit. | RO_OSV |  |
| 61 | grammatical | Voici la couronne que les machines polissent. | RO_OSV |  |
| 62 | ungrammatical | Voici le poulet que les grands-pères farcit. | RO_OSV |  |
| 63 | grammatical | Voici la période que les congés finissent. | RO_OSV |  |
| 64 | ungrammatical | Voici le papier que les patients adjoint. | RO_OSV |  |
| 65 | grammatical | Voici la coupable que les victimes absolvent. | RO_OSV |  |
| 66 | ungrammatical | Voici le chaton que les félins admet. | RO_OSV |  |
| 67 | grammatical | Voici la bougie que les boissons éteignent. | RO_OSV |  |
| 68 | ungrammatical | Voici le fiston que les parents inscrit. | RO_OSV |  |
| 69 | grammatical | Voici la lectrice que les libraires instruisent. | RO_OSV |  |
| 70 | ungrammatical | Voici le lutin que les artistes repeint. | RO_OSV |  |
| 71 | grammatical | Voici la fortune que les articles prédisent. | RO_OSV |  |
| 72 | ungrammatical | Voici le plombier que les logeurs pressent. | RO_OSV |  |
| 73 | grammatical | Voici la médium que promeuvent les leaders. | RO_OVS |  |
| 74 | ungrammatical | Voici le pervers que proscrit les juristes. | RO_OVS |  |
| 75 | grammatical | Voici la cliente que rabattent les vendeurs. | RO_OVS |  |
| 76 | ungrammatical | Voici le chagrin que réduit les câlins. | RO_OVS |  |
| 77 | grammatical | Voici la terreur que répandent les canons. | RO_OVS |  |
| 78 | ungrammatical | Voici le champion qu'émeut les anglais. | RO_OVS |  |
| 79 | grammatical | Voici la bêtise qu'écrivent les journaux. | RO_OVS |  |
| 80 | ungrammatical | Voici le détail qu'omet les hommages. | RO_OVS |  |
| 81 | grammatical | Voici la tristesse qu'induisent les vengeances. | RO_OVS |  |
| 82 | ungrammatical | Voici le fumier qu'étend les tracteurs. | RO_OVS |  |
| 83 | grammatical | Voici la jury qu'endorment les témoins. | RO_OVS |  |
| 84 | ungrammatical | Voici le croyant que bénit les curés. | RO_OVS |  |
| 85 | grammatical | Voici la baronne que bannissent les duchesses. | RO_OVS |  |
| 86 | ungrammatical | Voici le lapin qu'abat les chasseurs. | RO_OVS |  |
| 87 | grammatical | Voici la brebis qu'entendent les indiens. | RO_OVS |  |
| 88 | ungrammatical | Voici le papi que contraint les truands. | RO_OVS |  |
| 89 | grammatical | Voici la gardienne qu'élisent les allemands. | RO_OVS |  |
| 90 | ungrammatical | Voici le liquide qu'emplit les bouteilles. | RO_OVS |  |
| 91 | grammatical | Voici la tueuse que blanchissent les gendarmes. | RO_OVS |  |
| 92 | ungrammatical | Voici le morceau qu'émet les enceintes. | RO_OVS |  |
| 93 | grammatical | Voici la jument que chérissent les anciens. | RO_OVS |  |
| 94 | ungrammatical | Voici le casier que durcit les vernis. | RO_OVS |  |
| 95 | grammatical | Voici la bestiole qu'étreignent les gamins. | RO_OVS |  |
| 96 | ungrammatical | Voici le malade que recoud les soignants. | RO_OVS |  |
| 97 | grammatical | Le papa met les assiettes. | SVO |  |
| 98 | ungrammatical | Le fidèle prennent les hiboux. | SVO |  |
| 99 | grammatical | Le robot fait les compotes. | SVO |  |
| 100 | ungrammatical | Le barbare rendent les lamas. | SVO |  |
| 101 | ungrammatical | Les cuistots sent la volaille. | SVO |  |
| 102 | grammatical | Les chaussures tordent la cheville. | SVO |  |
| 103 | grammatical | La maman bat les jumeaux. | SVO |  |
| 104 | ungrammatical | La tigresse mordent les agneaux. | SVO |  |
| 105 | ungrammatical | Les gagnants plaint le perdant. | SVO |  |
| 106 | grammatical | Les couteaux fendent le fromage. | SVO |  |
| 107 | ungrammatical | Les fumeurs joint le docteur. | SVO |  |
| 108 | grammatical | Les livreurs perdent le homard. | SVO |  |
| 109 | ungrammatical | Les mensonges défait le contrat. | SVO |  |
| 110 | grammatical | Les dompteurs brandissent le reptile. | SVO |  |
| 111 | grammatical | Le manchot conçoit les bébés. | SVO |  |
| 112 | ungrammatical | Le commerce remettent les échanges. | SVO |  |
| 113 | ungrammatical | Les efforts permet la victoire. | SVO |  |
| 114 | grammatical | Les gérants entretiennent la mendiante . | SVO |  |
| 115 | grammatical | La pédiatre intervertit les marmots. | SVO |  |
| 116 | ungrammatical | La nana aperçoivent les oiseaux. | SVO |  |
| 117 | ungrammatical | Les pirates soumet la bourgeoise. | SVO |  |
| 118 | grammatical | Les seigneurs anoblissent la bâtarde. | SVO |  |
| 119 | ungrammatical | La ponceuse arrondissent les arêtes. | SVO |  |
| 120 | grammatical | La clinique maintient les actions. | SVO |  |
| 121 | grammatical | Les grands-mères disent que le petit lit. | IND_INTR |  |
| 122 | ungrammatical | Les serveurs disent que le saumon cuisent. | IND_INTR |  |
| 123 | ungrammatical | La gangster dit que les motards suit. | IND_INTR |  |
| 124 | grammatical | La nourrice dit que les altesses cousent. | IND_INTR |  |
| 125 | ungrammatical | Le public dit que les agents boit. | IND_INTR |  |
| 126 | grammatical | Le concierge dit que les voisins rompent. | IND_INTR |  |
| 127 | grammatical | Les neveux disent que le hamster grandit. | IND_INTR |  |
| 128 | ungrammatical | Les passants disent que la coureuse rougissent. | IND_INTR |  |
| 129 | grammatical | La modèle dit que les experts applaudissent. | IND_INTR |  |
| 130 | grammatical | Les vieillards disent que la fiancé resplendit. | IND_INTR |  |
| 131 | ungrammatical | Les valets disent que la tsarine boivent. | IND_INTR |  |
| 132 | ungrammatical | La belle-mère dit que les canards épaissit. | IND_INTR |  |
| 133 | ungrammatical | Voici les frangins qui veut le caniche. | RS |  |
| 134 | ungrammatical | Voici le tricheur qui craignent les arbitres. | RS |  |
| 135 | ungrammatical | Voici les bourreaux qui pend la martyre. | RS |  |
| 136 | grammatical | Voici les coiffeuses qui teignent la danseuse. | RS |  |
| 137 | grammatical | Voici le bonhomme qui tient les chevaux. | RS |  |
| 138 | ungrammatical | Voici le fermier qui tondent les moutons. | RS |  |
| 139 | grammatical | Voici le sergent qui interrompt les intrus. | RS |  |
| 140 | grammatical | Voici les prophètes qui avertissent le calife. | RS |  |
| 141 | grammatical | Voici la rebelle qui affaiblit les déesses. | RS |  |
| 142 | ungrammatical | Voici la méchante qui retiennent les otages. | RS |  |
| 143 | ungrammatical | Voici les rouleaux qui aplatit la pizza. | RS |  |
| 144 | grammatical | Voici les engins qui élargissent le chemin. | RS |  |
| **Appendix 1. Linguistic stimuli used in Experiments 1 and 3. The pseudowords used by György et al. (2024) were replaced by French words, controlling for a range of variables (see Methods). Syntactic structures are coded as follows: SVO - simple subject-verb-object sentences, RS - subject relatives, IND_INTR - complement clauses, RO_OSV - object-subject-verb (common) object relatives, RO_OSV object-verb-subject (transposed) obejct relatives (these were removed from analysis).** | | | | |

| **Appendix 2: Jabberwocky stimuli used in Experiment 2** | | | | |
| --- | --- | --- | --- | --- |
| **Item** | **Grammaticality** | **Sentence** | **Structure** |  |
| 1 | grammatical | Voici les dafrans que le bostron reconstruit. | RO_OSV |  |
| 2 | ungrammatical | Voici les mitelles que la branille décrivent. | RO_OSV |  |
| 3 | grammatical | Voici les lamolles que la chibute promet. | RO_OSV |  |
| 4 | ungrammatical | Voici les lestans que le firon rejoignent. | RO_OSV |  |
| 5 | grammatical | Voici les bounieurs que le rissiat déçoit. | RO_OSV |  |
| 6 | ungrammatical | Voici les trafines que la narmule choisissent. | RO_OSV |  |
| 7 | grammatical | Voici les chibaires que le rabou comprend. | RO_OSV |  |
| 8 | ungrammatical | Voici les pomins que le batil détiennent. | RO_OSV |  |
| 9 | grammatical | Voici les strivelles que la bracune poursuit. | RO_OSV |  |
| 10 | ungrammatical | Voici les pocrous que la romache traduisent. | RO_OSV |  |
| 11 | grammatical | Voici les ramuches que la mioline retranscrit. | RO_OSV |  |
| 12 | ungrammatical | Voici les poutames que la boulume défendent. | RO_OSV |  |
| 13 | grammatical | Voici les lucans que la curame soutient. | RO_OSV |  |
| 14 | ungrammatical | Voici les remiches que la suroise bâtissent. | RO_OSV |  |
| 15 | grammatical | Voici les caquettes que la bacolle détruit. | RO_OSV |  |
| 16 | ungrammatical | Voici les tumiles que la ramule suspendent. | RO_OSV |  |
| 17 | grammatical | Voici les chetrilles que la crofelle contient. | RO_OSV |  |
| 18 | ungrammatical | Voici les tétunes que la simale résolvent. | RO_OSV |  |
| 19 | grammatical | Voici les trabins que le tarmon punit. | RO_OSV |  |
| 20 | ungrammatical | Voici les fipules que la guivore conduisent. | RO_OSV |  |
| 21 | grammatical | Voici les mibans que le drepou perçoit. | RO_OSV |  |
| 22 | ungrammatical | Voici les mertins que le quimard reproduisent. | RO_OSV |  |
| 23 | grammatical | Voici les ramines que la crapouse nourrit. | RO_OSV |  |
| 24 | ungrammatical | Voici les chimates que la ramoule salissent. | RO_OSV |  |
| 25 | grammatical | Voici les tripouilles que la mupure prévient. | RO_OSV |  |
| 26 | ungrammatical | Voici les fergères que la nitoise surprennent. | RO_OSV |  |
| 27 | grammatical | Voici les bamilles que la pimesse combat. | RO_OSV |  |
| 28 | ungrammatical | Voici les muchons que le canouge reconnaissent. | RO_OSV |  |
| 29 | grammatical | Voici les marettes que la mivale rétablit. | RO_OSV |  |
| 30 | ungrammatical | Voici les mirtons que le sarpou garantissent. | RO_OSV |  |
| 31 | grammatical | Voici les nerfils que le cardat rafraîchit. | RO_OSV |  |
| 32 | ungrammatical | Voici les bassions que le gupan convertissent. | RO_OSV |  |
| 33 | grammatical | Voici les droupennes que la manule compromet. | RO_OSV |  |
| 34 | ungrammatical | Voici les pilaines que la drofette refroidissent. | RO_OSV |  |
| 35 | grammatical | Voici les ponchons que le fiteau définit. | RO_OSV |  |
| 36 | ungrammatical | Voici les jamites que la sottelle contredisent. | RO_OSV |  |
| 37 | grammatical | Voici les tusteurs que le chamoul divertit. | RO_OSV |  |
| 38 | ungrammatical | Voici les pesclains que le farton rajeunissent. | RO_OSV |  |
| 39 | grammatical | Voici les prastilles que la cartanne raccourcit. | RO_OSV |  |
| 40 | ungrammatical | Voici les vribons que le perchain pervertissent. | RO_OSV |  |
| 41 | grammatical | Voici les doidelles que la certesse rétrécit. | RO_OSV |  |
| 42 | ungrammatical | Voici les cortiers que le muchoin transmettent. | RO_OSV |  |
| 43 | grammatical | Voici les poinettes que la casarpe travestit. | RO_OSV |  |
| 44 | ungrammatical | Voici les chapirs que le juspan réélisent. | RO_OSV |  |
| 45 | grammatical | Voici les tranilles que la pancasse ralentit. | RO_OSV |  |
| 46 | ungrammatical | Voici les forcols que le bécoin démolissent. | RO_OSV |  |
| 47 | grammatical | Voici les civrelles que la fameille réunit. | RO_OSV |  |
| 48 | ungrammatical | Voici les dantous que le tranqueau satisfont. | RO_OSV |  |
| 49 | grammatical | Voici la pinole que les gipannes obtiennent. | RO_OSV |  |
| 50 | ungrammatical | Voici la mirtule que les ganettes apprend. | RO_OSV |  |
| 51 | grammatical | Voici le framan que les poiteaux atteignent. | RO_OSV |  |
| 52 | ungrammatical | Voici le porchon que les coutards unit. | RO_OSV |  |
| 53 | grammatical | Voici la soupille que les toirules ternissent. | RO_OSV |  |
| 54 | ungrammatical | Voici le vitron que les pasquerts dépeint. | RO_OSV |  |
| 55 | grammatical | Voici la roteille que les dachelles desservent. | RO_OSV |  |
| 56 | ungrammatical | Voici le plicot que les fandols dissout. | RO_OSV |  |
| 57 | grammatical | Voici la faïette que les lusannes attendent. | RO_OSV |  |
| 58 | ungrammatical | Voici le panclusse que les rochouls grossit. | RO_OSV |  |
| 59 | grammatical | Voici la gisture que les dramanes corrompent. | RO_OSV |  |
| 60 | ungrammatical | Voici le folon que les moudards pétrit. | RO_OSV |  |
| 61 | grammatical | Voici la cibanne que les roumettes polissent. | RO_OSV |  |
| 62 | ungrammatical | Voici le dardoin que les proutons farcit. | RO_OSV |  |
| 63 | grammatical | Voici la plimace que les feussines finissent. | RO_OSV |  |
| 64 | ungrammatical | Voici le clantain que les teillons adjoint. | RO_OSV |  |
| 65 | grammatical | Voici la mitroise que les frenettes absolvent. | RO_OSV |  |
| 66 | ungrammatical | Voici le cetrard que les chistons admet. | RO_OSV |  |
| 67 | grammatical | Voici la planesse que les cramaches éteignent. | RO_OSV |  |
| 68 | ungrammatical | Voici le péneuil que les plissacs inscrit. | RO_OSV |  |
| 69 | grammatical | Voici la vesace que les plamoires instruisent. | RO_OSV |  |
| 70 | ungrammatical | Voici le reclon que les poutucs repeint. | RO_OSV |  |
| 71 | grammatical | Voici la passure que les chernières prédisent. | RO_OSV |  |
| 72 | ungrammatical | Voici le fristoin que les classacs pressent. | RO_OSV |  |
| 73 | grammatical | Voici la lantine que promeuvent les berleuses. | RO_OVS |  |
| 74 | ungrammatical | Voici le naraque que proscrit les pimoins. | RO_OVS |  |
| 75 | grammatical | Voici la nipoire que rabattent les sorlottes. | RO_OVS |  |
| 76 | ungrammatical | Voici le crapoin que réduit les serleurs. | RO_OVS |  |
| 77 | grammatical | Voici la bémuche que répandent les parcades. | RO_OVS |  |
| 78 | ungrammatical | Voici le souchard qu'émeut les doupieux. | RO_OVS |  |
| 79 | grammatical | Voici la vernique qu'écrivent les sambases. | RO_OVS |  |
| 80 | ungrammatical | Voici le tralan qu'omet les gidrains. | RO_OVS |  |
| 81 | grammatical | Voici la cartige qu'induisent les gostelles. | RO_OVS |  |
| 82 | ungrammatical | Voici le quinsteau qu'étend les poulacs. | RO_OVS |  |
| 83 | grammatical | Voici la sénelle qu'endorment les gistures. | RO_OVS |  |
| 84 | ungrammatical | Voici le crispan que bénit les ratards. | RO_OVS |  |
| 85 | grammatical | Voici la tournille que bannissent les gueunières. | RO_OVS |  |
| 86 | ungrammatical | Voici le talpon qu'abat les clasteurs. | RO_OVS |  |
| 87 | grammatical | Voici la rinette qu'entendent les clutines. | RO_OVS |  |
| 88 | ungrammatical | Voici le cimique que contraint les brunels. | RO_OVS |  |
| 89 | grammatical | Voici la tisière qu'élisent les flaponnes. | RO_OVS |  |
| 90 | ungrammatical | Voici le moineur qu'emplit les pitandres. | RO_OVS |  |
| 91 | grammatical | Voici la démuse que blanchissent les gidoises. | RO_OVS |  |
| 92 | ungrammatical | Voici le rochetot qu'émet les pilberts. | RO_OVS |  |
| 93 | grammatical | Voici la lanchesse que chérissent les barlines. | RO_OVS |  |
| 94 | ungrammatical | Voici le timoul que durcit les charboits. | RO_OVS |  |
| 95 | grammatical | Voici la trouneille qu'étreignent les daleines. | RO_OVS |  |
| 96 | ungrammatical | Voici le piscard que recoud les tramieux. | RO_OVS |  |
| 97 | grammatical | Le lampoin met les mircats. | SVO |  |
| 98 | ungrammatical | Le trophone prennent les titons. | SVO |  |
| 99 | grammatical | Le biblon fait les xylones. | SVO |  |
| 100 | ungrammatical | Le tuma rendent les liteurs. | SVO |  |
| 101 | ungrammatical | Las critones sent la blatère. | SVO |  |
| 102 | grammatical | Les diderines tordent la varouche. | SVO |  |
| 103 | grammatical | La tésiade bat les moxines. | SVO |  |
| 104 | ungrammatical | La talude mordent les zirlones. | SVO |  |
| 105 | ungrammatical | Les clapoirs plaint le carfi. | SVO |  |
| 106 | grammatical | Les dépiets fendent le frépon. | SVO |  |
| 107 | ungrammatical | Les dapons joint le chailleul. | SVO |  |
| 108 | grammatical | Les lumoirs perdent le tudruc. | SVO |  |
| 109 | ungrammatical | Les tachins défait le panoir. | SVO |  |
| 110 | grammatical | Les fipons brandissent le soulpucre. | SVO |  |
| 111 | grammatical | Le drapal conçoit les fignons. | SVO |  |
| 112 | ungrammatical | Le tassin remettent les charpans. | SVO |  |
| 113 | ungrammatical | Les bassiches permet la ragille. | SVO |  |
| 114 | grammatical | Les datilles entretiennent la castage. | SVO |  |
| 115 | grammatical | La pétaille intervertit les taillaches. | SVO |  |
| 116 | ungrammatical | La fourmelle aperçoivent les vampales. | SVO |  |
| 117 | ungrammatical | Les courlettes soumet la parlène. | SVO |  |
| 118 | grammatical | Les menouilles anoblissent la listule. | SVO |  |
| 119 | ungrammatical | La factale arrondissent les gisales. | SVO |  |
| 120 | grammatical | La coulaille maintient les rivielles. | SVO |  |
| 121 | grammatical | Les naillons disent que le chanin lit. | IND_INTR |  |
| 122 | ungrammatical | Les michons disent que le carnout cuisent. | IND_INTR |  |
| 123 | ungrammatical | La varlaille dit que les marlunes suit. | IND_INTR |  |
| 124 | grammatical | La gobette dit que les larmonnes cousent. | IND_INTR |  |
| 125 | ungrammatical | Le prouchain dit que les parleaux boit. | IND_INTR |  |
| 126 | grammatical | Le gostar dit que les stifons rompent. | IND_INTR |  |
| 127 | grammatical | Les pistères disent que le trémieux saisit. | IND_INTR |  |
| 128 | ungrammatical | Les tréchains disent que la pimande rougissent. | IND_INTR |  |
| 129 | grammatical | La cultine dit que les crapilles applaudissent. | IND_INTR |  |
| 130 | grammatical | Les funules disent que la chouturne resplendit. | IND_INTR |  |
| 131 | ungrammatical | Les postilles disent que la cranule boivent. | IND_INTR |  |
| 132 | ungrammatical | La peillette dit que les misailles épaissit. | IND_INTR |  |
| 133 | ungrammatical | Voici les forcaux qui veut le stanon. | RS |  |
| 134 | ungrammatical | Voici le stélo qui craignent les pécoins. | RS |  |
| 135 | ungrammatical | Voici les modilles qui pend la charasse. | RS |  |
| 136 | grammatical | Voici les salames qui teignent la plichette. | RS |  |
| 137 | grammatical | Voici le donchon qui tient les chapoils. | RS |  |
| 138 | ungrammatical | Voici le flumeau qui tondent les terlacs. | RS |  |
| 139 | grammatical | Voici le frimin qui interrompt les crastoirs. | RS |  |
| 140 | grammatical | Voici les chageots qui avertissent le ficard. | RS |  |
| 141 | grammatical | Voici la manoule qui affaiblit les flimousses. | RS |  |
| 142 | ungrammatical | Voici la trimale qui retiennent les sompiges. | RS |  |
| 143 | ungrammatical | Voici les lichales qui aplatit la malitre. | RS |  |
| 144 | grammatical | Voici les terluettes qui élargissent le chifandre. | RS |  |
| **Appendix 2. Linguistic stimuli used in Experiment 2, identical to the material used by György et al. (2024). Syntactic structures are coded as follows: SVO - simple subject-verb-object sentences, RS - subject relatives, IND_INTR - complement clauses, RO_OSV - object-subject-verb (common) object relatives, RO_OSV object-verb-subject (transposed) obejct relatives (these were removed from analysis).** | | | | |

| Subject | Prime | nhits | nfalsealrams | nmiss | ncr |
| --- | --- | --- | --- | --- | --- |
| 20303228 | Regular | 18 | 4 | 1 | 16 |
| 20303228 | Silence | 20 | 1 | 0 | 19 |
| 20303228 | Irregular | 19 | 1 | 0 | 18 |
| A10303 | Regular | 19 | 0 | 0 | 20 |
| A10303 | Silence | 17 | 2 | 3 | 18 |
| A10303 | Irregular | 18 | 0 | 1 | 19 |
| A10304 | Regular | 11 | 7 | 9 | 13 |
| A10304 | Silence | 12 | 3 | 7 | 17 |
| A10304 | Irregular | 13 | 3 | 6 | 16 |
| A10307 | Regular | 5 | 3 | 14 | 17 |
| A10307 | Silence | 7 | 3 | 12 | 16 |
| A10307 | Irregular | 10 | 3 | 10 | 17 |
| A10308 | Regular | 13 | 4 | 6 | 15 |
| A10308 | Silence | 13 | 3 | 7 | 17 |
| A10308 | Irregular | 13 | 4 | 6 | 16 |
| A10310 | Regular | 17 | 0 | 2 | 19 |
| A10310 | Silence | 18 | 0 | 2 | 20 |
| A10310 | Irregular | 17 | 0 | 2 | 20 |
| A10311 | Regular | 19 | 0 | 1 | 20 |
| A10311 | Silence | 17 | 1 | 2 | 18 |
| A10311 | Irregular | 17 | 0 | 2 | 20 |
| A10312 | Regular | 18 | 1 | 1 | 19 |
| A10312 | Silence | 17 | 2 | 2 | 17 |
| A10312 | Irregular | 20 | 0 | 0 | 20 |
| A10313 | Regular | 17 | 0 | 2 | 20 |
| A10313 | Silence | 17 | 0 | 2 | 19 |
| A10313 | Irregular | 15 | 0 | 5 | 20 |
| A10315 | Regular | 19 | 1 | 0 | 18 |
| A10315 | Silence | 17 | 1 | 2 | 19 |
| A10315 | Irregular | 19 | 1 | 1 | 19 |
| A10316 | Regular | 15 | 1 | 4 | 19 |
| A10316 | Silence | 17 | 3 | 2 | 16 |
| A10316 | Irregular | 16 | 1 | 4 | 19 |
| A10317 | Regular | 13 | 3 | 6 | 17 |
| A10317 | Silence | 15 | 1 | 4 | 18 |
| A10317 | Irregular | 17 | 3 | 3 | 17 |
| A10318 | Regular | 16 | 2 | 4 | 18 |
| A10318 | Silence | 14 | 1 | 5 | 18 |
| A10318 | Irregular | 15 | 1 | 4 | 19 |
| A10320 | Regular | 18 | 1 | 1 | 18 |
| A10320 | Silence | 17 | 1 | 3 | 19 |
| A10320 | Irregular | 18 | 1 | 1 | 19 |
| A10321 | Regular | 16 | 1 | 4 | 19 |
| A10321 | Silence | 19 | 0 | 0 | 19 |
| A10321 | Irregular | 18 | 0 | 1 | 20 |
| A10323 | Regular | 11 | 3 | 8 | 16 |
| A10323 | Silence | 8 | 4 | 11 | 16 |
| A10323 | Irregular | 7 | 5 | 13 | 15 |
| A10324 | Regular | 17 | 1 | 3 | 19 |
| A10324 | Silence | 18 | 0 | 1 | 19 |
| A10324 | Irregular | 18 | 0 | 1 | 20 |
| A10325 | Regular | 20 | 5 | 0 | 15 |
| A10325 | Silence | 19 | 3 | 0 | 17 |
| A10325 | Irregular | 16 | 2 | 3 | 17 |
| A10326 | Regular | 15 | 5 | 5 | 15 |
| A10326 | Silence | 14 | 2 | 5 | 17 |
| A10326 | Irregular | 16 | 1 | 3 | 19 |
| A10327 | Regular | 17 | 1 | 2 | 18 |
| A10327 | Silence | 17 | 1 | 3 | 19 |
| A10327 | Irregular | 19 | 1 | 0 | 19 |
| A10328 | Regular | 20 | 2 | 0 | 18 |
| A10328 | Silence | 19 | 1 | 0 | 18 |
| A10328 | Irregular | 18 | 1 | 1 | 19 |
| A10330 | Regular | 19 | 2 | 0 | 18 |
| A10330 | Silence | 20 | 0 | 0 | 20 |
| A10330 | Irregular | 17 | 0 | 2 | 19 |
| A10331 | Regular | 15 | 1 | 4 | 18 |
| A10331 | Silence | 19 | 1 | 1 | 19 |
| A10331 | Irregular | 16 | 1 | 3 | 19 |
| A10332 | Regular | 19 | 1 | 0 | 18 |
| A10332 | Silence | 18 | 0 | 1 | 20 |
| A10332 | Irregular | 17 | 0 | 3 | 20 |
| A10333 | Regular | 18 | 1 | 1 | 18 |
| A10333 | Silence | 19 | 0 | 0 | 20 |
| A10333 | Irregular | 18 | 1 | 2 | 19 |
| A10336 | Regular | 19 | 2 | 0 | 17 |
| A10336 | Silence | 18 | 5 | 1 | 15 |
| A10336 | Irregular | 18 | 2 | 2 | 18 |
| A10337 | Regular | 16 | 9 | 4 | 11 |
| A10337 | Silence | 14 | 6 | 5 | 14 |
| A10337 | Irregular | 10 | 7 | 9 | 12 |
| A10339 | Regular | 18 | 2 | 1 | 18 |
| A10339 | Silence | 18 | 1 | 2 | 19 |
| A10339 | Irregular | 17 | 1 | 2 | 18 |
| A10340 | Regular | 18 | 0 | 1 | 19 |
| A10340 | Silence | 16 | 1 | 4 | 19 |
| A10340 | Irregular | 16 | 3 | 3 | 17 |
| A10342 | Regular | 17 | 0 | 2 | 20 |
| A10342 | Silence | 17 | 0 | 2 | 19 |
| A10342 | Irregular | 19 | 0 | 1 | 20 |
| A10343 | Regular | 34 | 4 | 4 | 36 |
| A10343 | Silence | 33 | 8 | 7 | 32 |
| A10343 | Irregular | 32 | 2 | 6 | 36 |
| A10344 | Regular | 15 | 2 | 5 | 18 |
| A10344 | Silence | 12 | 0 | 7 | 19 |
| A10344 | Irregular | 14 | 1 | 5 | 19 |
| A10346 | Regular | 19 | 1 | 0 | 19 |
| A10346 | Silence | 20 | 0 | 0 | 20 |
| A10346 | Irregular | 18 | 0 | 1 | 19 |
| A10347 | Regular | 19 | 0 | 0 | 20 |
| A10347 | Silence | 18 | 0 | 2 | 20 |
| A10347 | Irregular | 19 | 0 | 0 | 19 |
| A10349 | Regular | 20 | 1 | 0 | 19 |
| A10349 | Silence | 18 | 3 | 1 | 16 |
| A10349 | Irregular | 17 | 0 | 2 | 20 |
| A10350 | Regular | 18 | 1 | 1 | 18 |
| A10350 | Silence | 20 | 2 | 0 | 18 |
| A10350 | Irregular | 19 | 0 | 0 | 20 |
| A10351 | Regular | 20 | 0 | 0 | 20 |
| A10351 | Silence | 18 | 0 | 1 | 19 |
| A10351 | Irregular | 19 | 1 | 0 | 19 |
| A10352 | Regular | 19 | 1 | 0 | 19 |
| A10352 | Silence | 20 | 0 | 0 | 20 |
| A10352 | Irregular | 18 | 0 | 1 | 19 |
| A10353 | Regular | 15 | 1 | 4 | 19 |
| A10353 | Silence | 17 | 3 | 3 | 17 |
| A10353 | Irregular | 14 | 4 | 5 | 15 |
| A10354 | Regular | 18 | 0 | 1 | 20 |
| A10354 | Silence | 19 | 1 | 0 | 18 |
| A10354 | Irregular | 19 | 0 | 1 | 20 |
| A10355 | Regular | 17 | 3 | 2 | 17 |
| A10355 | Silence | 18 | 3 | 1 | 16 |
| A10355 | Irregular | 16 | 2 | 4 | 18 |
| A10356 | Regular | 15 | 1 | 4 | 19 |
| A10356 | Silence | 14 | 2 | 6 | 18 |
| A10356 | Irregular | 18 | 0 | 1 | 19 |
| A10358 | Regular | 18 | 1 | 1 | 18 |
| A10358 | Silence | 18 | 3 | 1 | 17 |
| A10358 | Irregular | 15 | 0 | 5 | 20 |
| A10359 | Regular | 16 | 3 | 4 | 17 |
| A10359 | Silence | 16 | 5 | 3 | 14 |
| A10359 | Irregular | 16 | 1 | 3 | 19 |
| A10360 | Regular | 17 | 1 | 2 | 19 |
| A10360 | Silence | 19 | 1 | 0 | 18 |
| A10360 | Irregular | 20 | 2 | 0 | 18 |
| A10361 | Regular | 17 | 2 | 2 | 18 |
| A10361 | Silence | 16 | 0 | 3 | 19 |
| A10361 | Irregular | 18 | 1 | 2 | 19 |
| A10362 | Regular | 15 | 2 | 4 | 18 |
| A10362 | Silence | 14 | 2 | 6 | 18 |
| A10362 | Irregular | 14 | 2 | 5 | 17 |
| A10363 | Regular | 19 | 0 | 0 | 20 |
| A10363 | Silence | 18 | 0 | 2 | 20 |
| A10363 | Irregular | 17 | 3 | 2 | 16 |
| A10364 | Regular | 17 | 1 | 3 | 19 |
| A10364 | Silence | 17 | 2 | 2 | 18 |
| A10364 | Irregular | 18 | 1 | 1 | 18 |
| A10365 | Regular | 19 | 0 | 1 | 20 |
| A10365 | Silence | 19 | 1 | 0 | 19 |
| A10365 | Irregular | 17 | 0 | 2 | 19 |
| A10366 | Regular | 18 | 0 | 2 | 20 |
| A10366 | Silence | 19 | 1 | 0 | 18 |
| A10366 | Irregular | 18 | 0 | 1 | 20 |
| A10368 | Regular | 19 | 1 | 0 | 19 |
| A10368 | Silence | 13 | 2 | 6 | 17 |
| A10368 | Irregular | 12 | 1 | 8 | 19 |
| A10369 | Regular | 7 | 3 | 13 | 17 |
| A10369 | Silence | 8 | 1 | 11 | 19 |
| A10369 | Irregular | 6 | 1 | 13 | 18 |
| A10370 | Regular | 18 | 2 | 2 | 18 |
| A10370 | Silence | 16 | 1 | 3 | 18 |
| A10370 | Irregular | 18 | 1 | 1 | 19 |
| A10371 | Regular | 18 | 0 | 1 | 19 |
| A10371 | Silence | 18 | 1 | 1 | 19 |
| A10371 | Irregular | 20 | 1 | 0 | 19 |
| A10372 | Regular | 20 | 0 | 0 | 20 |
| A10372 | Silence | 19 | 0 | 0 | 20 |
| A10372 | Irregular | 19 | 1 | 0 | 18 |
| A10373 | Regular | 17 | 0 | 3 | 20 |
| A10373 | Silence | 18 | 1 | 1 | 18 |
| A10373 | Irregular | 19 | 0 | 0 | 20 |
| A10374 | Regular | 19 | 1 | 1 | 19 |
| A10374 | Silence | 19 | 0 | 0 | 19 |
| A10374 | Irregular | 19 | 1 | 0 | 19 |
| A10375 | Regular | 18 | 3 | 1 | 16 |
| A10375 | Silence | 18 | 2 | 2 | 18 |
| A10375 | Irregular | 16 | 0 | 3 | 20 |
| A10377 | Regular | 18 | 0 | 1 | 19 |
| A10377 | Silence | 19 | 1 | 0 | 19 |
| A10377 | Irregular | 18 | 1 | 2 | 19 |
| A10378 | Regular | 18 | 1 | 1 | 19 |
| A10378 | Silence | 17 | 1 | 2 | 18 |
| A10378 | Irregular | 16 | 1 | 4 | 19 |
| A10379 | Regular | 18 | 2 | 1 | 17 |
| A10379 | Silence | 16 | 1 | 3 | 19 |
| A10379 | Irregular | 16 | 3 | 4 | 17 |
| A10380 | Regular | 19 | 3 | 0 | 17 |
| A10380 | Silence | 18 | 1 | 1 | 18 |
| A10380 | Irregular | 17 | 3 | 3 | 17 |
| A10381 | Regular | 20 | 0 | 0 | 20 |
| A10381 | Silence | 19 | 0 | 0 | 20 |
| A10381 | Irregular | 18 | 0 | 1 | 19 |
| A10382 | Regular | 14 | 1 | 5 | 18 |
| A10382 | Silence | 16 | 7 | 3 | 13 |
| A10382 | Irregular | 13 | 2 | 7 | 18 |
| A10383 | Regular | 19 | 2 | 0 | 17 |
| A10383 | Silence | 18 | 3 | 1 | 17 |
| A10383 | Irregular | 18 | 4 | 2 | 16 |
| A10384 | Regular | 19 | 0 | 0 | 19 |
| A10384 | Silence | 19 | 0 | 0 | 20 |
| A10384 | Irregular | 19 | 1 | 1 | 19 |
| A10386 | Regular | 19 | 0 | 1 | 20 |
| A10386 | Silence | 18 | 1 | 1 | 18 |
| A10386 | Irregular | 19 | 0 | 0 | 20 |
| A10387 | Regular | 17 | 1 | 3 | 19 |
| A10387 | Silence | 15 | 1 | 4 | 19 |
| A10387 | Irregular | 15 | 0 | 4 | 19 |
| A10388 | Regular | 14 | 1 | 6 | 19 |
| A10388 | Silence | 15 | 1 | 4 | 18 |
| A10388 | Irregular | 19 | 0 | 0 | 20 |
| A10389 | Regular | 16 | 2 | 4 | 18 |
| A10389 | Silence | 12 | 0 | 7 | 19 |
| A10389 | Irregular | 11 | 5 | 8 | 15 |
| A10390 | Regular | 19 | 2 | 0 | 18 |
| A10390 | Silence | 19 | 0 | 1 | 20 |
| A10390 | Irregular | 18 | 1 | 1 | 18 |
| A10391 | Regular | 14 | 0 | 5 | 20 |
| A10391 | Silence | 15 | 2 | 5 | 18 |
| A10391 | Irregular | 12 | 2 | 7 | 17 |
| A10392 | Regular | 12 | 2 | 8 | 18 |
| A10392 | Silence | 10 | 1 | 9 | 19 |
| A10392 | Irregular | 9 | 4 | 10 | 15 |
| A10393 | Regular | 17 | 3 | 2 | 16 |
| A10393 | Silence | 15 | 0 | 5 | 20 |
| A10393 | Irregular | 18 | 1 | 1 | 19 |
| A10394 | Regular | 18 | 0 | 2 | 20 |
| A10394 | Silence | 18 | 1 | 1 | 19 |
| A10394 | Irregular | 19 | 0 | 0 | 19 |
| A10395 | Regular | 17 | 1 | 2 | 18 |
| A10395 | Silence | 17 | 1 | 3 | 19 |
| A10395 | Irregular | 17 | 0 | 2 | 20 |
| A10396 | Regular | 18 | 0 | 1 | 19 |
| A10396 | Silence | 16 | 1 | 4 | 19 |
| A10396 | Irregular | 19 | 0 | 0 | 20 |
| A10397 | Regular | 17 | 3 | 2 | 17 |
| A10397 | Silence | 19 | 1 | 0 | 18 |
| A10397 | Irregular | 19 | 0 | 1 | 20 |
| A10398 | Regular | 18 | 2 | 2 | 18 |
| A10398 | Silence | 19 | 0 | 0 | 20 |
| A10398 | Irregular | 16 | 0 | 3 | 19 |
| A10399 | Regular | 19 | 1 | 1 | 19 |
| A10399 | Silence | 19 | 0 | 0 | 20 |
| A10399 | Irregular | 19 | 1 | 0 | 18 |
| A10400 | Regular | 19 | 0 | 0 | 19 |
| A10400 | Silence | 18 | 0 | 1 | 20 |
| A10400 | Irregular | 18 | 0 | 2 | 20 |
| A10401 | Regular | 18 | 0 | 1 | 19 |
| A10401 | Silence | 15 | 2 | 5 | 18 |
| A10401 | Irregular | 16 | 1 | 3 | 19 |
| A10402 | Regular | 18 | 1 | 1 | 18 |
| A10402 | Silence | 18 | 0 | 2 | 20 |
| A10402 | Irregular | 17 | 1 | 2 | 19 |
| A10403 | Regular | 18 | 0 | 1 | 19 |
| A10403 | Silence | 18 | 4 | 2 | 16 |
| A10403 | Irregular | 19 | 4 | 0 | 16 |
| A10404 | Regular | 17 | 0 | 2 | 20 |
| A10404 | Silence | 18 | 1 | 2 | 19 |
| A10404 | Irregular | 18 | 1 | 1 | 18 |
| A10406 | Regular | 16 | 1 | 3 | 18 |
| A10406 | Silence | 17 | 3 | 2 | 17 |
| A10406 | Irregular | 13 | 3 | 7 | 17 |
| A10407 | Regular | 19 | 0 | 0 | 19 |
| A10407 | Silence | 19 | 1 | 1 | 19 |
| A10407 | Irregular | 18 | 0 | 1 | 20 |
| A10408 | Regular | 19 | 1 | 0 | 18 |
| A10408 | Silence | 18 | 0 | 1 | 20 |
| A10408 | Irregular | 20 | 0 | 0 | 20 |
| A10409 | Regular | 18 | 1 | 1 | 19 |
| A10409 | Silence | 15 | 1 | 4 | 18 |
| A10409 | Irregular | 15 | 1 | 5 | 19 |
| A10410 | Regular | 16 | 1 | 3 | 18 |
| A10410 | Silence | 18 | 2 | 2 | 18 |
| A10410 | Irregular | 19 | 1 | 0 | 19 |
| A10411 | Regular | 17 | 3 | 2 | 16 |
| A10411 | Silence | 16 | 1 | 3 | 19 |
| A10411 | Irregular | 17 | 2 | 3 | 18 |
| A10414 | Regular | 17 | 0 | 2 | 20 |
| A10414 | Silence | 19 | 0 | 0 | 19 |
| A10414 | Irregular | 18 | 2 | 2 | 18 |
| A10416 | Regular | 14 | 3 | 6 | 17 |
| A10416 | Silence | 15 | 0 | 4 | 20 |
| A10416 | Irregular | 16 | 2 | 3 | 17 |
| A10419 | Regular | 19 | 4 | 0 | 16 |
| A10419 | Silence | 12 | 2 | 8 | 18 |
| A10419 | Irregular | 14 | 0 | 5 | 19 |
| A10420 | Regular | 14 | 5 | 6 | 15 |
| A10420 | Silence | 11 | 2 | 8 | 18 |
| A10420 | Irregular | 14 | 1 | 5 | 18 |
| A10421 | Regular | 16 | 1 | 4 | 19 |
| A10421 | Silence | 15 | 1 | 4 | 18 |
| A10421 | Irregular | 14 | 2 | 5 | 18 |
| A10422 | Regular | 15 | 4 | 4 | 15 |
| A10422 | Silence | 15 | 4 | 5 | 16 |
| A10422 | Irregular | 11 | 5 | 8 | 15 |
| A10423 | Regular | 18 | 2 | 1 | 18 |
| A10423 | Silence | 17 | 2 | 3 | 18 |
| A10423 | Irregular | 19 | 1 | 0 | 18 |
| A10424 | Regular | 19 | 0 | 1 | 20 |
| A10424 | Silence | 18 | 1 | 1 | 19 |
| A10424 | Irregular | 18 | 1 | 1 | 18 |
| A10425 | Regular | 18 | 0 | 1 | 19 |
| A10425 | Silence | 18 | 1 | 1 | 19 |
| A10425 | Irregular | 17 | 2 | 3 | 18 |
| A10426 | Regular | 17 | 3 | 3 | 17 |
| A10426 | Silence | 18 | 3 | 1 | 17 |
| A10426 | Irregular | 19 | 1 | 0 | 18 |
| A10427 | Regular | 17 | 1 | 3 | 19 |
| A10427 | Silence | 14 | 3 | 5 | 16 |
| A10427 | Irregular | 13 | 2 | 6 | 18 |
| A10428 | Regular | 19 | 2 | 0 | 17 |
| A10428 | Silence | 18 | 4 | 2 | 16 |
| A10428 | Irregular | 19 | 0 | 0 | 20 |
| A10429 | Regular | 19 | 0 | 1 | 20 |
| A10429 | Silence | 18 | 0 | 1 | 20 |
| A10429 | Irregular | 17 | 0 | 2 | 19 |
| A10430 | Regular | 17 | 2 | 3 | 18 |
| A10430 | Silence | 15 | 1 | 4 | 18 |
| A10430 | Irregular | 18 | 0 | 1 | 20 |
| A10432 | Regular | 11 | 8 | 8 | 11 |
| A10432 | Silence | 10 | 6 | 9 | 14 |
| A10432 | Irregular | 9 | 5 | 11 | 15 |
| DNS214 | Regular | 15 | 12 | 4 | 8 |
| DNS214 | Silence | 17 | 9 | 2 | 10 |
| DNS214 | Irregular | 19 | 11 | 1 | 9 |
| Z10442 | Regular | 18 | 0 | 1 | 20 |
| Z10442 | Silence | 20 | 1 | 0 | 19 |
| Z10442 | Irregular | 17 | 0 | 2 | 19 |
| **Appendix 3**. Number hits, false alarms, misses and correct rejections per prime per participant in Experiment 1. | | | | | |

| 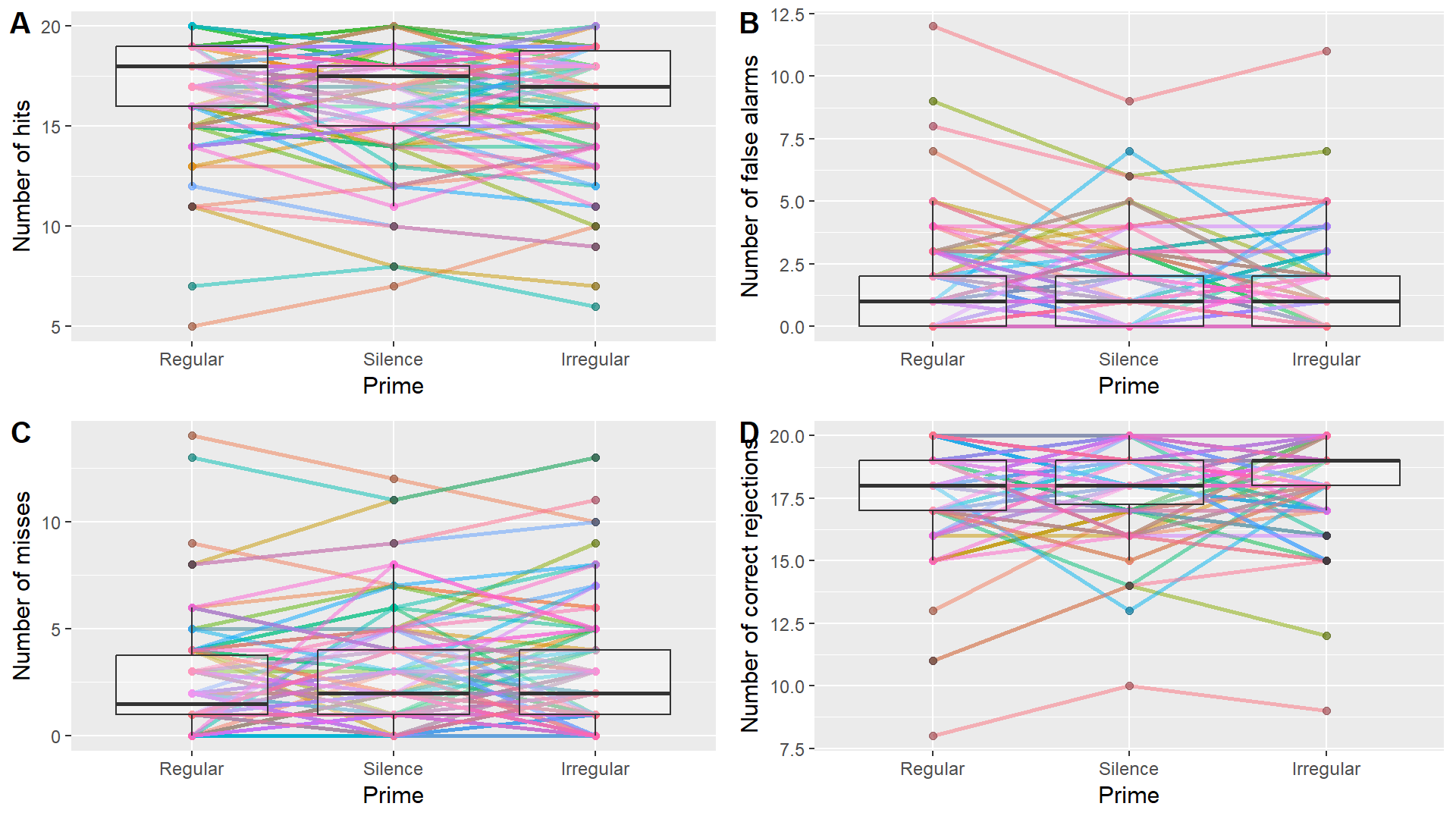 |
| --- |
| **Appendix 3b**: Boxplots of number hits (A), false alarms (B), misses (C) and correct rejections (D) by Prime in Experiment 1. The points represent individual participants. |

| Subject | Prime | nhits | nfalsealrams | nmiss | ncr |
| --- | --- | --- | --- | --- | --- |
| 10504 | Regular | 13 | 2 | 6 | 18 |
| 10504 | Silence | 17 | 2 | 3 | 18 |
| 10504 | Irregular | 11 | 0 | 9 | 20 |
| A10501 | Regular | 10 | 2 | 10 | 18 |
| A10501 | Silence | 7 | 4 | 12 | 16 |
| A10501 | Irregular | 9 | 6 | 11 | 14 |
| A10502 | Regular | 19 | 2 | 1 | 18 |
| A10502 | Silence | 19 | 2 | 1 | 18 |
| A10502 | Irregular | 16 | 1 | 3 | 19 |
| A10503 | Regular | 16 | 9 | 4 | 11 |
| A10503 | Silence | 13 | 9 | 7 | 11 |
| A10503 | Irregular | 10 | 14 | 9 | 6 |
| A10505 | Regular | 17 | 0 | 2 | 20 |
| A10505 | Silence | 19 | 0 | 1 | 20 |
| A10505 | Irregular | 18 | 1 | 2 | 19 |
| A10506 | Regular | 18 | 0 | 1 | 20 |
| A10506 | Silence | 18 | 0 | 2 | 20 |
| A10506 | Irregular | 20 | 0 | 0 | 20 |
| A10507 | Regular | 15 | 3 | 5 | 17 |
| A10507 | Silence | 16 | 2 | 4 | 18 |
| A10507 | Irregular | 18 | 1 | 1 | 19 |
| A10510 | Regular | 20 | 0 | 0 | 20 |
| A10510 | Silence | 18 | 2 | 1 | 18 |
| A10510 | Irregular | 18 | 2 | 2 | 18 |
| A10511 | Regular | 8 | 3 | 11 | 17 |
| A10511 | Silence | 9 | 4 | 11 | 16 |
| A10511 | Irregular | 6 | 8 | 14 | 12 |
| A10512 | Regular | 11 | 3 | 8 | 17 |
| A10512 | Silence | 15 | 2 | 5 | 18 |
| A10512 | Irregular | 15 | 2 | 5 | 18 |
| A10513 | Regular | 20 | 1 | 0 | 19 |
| A10513 | Silence | 17 | 3 | 2 | 17 |
| A10513 | Irregular | 16 | 1 | 4 | 19 |
| A10514 | Regular | 15 | 7 | 4 | 13 |
| A10514 | Silence | 17 | 2 | 3 | 18 |
| A10514 | Irregular | 15 | 3 | 5 | 17 |
| A10515 | Regular | 20 | 1 | 0 | 19 |
| A10515 | Silence | 19 | 0 | 1 | 20 |
| A10515 | Irregular | 18 | 1 | 1 | 19 |
| A10516 | Regular | 11 | 2 | 8 | 18 |
| A10516 | Silence | 10 | 4 | 10 | 16 |
| A10516 | Irregular | 15 | 3 | 5 | 17 |
| A10517 | Regular | 17 | 3 | 3 | 17 |
| A10517 | Silence | 11 | 2 | 8 | 18 |
| A10517 | Irregular | 16 | 1 | 4 | 19 |
| A10518 | Regular | 19 | 4 | 1 | 16 |
| A10518 | Silence | 13 | 2 | 6 | 18 |
| A10518 | Irregular | 14 | 4 | 6 | 16 |
| A10519 | Regular | 11 | 0 | 9 | 20 |
| A10519 | Silence | 12 | 3 | 8 | 17 |
| A10519 | Irregular | 8 | 2 | 11 | 18 |
| A10520 | Regular | 18 | 1 | 1 | 19 |
| A10520 | Silence | 18 | 0 | 2 | 20 |
| A10520 | Irregular | 16 | 2 | 4 | 18 |
| A10521 | Regular | 19 | 2 | 1 | 18 |
| A10521 | Silence | 18 | 3 | 2 | 17 |
| A10521 | Irregular | 14 | 0 | 5 | 20 |
| A10522 | Regular | 15 | 0 | 4 | 20 |
| A10522 | Silence | 19 | 1 | 1 | 19 |
| A10522 | Irregular | 19 | 4 | 1 | 16 |
| A10523 | Regular | 18 | 2 | 2 | 18 |
| A10523 | Silence | 17 | 0 | 2 | 20 |
| A10523 | Irregular | 17 | 1 | 3 | 19 |
| A10525 | Regular | 14 | 1 | 6 | 19 |
| A10525 | Silence | 14 | 3 | 6 | 17 |
| A10525 | Irregular | 15 | 0 | 4 | 20 |
| a10527 | Regular | 17 | 4 | 3 | 16 |
| a10527 | Silence | 14 | 5 | 5 | 15 |
| a10527 | Irregular | 14 | 5 | 6 | 15 |
| A10527 | Regular | 12 | 5 | 8 | 15 |
| A10527 | Silence | 19 | 5 | 1 | 15 |
| A10527 | Irregular | 12 | 4 | 7 | 16 |
| A10528 | Regular | 19 | 0 | 1 | 20 |
| A10528 | Silence | 18 | 4 | 1 | 16 |
| A10528 | Irregular | 20 | 1 | 0 | 19 |
| A10530 | Regular | 18 | 1 | 1 | 19 |
| A10530 | Silence | 18 | 2 | 2 | 18 |
| A10530 | Irregular | 20 | 2 | 0 | 18 |
| A10531 | Regular | 13 | 5 | 7 | 15 |
| A10531 | Silence | 13 | 3 | 7 | 17 |
| A10531 | Irregular | 9 | 7 | 10 | 13 |
| A10532 | Regular | 20 | 1 | 0 | 19 |
| A10532 | Silence | 19 | 0 | 1 | 20 |
| A10532 | Irregular | 18 | 3 | 1 | 17 |
| A10533 | Regular | 19 | 5 | 1 | 15 |
| A10533 | Silence | 17 | 5 | 3 | 15 |
| A10533 | Irregular | 19 | 3 | 0 | 17 |
| A10534 | Regular | 20 | 5 | 0 | 15 |
| A10534 | Silence | 17 | 2 | 2 | 18 |
| A10534 | Irregular | 18 | 3 | 2 | 17 |
| A10535 | Regular | 17 | 1 | 2 | 19 |
| A10535 | Silence | 15 | 1 | 5 | 19 |
| A10535 | Irregular | 12 | 4 | 8 | 16 |
| A10537 | Regular | 19 | 0 | 1 | 20 |
| A10537 | Silence | 18 | 0 | 1 | 20 |
| A10537 | Irregular | 17 | 2 | 3 | 18 |
| A10538 | Regular | 14 | 3 | 6 | 17 |
| A10538 | Silence | 15 | 3 | 4 | 17 |
| A10538 | Irregular | 14 | 4 | 6 | 16 |
| A10539 | Regular | 14 | 1 | 5 | 19 |
| A10539 | Silence | 18 | 2 | 2 | 18 |
| A10539 | Irregular | 17 | 0 | 3 | 20 |
| A10542 | Regular | 17 | 0 | 2 | 20 |
| A10542 | Silence | 19 | 0 | 1 | 20 |
| A10542 | Irregular | 16 | 1 | 4 | 19 |
| A10543 | Regular | 14 | 2 | 6 | 18 |
| A10543 | Silence | 15 | 4 | 4 | 16 |
| A10543 | Irregular | 15 | 3 | 5 | 17 |
| A10544 | Regular | 19 | 0 | 0 | 20 |
| A10544 | Silence | 20 | 0 | 0 | 20 |
| A10544 | Irregular | 18 | 0 | 2 | 20 |
| A10545 | Regular | 13 | 2 | 6 | 18 |
| A10545 | Silence | 12 | 1 | 8 | 19 |
| A10545 | Irregular | 14 | 1 | 6 | 19 |
| A10546 | Regular | 15 | 3 | 5 | 17 |
| A10546 | Silence | 9 | 3 | 11 | 17 |
| A10546 | Irregular | 14 | 6 | 5 | 14 |
| A10547 | Regular | 13 | 2 | 6 | 18 |
| A10547 | Silence | 17 | 2 | 3 | 18 |
| A10547 | Irregular | 16 | 0 | 4 | 20 |
| A10548 | Regular | 13 | 0 | 7 | 20 |
| A10548 | Silence | 16 | 2 | 4 | 18 |
| A10548 | Irregular | 16 | 2 | 3 | 18 |
| A10549 | Regular | 17 | 3 | 2 | 17 |
| A10549 | Silence | 18 | 2 | 2 | 18 |
| A10549 | Irregular | 17 | 3 | 3 | 17 |
| A10550 | Regular | 12 | 0 | 8 | 20 |
| A10550 | Silence | 13 | 2 | 6 | 18 |
| A10550 | Irregular | 12 | 1 | 8 | 19 |
| A10551 | Regular | 8 | 8 | 12 | 12 |
| A10551 | Silence | 10 | 3 | 10 | 17 |
| A10551 | Irregular | 8 | 5 | 11 | 15 |
| A10552 | Regular | 10 | 3 | 10 | 17 |
| A10552 | Silence | 8 | 5 | 12 | 15 |
| A10552 | Irregular | 5 | 6 | 14 | 14 |
| A10553 | Regular | 13 | 3 | 7 | 17 |
| A10553 | Silence | 12 | 2 | 8 | 18 |
| A10553 | Irregular | 9 | 2 | 10 | 18 |
| A10554 | Regular | 14 | 3 | 6 | 17 |
| A10554 | Silence | 18 | 1 | 2 | 19 |
| A10554 | Irregular | 17 | 0 | 2 | 20 |
| A10555 | Regular | 17 | 3 | 2 | 17 |
| A10555 | Silence | 17 | 6 | 3 | 14 |
| A10555 | Irregular | 17 | 3 | 3 | 17 |
| A10556 | Regular | 6 | 5 | 13 | 15 |
| A10556 | Silence | 11 | 7 | 9 | 13 |
| A10556 | Irregular | 11 | 6 | 9 | 14 |
| A10557 | Regular | 12 | 4 | 8 | 16 |
| A10557 | Silence | 13 | 0 | 6 | 20 |
| A10557 | Irregular | 15 | 0 | 5 | 20 |
| A10558 | Regular | 20 | 1 | 0 | 19 |
| A10558 | Silence | 19 | 1 | 0 | 19 |
| A10558 | Irregular | 18 | 1 | 2 | 19 |
| A10559 | Regular | 15 | 3 | 4 | 17 |
| A10559 | Silence | 16 | 1 | 4 | 19 |
| A10559 | Irregular | 17 | 1 | 3 | 19 |
| A10560 | Regular | 17 | 2 | 3 | 18 |
| A10560 | Silence | 16 | 3 | 4 | 17 |
| A10560 | Irregular | 10 | 3 | 9 | 17 |
| A10561 | Regular | 17 | 2 | 2 | 18 |
| A10561 | Silence | 15 | 4 | 5 | 16 |
| A10561 | Irregular | 18 | 2 | 2 | 18 |
| A10562 | Regular | 15 | 0 | 5 | 20 |
| A10562 | Silence | 14 | 1 | 6 | 19 |
| A10562 | Irregular | 15 | 0 | 4 | 20 |
| A10564 | Regular | 18 | 0 | 1 | 20 |
| A10564 | Silence | 18 | 0 | 2 | 20 |
| A10564 | Irregular | 20 | 0 | 0 | 20 |
| A10565 | Regular | 19 | 1 | 1 | 19 |
| A10565 | Silence | 16 | 3 | 3 | 17 |
| A10565 | Irregular | 17 | 0 | 3 | 20 |
| A10566 | Regular | 16 | 5 | 4 | 15 |
| A10566 | Silence | 16 | 1 | 4 | 19 |
| A10566 | Irregular | 14 | 0 | 5 | 20 |
| A10567 | Regular | 19 | 1 | 1 | 19 |
| A10567 | Silence | 17 | 1 | 3 | 19 |
| A10567 | Irregular | 15 | 0 | 4 | 20 |
| A10568 | Regular | 16 | 3 | 4 | 17 |
| A10568 | Silence | 15 | 3 | 4 | 17 |
| A10568 | Irregular | 18 | 3 | 2 | 17 |
| A10569 | Regular | 15 | 1 | 4 | 19 |
| A10569 | Silence | 19 | 0 | 1 | 20 |
| A10569 | Irregular | 14 | 1 | 6 | 19 |
| A10570 | Regular | 18 | 0 | 2 | 20 |
| A10570 | Silence | 19 | 1 | 1 | 19 |
| A10570 | Irregular | 18 | 2 | 1 | 18 |
| A10571 | Regular | 14 | 1 | 6 | 19 |
| A10571 | Silence | 15 | 0 | 4 | 20 |
| A10571 | Irregular | 16 | 1 | 4 | 19 |
| A10572 | Regular | 18 | 1 | 2 | 19 |
| A10572 | Silence | 18 | 2 | 1 | 18 |
| A10572 | Irregular | 19 | 2 | 1 | 18 |
| A10573 | Regular | 14 | 3 | 5 | 17 |
| A10573 | Silence | 17 | 0 | 3 | 20 |
| A10573 | Irregular | 15 | 0 | 5 | 20 |
| A10574 | Regular | 15 | 0 | 4 | 20 |
| A10574 | Silence | 18 | 2 | 2 | 18 |
| A10574 | Irregular | 18 | 2 | 2 | 18 |
| A10575 | Regular | 17 | 4 | 2 | 16 |
| A10575 | Silence | 12 | 1 | 8 | 19 |
| A10575 | Irregular | 15 | 2 | 5 | 18 |
| A10576 | Regular | 15 | 2 | 5 | 18 |
| A10576 | Silence | 19 | 1 | 0 | 19 |
| A10576 | Irregular | 20 | 3 | 0 | 17 |
| A10578 | Regular | 17 | 0 | 3 | 20 |
| A10578 | Silence | 18 | 2 | 2 | 18 |
| A10578 | Irregular | 14 | 1 | 5 | 19 |
| A10579 | Regular | 20 | 0 | 0 | 20 |
| A10579 | Silence | 16 | 1 | 3 | 19 |
| A10579 | Irregular | 16 | 1 | 4 | 19 |
| A10580 | Regular | 18 | 1 | 2 | 19 |
| A10580 | Silence | 17 | 1 | 2 | 19 |
| A10580 | Irregular | 19 | 0 | 1 | 20 |
| A10581 | Regular | 20 | 1 | 0 | 19 |
| A10581 | Silence | 19 | 0 | 0 | 20 |
| A10581 | Irregular | 20 | 0 | 0 | 20 |
| A10582 | Regular | 9 | 6 | 11 | 14 |
| A10582 | Silence | 13 | 2 | 6 | 18 |
| A10582 | Irregular | 11 | 3 | 9 | 17 |
| A10584 | Regular | 20 | 0 | 0 | 20 |
| A10584 | Silence | 20 | 0 | 0 | 20 |
| A10584 | Irregular | 19 | 0 | 0 | 20 |
| A10585 | Regular | 17 | 1 | 2 | 19 |
| A10585 | Silence | 18 | 1 | 2 | 19 |
| A10585 | Irregular | 18 | 1 | 2 | 19 |
| A10586 | Regular | 19 | 1 | 1 | 19 |
| A10586 | Silence | 19 | 0 | 1 | 20 |
| A10586 | Irregular | 19 | 1 | 0 | 19 |
| A10587 | Regular | 16 | 1 | 3 | 19 |
| A10587 | Silence | 11 | 4 | 9 | 16 |
| A10587 | Irregular | 15 | 2 | 5 | 18 |
| A10588 | Regular | 17 | 1 | 3 | 19 |
| A10588 | Silence | 19 | 4 | 1 | 16 |
| A10588 | Irregular | 19 | 9 | 0 | 11 |
| A10589 | Regular | 16 | 0 | 4 | 20 |
| A10589 | Silence | 15 | 1 | 4 | 19 |
| A10589 | Irregular | 13 | 2 | 7 | 18 |
| A10590 | Regular | 19 | 1 | 1 | 19 |
| A10590 | Silence | 18 | 1 | 2 | 19 |
| A10590 | Irregular | 19 | 0 | 0 | 20 |
| A10591 | Regular | 12 | 2 | 8 | 18 |
| A10591 | Silence | 13 | 1 | 6 | 19 |
| A10591 | Irregular | 16 | 1 | 4 | 19 |
| A10592 | Regular | 16 | 5 | 4 | 15 |
| A10592 | Silence | 13 | 4 | 6 | 16 |
| A10592 | Irregular | 17 | 4 | 3 | 16 |
| A10594 | Regular | 18 | 5 | 2 | 15 |
| A10594 | Silence | 14 | 0 | 5 | 20 |
| A10594 | Irregular | 20 | 0 | 0 | 20 |
| A10595 | Regular | 18 | 0 | 2 | 20 |
| A10595 | Silence | 16 | 1 | 3 | 19 |
| A10595 | Irregular | 17 | 0 | 3 | 20 |
| A10597 | Regular | 16 | 2 | 4 | 18 |
| A10597 | Silence | 14 | 2 | 6 | 18 |
| A10597 | Irregular | 13 | 1 | 6 | 19 |
| A10598 | Regular | 14 | 3 | 6 | 17 |
| A10598 | Silence | 14 | 2 | 5 | 18 |
| A10598 | Irregular | 9 | 3 | 11 | 17 |
| A10599 | Regular | 18 | 0 | 1 | 20 |
| A10599 | Silence | 18 | 0 | 2 | 20 |
| A10599 | Irregular | 18 | 0 | 2 | 20 |
| A10600 | Regular | 19 | 1 | 1 | 19 |
| A10600 | Silence | 17 | 2 | 3 | 18 |
| A10600 | Irregular | 16 | 1 | 3 | 19 |
| X10630 | Regular | 17 | 3 | 3 | 17 |
| X10630 | Silence | 19 | 2 | 1 | 18 |
| X10630 | Irregular | 16 | 8 | 3 | 12 |
| **Appendix 4**. Number hits, false alarms, misses and correct rejections per prime per participant in Experiment 2. | | | | | |

| 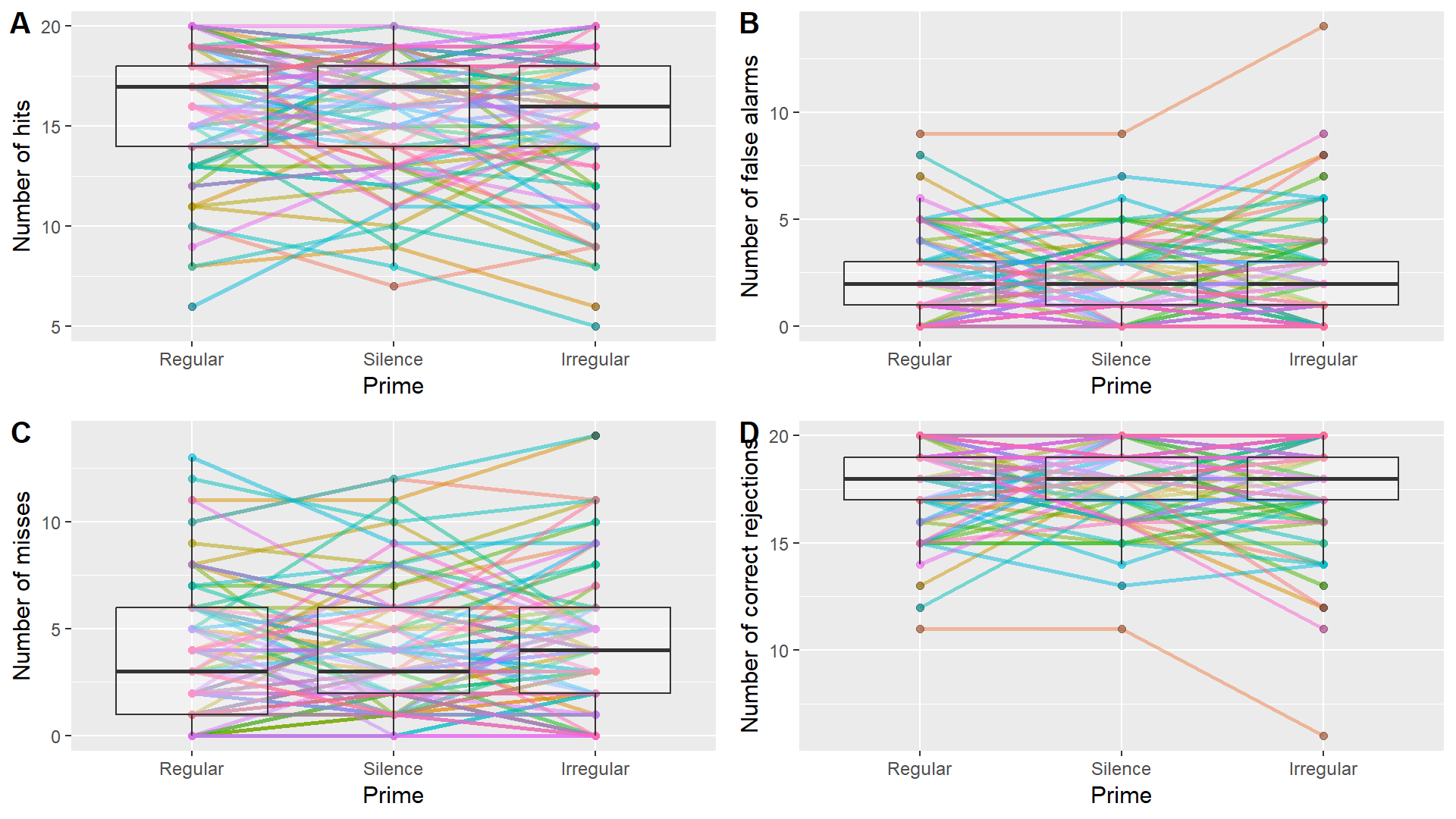 |
| --- |
| **Appendix 4b**: Boxplots of number hits (A), false alarms (B), misses (C) and correct rejections (D) by Prime in Experiment 2. The points represent individual participants. |

| Subject | Prime | nhits | nfalsealrams | nmiss | ncr |
| --- | --- | --- | --- | --- | --- |
| 3302 | Regular | 20 | 0 | 0 | 20 |
| 3302 | Silence | 18 | 1 | 2 | 19 |
| 3302 | Irregular | 18 | 0 | 1 | 20 |
| 3304 | Regular | 11 | 6 | 9 | 14 |
| 3304 | Silence | 12 | 6 | 8 | 14 |
| 3304 | Irregular | 15 | 2 | 4 | 18 |
| 3308 | Regular | 19 | 0 | 0 | 20 |
| 3308 | Silence | 19 | 0 | 1 | 20 |
| 3308 | Irregular | 18 | 0 | 2 | 20 |
| 3310 | Regular | 17 | 4 | 3 | 16 |
| 3310 | Silence | 18 | 1 | 1 | 19 |
| 3310 | Irregular | 19 | 1 | 1 | 19 |
| 3314 | Regular | 18 | 1 | 1 | 19 |
| 3314 | Silence | 19 | 1 | 1 | 19 |
| 3314 | Irregular | 19 | 1 | 1 | 19 |
| 3315 | Regular | 15 | 5 | 4 | 15 |
| 3315 | Silence | 18 | 5 | 2 | 15 |
| 3315 | Irregular | 16 | 1 | 4 | 19 |
| 3316 | Regular | 20 | 1 | 0 | 19 |
| 3316 | Silence | 18 | 1 | 1 | 19 |
| 3316 | Irregular | 20 | 1 | 0 | 19 |
| 3318 | Regular | 18 | 0 | 1 | 20 |
| 3318 | Silence | 17 | 3 | 3 | 17 |
| 3318 | Irregular | 19 | 2 | 1 | 18 |
| 3320 | Regular | 20 | 0 | 0 | 20 |
| 3320 | Silence | 19 | 2 | 1 | 18 |
| 3320 | Irregular | 17 | 0 | 2 | 20 |
| 3321 | Regular | 17 | 1 | 2 | 19 |
| 3321 | Silence | 19 | 1 | 1 | 19 |
| 3321 | Irregular | 18 | 0 | 2 | 20 |
| 3322 | Regular | 15 | 3 | 5 | 17 |
| 3322 | Silence | 13 | 3 | 7 | 17 |
| 3322 | Irregular | 17 | 2 | 2 | 18 |
| 3323 | Regular | 15 | 2 | 5 | 18 |
| 3323 | Silence | 15 | 3 | 4 | 17 |
| 3323 | Irregular | 17 | 3 | 3 | 17 |
| 3324 | Regular | 17 | 0 | 3 | 20 |
| 3324 | Silence | 19 | 1 | 1 | 19 |
| 3324 | Irregular | 17 | 1 | 2 | 19 |
| 3327 | Regular | 19 | 1 | 1 | 19 |
| 3327 | Silence | 19 | 0 | 0 | 20 |
| 3327 | Irregular | 17 | 2 | 3 | 18 |
| 3329 | Regular | 19 | 0 | 1 | 20 |
| 3329 | Silence | 19 | 0 | 0 | 20 |
| 3329 | Irregular | 18 | 1 | 2 | 19 |
| 3330 | Regular | 18 | 0 | 2 | 20 |
| 3330 | Silence | 19 | 0 | 0 | 20 |
| 3330 | Irregular | 18 | 0 | 2 | 20 |
| 3332 | Regular | 20 | 0 | 0 | 20 |
| 3332 | Silence | 19 | 0 | 1 | 20 |
| 3332 | Irregular | 18 | 2 | 1 | 18 |
| 3333 | Regular | 18 | 1 | 2 | 19 |
| 3333 | Silence | 17 | 1 | 2 | 19 |
| 3333 | Irregular | 17 | 0 | 3 | 20 |
| 3334 | Regular | 15 | 0 | 5 | 20 |
| 3334 | Silence | 14 | 0 | 6 | 20 |
| 3334 | Irregular | 14 | 0 | 5 | 20 |
| 3335 | Regular | 18 | 0 | 2 | 20 |
| 3335 | Silence | 19 | 0 | 1 | 20 |
| 3335 | Irregular | 14 | 1 | 5 | 19 |
| 3336 | Regular | 4 | 4 | 15 | 16 |
| 3336 | Silence | 5 | 4 | 15 | 16 |
| 3336 | Irregular | 4 | 4 | 16 | 16 |
| 3339 | Regular | 17 | 2 | 3 | 18 |
| 3339 | Silence | 18 | 3 | 2 | 17 |
| 3339 | Irregular | 17 | 1 | 2 | 19 |
| 3341 | Regular | 18 | 2 | 1 | 18 |
| 3341 | Silence | 19 | 2 | 1 | 18 |
| 3341 | Irregular | 20 | 3 | 0 | 17 |
| 3342 | Regular | 15 | 4 | 5 | 16 |
| 3342 | Silence | 10 | 4 | 9 | 16 |
| 3342 | Irregular | 15 | 3 | 5 | 17 |
| 3343 | Regular | 17 | 6 | 3 | 14 |
| 3343 | Silence | 12 | 3 | 7 | 17 |
| 3343 | Irregular | 19 | 1 | 1 | 19 |
| 3344 | Regular | 18 | 2 | 1 | 18 |
| 3344 | Silence | 18 | 3 | 2 | 17 |
| 3344 | Irregular | 17 | 0 | 3 | 20 |
| 3346 | Regular | 18 | 0 | 2 | 20 |
| 3346 | Silence | 17 | 0 | 2 | 20 |
| 3346 | Irregular | 16 | 0 | 4 | 20 |
| 3347 | Regular | 20 | 3 | 0 | 17 |
| 3347 | Silence | 18 | 2 | 1 | 18 |
| 3347 | Irregular | 19 | 0 | 1 | 20 |
| 3348 | Regular | 19 | 0 | 1 | 20 |
| 3348 | Silence | 18 | 0 | 1 | 20 |
| 3348 | Irregular | 19 | 1 | 1 | 19 |
| 3349 | Regular | 17 | 0 | 3 | 20 |
| 3349 | Silence | 18 | 1 | 2 | 19 |
| 3349 | Irregular | 17 | 0 | 2 | 20 |
| 3350 | Regular | 20 | 1 | 0 | 19 |
| 3350 | Silence | 19 | 0 | 0 | 20 |
| 3350 | Irregular | 20 | 0 | 0 | 20 |
| 3351 | Regular | 19 | 0 | 0 | 20 |
| 3351 | Silence | 19 | 0 | 1 | 20 |
| 3351 | Irregular | 19 | 0 | 1 | 20 |
| 3353 | Regular | 20 | 2 | 0 | 18 |
| 3353 | Silence | 19 | 2 | 1 | 18 |
| 3353 | Irregular | 19 | 1 | 0 | 19 |
| 3358 | Regular | 19 | 0 | 1 | 20 |
| 3358 | Silence | 20 | 0 | 0 | 20 |
| 3358 | Irregular | 19 | 0 | 0 | 20 |
| 3359 | Regular | 20 | 0 | 0 | 20 |
| 3359 | Silence | 19 | 0 | 0 | 20 |
| 3359 | Irregular | 20 | 0 | 0 | 20 |
| 3360 | Regular | 19 | 3 | 0 | 17 |
| 3360 | Silence | 19 | 3 | 1 | 17 |
| 3360 | Irregular | 20 | 1 | 0 | 19 |
| 3361 | Regular | 16 | 5 | 3 | 15 |
| 3361 | Silence | 18 | 4 | 2 | 16 |
| 3361 | Irregular | 14 | 3 | 6 | 17 |
| 3365 | Regular | 16 | 0 | 3 | 20 |
| 3365 | Silence | 18 | 0 | 2 | 20 |
| 3365 | Irregular | 16 | 2 | 4 | 18 |
| 3366 | Regular | 17 | 1 | 3 | 19 |
| 3366 | Silence | 18 | 1 | 1 | 19 |
| 3366 | Irregular | 17 | 0 | 3 | 20 |
| 3367 | Regular | 18 | 3 | 1 | 17 |
| 3367 | Silence | 18 | 2 | 2 | 18 |
| 3367 | Irregular | 18 | 1 | 2 | 19 |
| 3369 | Regular | 18 | 2 | 1 | 18 |
| 3369 | Silence | 20 | 2 | 0 | 18 |
| 3369 | Irregular | 17 | 1 | 3 | 19 |
| 3370 | Regular | 17 | 1 | 3 | 19 |
| 3370 | Silence | 18 | 0 | 2 | 20 |
| 3370 | Irregular | 18 | 0 | 1 | 20 |
| 3371 | Regular | 18 | 1 | 2 | 19 |
| 3371 | Silence | 18 | 1 | 2 | 19 |
| 3371 | Irregular | 19 | 1 | 0 | 19 |
| 3372 | Regular | 18 | 4 | 2 | 16 |
| 3372 | Silence | 17 | 2 | 3 | 18 |
| 3372 | Irregular | 14 | 2 | 5 | 18 |
| 3375 | Regular | 19 | 2 | 0 | 18 |
| 3375 | Silence | 17 | 1 | 3 | 19 |
| 3375 | Irregular | 18 | 2 | 2 | 18 |
| 3377 | Regular | 16 | 0 | 4 | 20 |
| 3377 | Silence | 18 | 0 | 2 | 20 |
| 3377 | Irregular | 16 | 2 | 3 | 18 |
| 3379 | Regular | 17 | 1 | 2 | 19 |
| 3379 | Silence | 19 | 1 | 1 | 19 |
| 3379 | Irregular | 19 | 2 | 1 | 18 |
| 3381 | Regular | 18 | 3 | 1 | 17 |
| 3381 | Silence | 20 | 2 | 0 | 18 |
| 3381 | Irregular | 19 | 1 | 1 | 19 |
| 3382 | Regular | 20 | 0 | 0 | 20 |
| 3382 | Silence | 19 | 0 | 1 | 20 |
| 3382 | Irregular | 18 | 1 | 1 | 19 |
| 3383 | Regular | 15 | 2 | 5 | 18 |
| 3383 | Silence | 15 | 2 | 5 | 18 |
| 3383 | Irregular | 15 | 2 | 4 | 18 |
| 3384 | Regular | 17 | 2 | 3 | 18 |
| 3384 | Silence | 16 | 1 | 3 | 19 |
| 3384 | Irregular | 14 | 1 | 6 | 19 |
| 3387 | Regular | 18 | 0 | 1 | 20 |
| 3387 | Silence | 18 | 1 | 2 | 19 |
| 3387 | Irregular | 20 | 2 | 0 | 18 |
| 3388 | Regular | 18 | 2 | 2 | 18 |
| 3388 | Silence | 16 | 3 | 3 | 17 |
| 3388 | Irregular | 18 | 0 | 2 | 20 |
| 3390 | Regular | 16 | 2 | 4 | 18 |
| 3390 | Silence | 20 | 3 | 0 | 17 |
| 3390 | Irregular | 17 | 0 | 2 | 20 |
| 3391 | Regular | 19 | 2 | 0 | 18 |
| 3391 | Silence | 17 | 2 | 3 | 18 |
| 3391 | Irregular | 14 | 3 | 6 | 17 |
| 3393 | Regular | 19 | 2 | 1 | 18 |
| 3393 | Silence | 20 | 1 | 0 | 19 |
| 3393 | Irregular | 17 | 1 | 2 | 19 |
| 3396 | Regular | 20 | 0 | 0 | 20 |
| 3396 | Silence | 19 | 0 | 0 | 20 |
| 3396 | Irregular | 20 | 0 | 0 | 20 |
| 3398 | Regular | 12 | 0 | 8 | 20 |
| 3398 | Silence | 16 | 4 | 4 | 16 |
| 3398 | Irregular | 12 | 2 | 7 | 18 |
| 3399 | Regular | 17 | 0 | 3 | 20 |
| 3399 | Silence | 14 | 1 | 5 | 19 |
| 3399 | Irregular | 15 | 0 | 5 | 20 |
| 3402 | Regular | 15 | 3 | 4 | 17 |
| 3402 | Silence | 15 | 3 | 5 | 17 |
| 3402 | Irregular | 18 | 3 | 2 | 17 |
| **Appendix 5**. Number hits, false alarms, misses and correct rejections per prime per participant in Experiment 3. | | | | | |

| 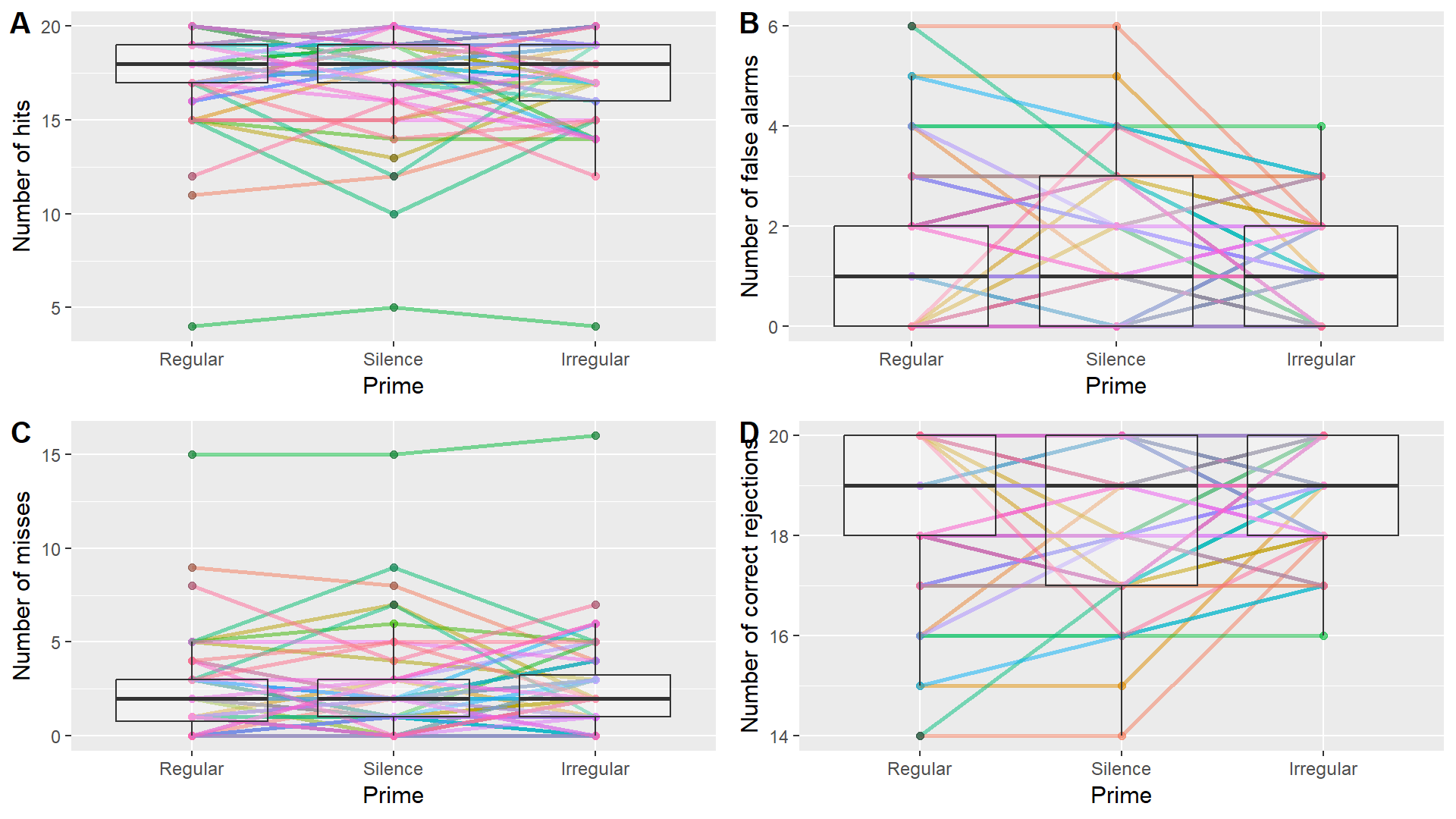 |
| --- |
| **Appendix 5b**: Boxplots of number hits (A), false alarms (B), misses (C) and correct rejections (D) by Prime in Experiment 3. The points represent individual participants. |
