# Supplementary material for: Not primed to agree? Short or no effect of rhythmic priming on typical adults processing number agreement
Source: Front Psychol. 2025 Jun 13;16:1512267. doi: 10.3389/fpsyg.2025.1512267 (PMC12204084; doi:10.3389/fpsyg.2025.1512267)
Supplement: Supplementary file 2 [file Table_1.docx]

| \|  \| **dprime** \| \| \| \| --- \| --- \| --- \| --- \| \| *Predictors* \| *Estimates* \| *CI* \| *p* \| \| (Intercept) \| 2.64 \| 2.48 – 2.81 \| **<0.001** \| \| Prime [Silence] \| -0.06 \| -0.20 – 0.07 \| 0.353 \| \| Prime [Irregular] \| -0.01 \| -0.14 – 0.13 \| 0.908 \| \| **Random Effects** \| \| \| \| \| σ^2^ \| 0.26 \| \| \| \| τ_00_ _Subject_ \| 0.49 \| \| \| \| ICC \| 0.65 \| \| \| \| N _Subject_ \| 109 \| \| \| \| Observations \| 327 \| \| \| \| Marginal R^2^ / Conditional R^2^ \| 0.001 / 0.650 \| \| \| |
| --- | --- | --- | --- | --- | --- | --- | --- | --- | --- | --- | --- | --- | --- | --- | --- | --- | --- | --- | --- | --- | --- | --- | --- | --- | --- | --- | --- | --- | --- | --- | --- | --- | --- | --- | --- | --- | --- | --- | --- | --- | --- | --- | --- | --- | --- | --- | --- | --- |
|  |

**Table 3: Summary of fixed effects obtained using the summary(model) function of the lme4 package in R. Model: D' ~ Prime + 1|Participant on data from Experiment 1**
