# Supplementary material for: Not primed to agree? Short or no effect of rhythmic priming on typical adults processing number agreement
Source: Front Psychol. 2025 Jun 13;16:1512267. doi: 10.3389/fpsyg.2025.1512267 (PMC12204084; doi:10.3389/fpsyg.2025.1512267)
Supplement: Supplementary file 4 [file Table_3.docx]

| \|  \| **dprime** \| \| \| \| --- \| --- \| --- \| --- \| \| *Predictors* \| *Estimates* \| *CI* \| *p* \| \| (Intercept) \| 2.50 \| 2.35 – 2.65 \| **<0.001** \| \| Prime [Silence] \| -0.08 \| -0.22 – 0.06 \| 0.244 \| \| Prime [Irregular] \| -0.06 \| -0.20 – 0.08 \| 0.372 \| \| Miniblockhalf [Second] \| -0.13 \| -0.27 – 0.01 \| 0.063 \| \| Prime [Silence] × Miniblockhalf [Second] \| 0.05 \| -0.15 – 0.25 \| 0.613 \| \| Prime [Irregular] × Miniblockhalf [Second] \| 0.07 \| -0.12 – 0.27 \| 0.463 \| \| **Random Effects** \| \| \| \| \| σ^2^ \| 0.27 \| \| \| \| τ_00_ _Subject_ \| 0.35 \| \| \| \| ICC \| 0.56 \| \| \| \| N _Subject_ \| 109 \| \| \| \| Observations \| 654 \| \| \| \| Marginal R^2^ / Conditional R^2^ \| 0.005 / 0.566 \| \| \| |
| --- | --- | --- | --- | --- | --- | --- | --- | --- | --- | --- | --- | --- | --- | --- | --- | --- | --- | --- | --- | --- | --- | --- | --- | --- | --- | --- | --- | --- | --- | --- | --- | --- | --- | --- | --- | --- | --- | --- | --- | --- | --- | --- | --- | --- | --- | --- | --- | --- | --- | --- | --- | --- | --- | --- | --- | --- | --- | --- | --- | --- |
| **Table 5:** **Summary of fixed effects obtained using the summary(model) function of the lme4 package in R. Model: D' ~ Prime * Miniblockhalf + 1\|Participant on data from Experiment 1** |
