# Supplementary material for: Not primed to agree? Short or no effect of rhythmic priming on typical adults processing number agreement
Source: Front Psychol. 2025 Jun 13;16:1512267. doi: 10.3389/fpsyg.2025.1512267 (PMC12204084; doi:10.3389/fpsyg.2025.1512267)
Supplement: Supplementary file 5 [file Table_4.docx]

| \|  \| **Sum Sq** \| **Mean Sq** \| **NumDf** \| **DenDF** \| **F value** \| **Pr(>F)** \| \| --- \| --- \| --- \| --- \| --- \| --- \| --- \| \| Prime \| 0.3590235 \| 0.1795117 \| 2 \| 540 \| 0.6559423 \| 0.5193658 \| \| Miniblockhalf \| 1.3426823 \| 1.3426823 \| 1 \| 540 \| 4.9062092 \| 0.0271769 \| \| Prime:Miniblockhalf \| 0.1546596 \| 0.0773298 \| 2 \| 540 \| 0.2825659 \| 0.7539584 \| |
| --- | --- | --- | --- | --- | --- | --- | --- | --- | --- | --- | --- | --- | --- | --- | --- | --- | --- | --- | --- | --- | --- | --- | --- | --- | --- | --- | --- | --- |
| **Table 6:** **Main effects and interactions obtained using the anova(model) function in R.**  **Model: D' ~ Prime * Miniblockhalf + 1\|Participant on data from Experiment 1** |
