# Supplementary material for: Not primed to agree? Short or no effect of rhythmic priming on typical adults processing number agreement
Source: Front Psychol. 2025 Jun 13;16:1512267. doi: 10.3389/fpsyg.2025.1512267 (PMC12204084; doi:10.3389/fpsyg.2025.1512267)
Supplement: Supplementary file 6 [file Table_5.docx]

| \|  \| **dprime** \| \| \| \| --- \| --- \| --- \| --- \| \| *Predictors* \| *Estimates* \| *CI* \| *p* \| \| (Intercept) \| 1.94 \| 1.81 – 2.07 \| **<0.001** \| \| Prime [Silence] \| -0.19 \| -0.34 – -0.03 \| **0.016** \| \| Prime [Irregular] \| -0.14 \| -0.29 – 0.01 \| 0.076 \| \| **Random Effects** \| \| \| \| \| σ^2^ \| 0.33 \| \| \| \| τ_00_ _Subject_ \| 0.13 \| \| \| \| ICC \| 0.28 \| \| \| \| N _Subject_ \| 109 \| \| \| \| Observations \| 327 \| \| \| \| Marginal R^2^ / Conditional R^2^ \| 0.014 / 0.287 \| \| \| |
| --- | --- | --- | --- | --- | --- | --- | --- | --- | --- | --- | --- | --- | --- | --- | --- | --- | --- | --- | --- | --- | --- | --- | --- | --- | --- | --- | --- | --- | --- | --- | --- | --- | --- | --- | --- | --- | --- | --- | --- | --- | --- | --- | --- | --- | --- | --- | --- | --- |
| **Table 7:** **Summary of fixed effects obtained using the summary(model) function of the lme4 package in R. Model: D' ~ Prime + 1\|Participant on the first sentence after each prime in Experiment 1** |
