# Supplementary material for: Not primed to agree? Short or no effect of rhythmic priming on typical adults processing number agreement
Source: Front Psychol. 2025 Jun 13;16:1512267. doi: 10.3389/fpsyg.2025.1512267 (PMC12204084; doi:10.3389/fpsyg.2025.1512267)
Supplement: Supplementary file 9 [file Table_8.docx]

| \|  \| **Sum Sq** \| **Mean Sq** \| **NumDf** \| **DenDF** \| **F value** \| **Pr(>F)** \| \| --- \| --- \| --- \| --- \| --- \| --- \| --- \| \| Prime \| 0.2755115 \| 0.1377557 \| 2 \| 176 \| 0.526595 \| 0.5915402 \| |
| --- | --- | --- | --- | --- | --- | --- | --- | --- | --- | --- | --- | --- | --- | --- |
| **Table 10:** **Main effects and interactions obtained using the anova(model) function in R.**  **Model: D' ~ Prime + 1\|Participant on data from Experiment 2** |
