# Supplementary material for: Not primed to agree? Short or no effect of rhythmic priming on typical adults processing number agreement
Source: Front Psychol. 2025 Jun 13;16:1512267. doi: 10.3389/fpsyg.2025.1512267 (PMC12204084; doi:10.3389/fpsyg.2025.1512267)
Supplement: Supplementary file 10 [file Table_9.docx]

| \|  \| **dprime** \| \| \| \| --- \| --- \| --- \| --- \| \| *Predictors* \| *Estimates* \| *CI* \| *p* \| \| (Intercept) \| 2.09 \| 1.91 – 2.28 \| **<0.001** \| \| Prime [Silence] \| -0.06 \| -0.23 – 0.11 \| 0.513 \| \| Prime [Irregular] \| -0.04 \| -0.21 – 0.13 \| 0.628 \| \| Miniblockhalf [Second] \| 0.00 \| -0.17 – 0.17 \| 0.984 \| \| Prime [Silence] × Miniblockhalf [Second] \| 0.08 \| -0.17 – 0.32 \| 0.538 \| \| Prime [Irregular] × Miniblockhalf [Second] \| -0.11 \| -0.35 – 0.13 \| 0.364 \| \| **Random Effects** \| \| \| \| \| σ^2^ \| 0.33 \| \| \| \| τ_00_ _Subject_ \| 0.44 \| \| \| \| ICC \| 0.57 \| \| \| \| N _Subject_ \| 89 \| \| \| \| Observations \| 534 \| \| \| \| Marginal R^2^ / Conditional R^2^ \| 0.004 / 0.569 \| \| \| |
| --- | --- | --- | --- | --- | --- | --- | --- | --- | --- | --- | --- | --- | --- | --- | --- | --- | --- | --- | --- | --- | --- | --- | --- | --- | --- | --- | --- | --- | --- | --- | --- | --- | --- | --- | --- | --- | --- | --- | --- | --- | --- | --- | --- | --- | --- | --- | --- | --- | --- | --- | --- | --- | --- | --- | --- | --- | --- | --- | --- | --- |
| **Table 11:** **Summary of fixed effects obtained using the summary(model) function of the lme4 package in R. Model: D' ~ Prime * Miniblockhalf + 1\|Participant on data from Experiment 2** |
