# Supplementary material for: Not primed to agree? Short or no effect of rhythmic priming on typical adults processing number agreement
Source: Front Psychol. 2025 Jun 13;16:1512267. doi: 10.3389/fpsyg.2025.1512267 (PMC12204084; doi:10.3389/fpsyg.2025.1512267)
Supplement: Supplementary file 11 [file Table_10.docx]

| \|  \| **Sum Sq** \| **Mean Sq** \| **NumDf** \| **DenDF** \| **F value** \| **Pr(>F)** \| \| --- \| --- \| --- \| --- \| --- \| --- \| --- \| \| Prime \| 0.9544849 \| 0.4772424 \| 2 \| 440 \| 1.4296988 \| 0.2404908 \| \| Miniblockhalf \| 0.0138565 \| 0.0138565 \| 1 \| 440 \| 0.0415105 \| 0.8386498 \| \| Prime:Miniblockhalf \| 0.7859581 \| 0.3929791 \| 2 \| 440 \| 1.1772669 \| 0.3090883 \| |
| --- | --- | --- | --- | --- | --- | --- | --- | --- | --- | --- | --- | --- | --- | --- | --- | --- | --- | --- | --- | --- | --- | --- | --- | --- | --- | --- | --- | --- |
| **Table 12**: **Main effects and interactions obtained using the anova(model) function in R.**  **Model: D' ~ Prime * Miniblockhalf + 1\|Participant on data from Experiment 2** |
