# Supplementary material for: Not primed to agree? Short or no effect of rhythmic priming on typical adults processing number agreement
Source: Front Psychol. 2025 Jun 13;16:1512267. doi: 10.3389/fpsyg.2025.1512267 (PMC12204084; doi:10.3389/fpsyg.2025.1512267)
Supplement: Supplementary file 12 [file Table_11.docx]

| \|  \| **dprime** \| \| \| \| --- \| --- \| --- \| --- \| \| *Predictors* \| *Estimates* \| *CI* \| *p* \| \| (Intercept) \| 1.73 \| 1.57 – 1.89 \| **<0.001** \| \| Prime [Silence] \| -0.16 \| -0.34 – 0.03 \| 0.090 \| \| Prime [Irregular] \| -0.13 \| -0.32 – 0.05 \| 0.155 \| \| **Random Effects** \| \| \| \| \| σ^2^ \| 0.39 \| \| \| \| τ_00_ _Subject_ \| 0.20 \| \| \| \| ICC \| 0.34 \| \| \| \| N _Subject_ \| 89 \| \| \| \| Observations \| 267 \| \| \| \| Marginal R^2^ / Conditional R^2^ \| 0.008 / 0.344 \| \| \| |
| --- | --- | --- | --- | --- | --- | --- | --- | --- | --- | --- | --- | --- | --- | --- | --- | --- | --- | --- | --- | --- | --- | --- | --- | --- | --- | --- | --- | --- | --- | --- | --- | --- | --- | --- | --- | --- | --- | --- | --- | --- | --- | --- | --- | --- | --- | --- | --- | --- |
| **Table 13**: **Summary of fixed effects obtained using the summary(model) function of the lme4 package in R. Model: D' ~ Prime + 1\|Participant on the first sentence after each prime in Experiment 2** |
