# Supplementary material for: Not primed to agree? Short or no effect of rhythmic priming on typical adults processing number agreement
Source: Front Psychol. 2025 Jun 13;16:1512267. doi: 10.3389/fpsyg.2025.1512267 (PMC12204084; doi:10.3389/fpsyg.2025.1512267)
Supplement: Supplementary file 14 [file Table_13.docx]

| \|  \| **dprime** \| \| \| \| --- \| --- \| --- \| --- \| \| *Predictors* \| *Estimates* \| *CI* \| *p* \| \| (Intercept) \| 2.70 \| 2.50 – 2.91 \| **<0.001** \| \| Prime [Silence] \| -0.05 \| -0.22 – 0.12 \| 0.550 \| \| Prime [Irregular] \| 0.00 \| -0.17 – 0.17 \| 1.000 \| \| **Random Effects** \| \| \| \| \| σ^2^ \| 0.21 \| \| \| \| τ_00_ _Subject_ \| 0.45 \| \| \| \| ICC \| 0.68 \| \| \| \| N _Subject_ \| 60 \| \| \| \| Observations \| 180 \| \| \| \| Marginal R^2^ / Conditional R^2^ \| 0.001 / 0.680 \| \| \| |
| --- | --- | --- | --- | --- | --- | --- | --- | --- | --- | --- | --- | --- | --- | --- | --- | --- | --- | --- | --- | --- | --- | --- | --- | --- | --- | --- | --- | --- | --- | --- | --- | --- | --- | --- | --- | --- | --- | --- | --- | --- | --- | --- | --- | --- | --- | --- | --- | --- |
| **Table 15:** **Summary of fixed effects obtained using the summary(model) function of the lme4 package in R. Model: D' ~ Prime + 1\|Participant on data from Experiment 3** |
